# Predicting population age structures of China, India, and Vietnam by 2030 based on compositional data

**DOI:** 10.1371/journal.pone.0212772

**Published:** 2019-04-11

**Authors:** Yigang Wei, Zhichao Wang, Huiwen Wang, Yan Li, Zhenyu Jiang

**Affiliations:** 1 School of Economics and Management, Beihang University, Beijing, China; 2 Business School, Shandong University, Weihai, Shandong, China; 3 School of Public Policy & Management, Tsinghua University, Beijing, China; Politecnico di Torino, ITALY

## Abstract

The changing population age structure has a significant influence on the economy, society, and numerous other aspects of a country. This paper has innovatively applied the method of compositional data forecasting for the prediction of population age changes of the young (aged 0–14), the middle-aged (aged 15–64), and the elderly (aged older than 65) in China, India, and Vietnam by 2030 based on data from 1960 to 2016. To select the best-suited forecasting model, an array of data transformation approaches and forecasting models have been extensively employed, and a large number of comparisons have been made between the aforementioned methods. The best-suited model for each country is identified considering the root mean squared error and mean absolute percent error values from the compositional data. As noted in this study, first and foremost, it is predicted that by the year 2030, China will witness the disappearance of population dividend and get mired in an aging problem far more severe than that of India or Vietnam. Second, Vietnam’s trend of change in population age structure resembles that of China, but the country will sustain its good health as a whole. Finally, the working population of India demonstrates a strong rising trend, indicating that the age structure of the Indian population still remains relatively “young”. Meanwhile, the continuous rise in the proportion of elderly population and the gradual leveling off growth of the young population have nevertheless become serious problems in the world. The present paper attempts to offer crucial insights into the Asian population size, labor market and urbanization, and, moreover, provides suggestions for a sustainable global demographic development.

## Introduction

**Fig 1:**
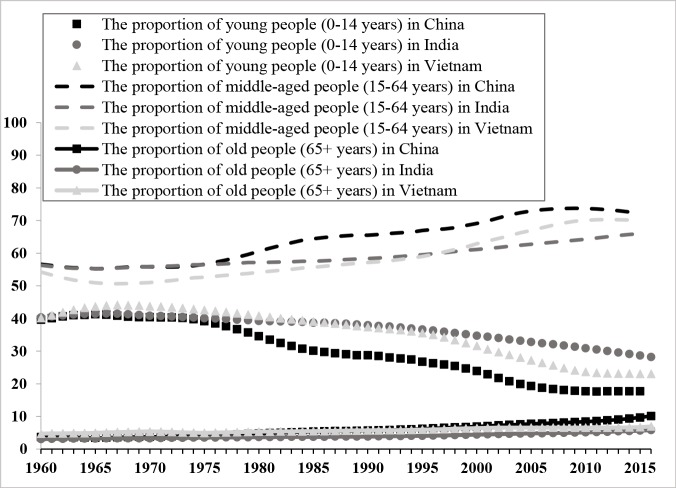
Historical changes of population age structures in China, India and Vietnam from 1960 to 2016.

Population age structure forecast is a research method that includes a comprehensive consideration of factors influencing development and a scientific calculation of population trend in the future [1]. Numerous researches have indicated that population age structure plays a decisive role in economic growth [2–10]. Since the supply of labor force and the saving rate continue to change over their life cycles, and given that their prolonged life span can contribute to the supply of labor force and saving rate, the change in population age structure can have a huge influence on a society’s economic performance. Fig 1 shows a description of the historical changes in population age structures from 1960 to 2016 in China, Vietnam, and India, in which it is shown clearly that the three countries differ significantly in this regard.

**Fig 2:**
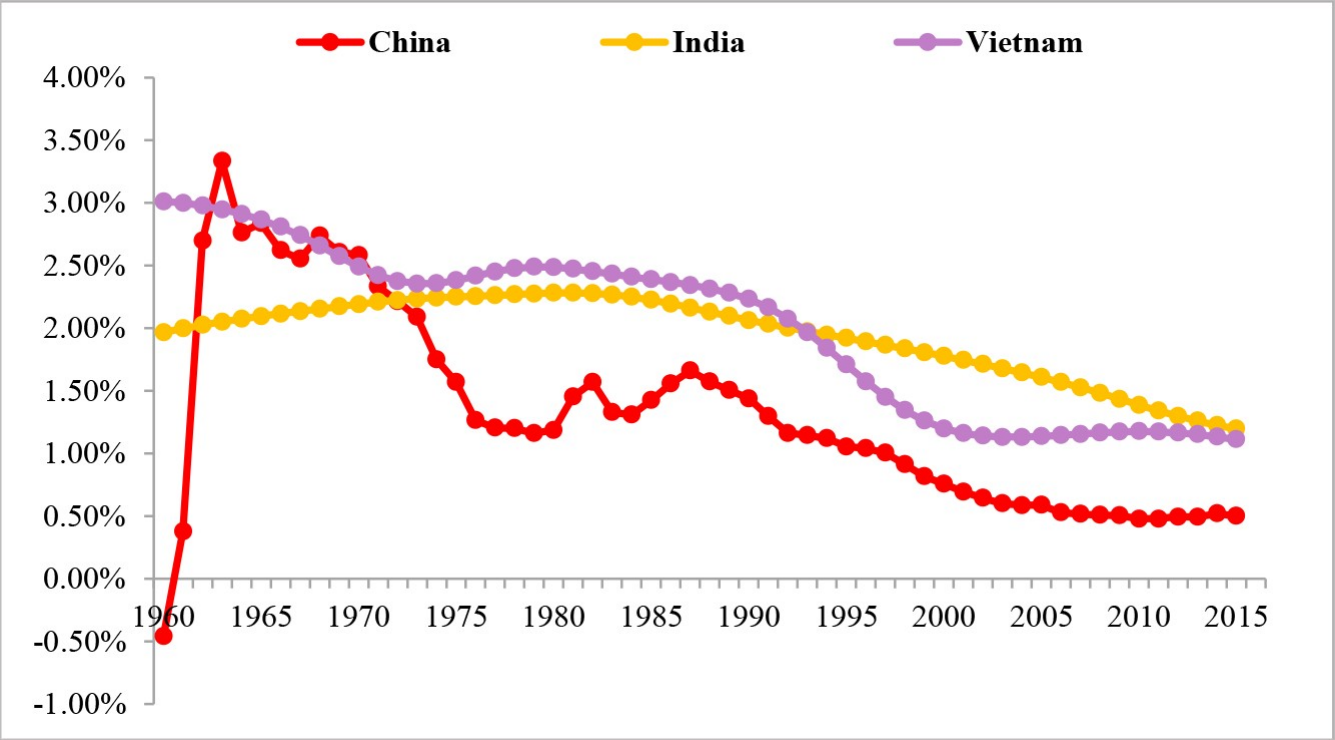
Natural population growth rates of China, India and Vietnam from 1960 to 2015. Data Source: World Bank (2018).

**Fig 3:**
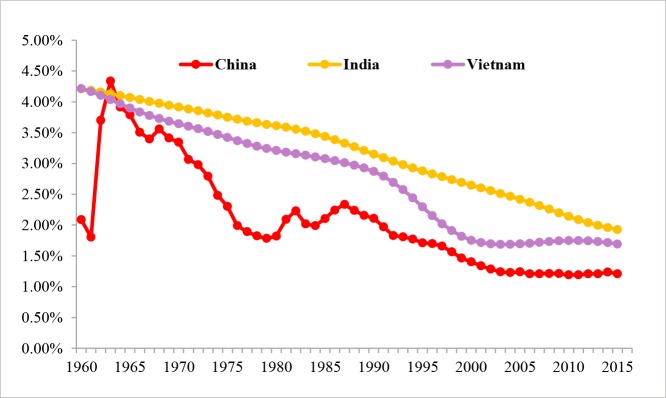
Birth rates of China, India and Vietnam from 1960 to 2015. Data Source: World Bank (2018).

The natural population growth rates and the birth rates of the three countries from 1960 to 2015 are depicted, respectively, in Figs 2 and 3. Overall, the populations of the three countries have started showing a declining trend. Before the 1970s, it used to be a prevailing opinion that the growth of population, the “population bomb” especially, might impede economic development. According to the UN, prior to the middle of the 1980s, the governments in 127 countries then across the globe in one way or another advocated birth control policies, covering 94% of the then population [11].

Considering the three countries in our study, China, having adopted the birth control policy at the end of 1960s, succeeded in progressing from the traditional stage (characterized by high birth rate, high death rate, and high natural growth rate) to the modern stage (characterized by low birth rate, low death rate, and low natural growth rate) of population transition within only three decades, achieving a spectacular miracle of demographic development [12]. However, alongside the above achievements, such problems as getting old before getting rich and newborn sex ratio imbalance are also confronting China, driving it towards an aging society despite its inadequate level of economic development [13, 14]. In recent years, the Chinese government is attempting to curb the negative influences such as the shortage of labor and “getting old before getting rich” arising from previous birth control policy through the implementation of an overall second-child policy. This policy aims to reduce the dependency burden of young adults by improving the age structure of families and the whole population, and furthermore, to promote harmonious demographic and economic development. As is well known, rapid population growth once provided ample workforce for China’s economic development [15], but as the age structure of population changes and the first demographic dividend diminishes, the massive population is gradually becoming a huge burden in recent year[16].

India, one of the most populous countries in the world second only to China, is also a major agrarian economy, with a large rural population and a huge wealth gap. Some researchers found from their comparative studies on China’s and India’s populations that in the next fifteen to twenty years, India will surpass China as the richest country in labor force resources, and furthermore exceed China in terms of national competitiveness [17]. In contrast, China, due to its shortage of labor force resources, will lose its edge in international competition. But the ever-expanding Indian population has nevertheless brought tremendous pressure on both national and global economy, resources, and environment. Unlike China, the Indian population is characterized by high marriage rate, young age at marriage, high birth rate, high illiteracy rate, preference for son, low urbanization rate, and dense population in big cities. As the second most populated country in the world, as well as a developing country with the fastest growing economy, India is populated on a land area only one-third the size of China by as many as 1.35 billion people, and that number is still increasing, which has resulted in great conflicts between the demographic and economic development, and therefore attracted attention, both domestic and overseas.

Vietnam, which is affected by uneven distribution of population, expanding population in urban area, and unbalanced sex ratio, is also confronted with grave challenges in demographic management. For a long time, in an effort to tackle its huge population size and high birth rate, the government of Vietnam had implemented certain policies of population control. For instance, Vietnam had once proposed objectives and measures for its population issue in the 21^st^ century, which includes establishing population and birth control commissions from the central to local level, to initiate and promote birth control campaigns, in order to curb population growth [18]. Although Vietnam has already made its way to an aging society by international standards, its old-age dependency ratio is nevertheless in a gradual decline, whereas other parts of the world see an upward trend in the same ratio, which is a favorable circumstance for Vietnam’s development. At the same time, Vietnam’s child dependency ratio, also in a drastic decline, descended from its historical high (87.67% in 1967) to a new low (32.82% in 2011), a drop of 54.85% [19]. As seen from the aforementioned data, Vietnam has reaped its harvest of demographic dividend. There is no denying that the aging problem is a global challenge, and even more so for a developing country like Vietnam. Up to now, Vietnam has not yet included policies for tackling the aging problem into its long-term strategic plan of national economic development, nor has it established a social pension system and an elderly service system. Furthermore, the country is seriously lagging behind in aging problem research.

**Fig 4:**
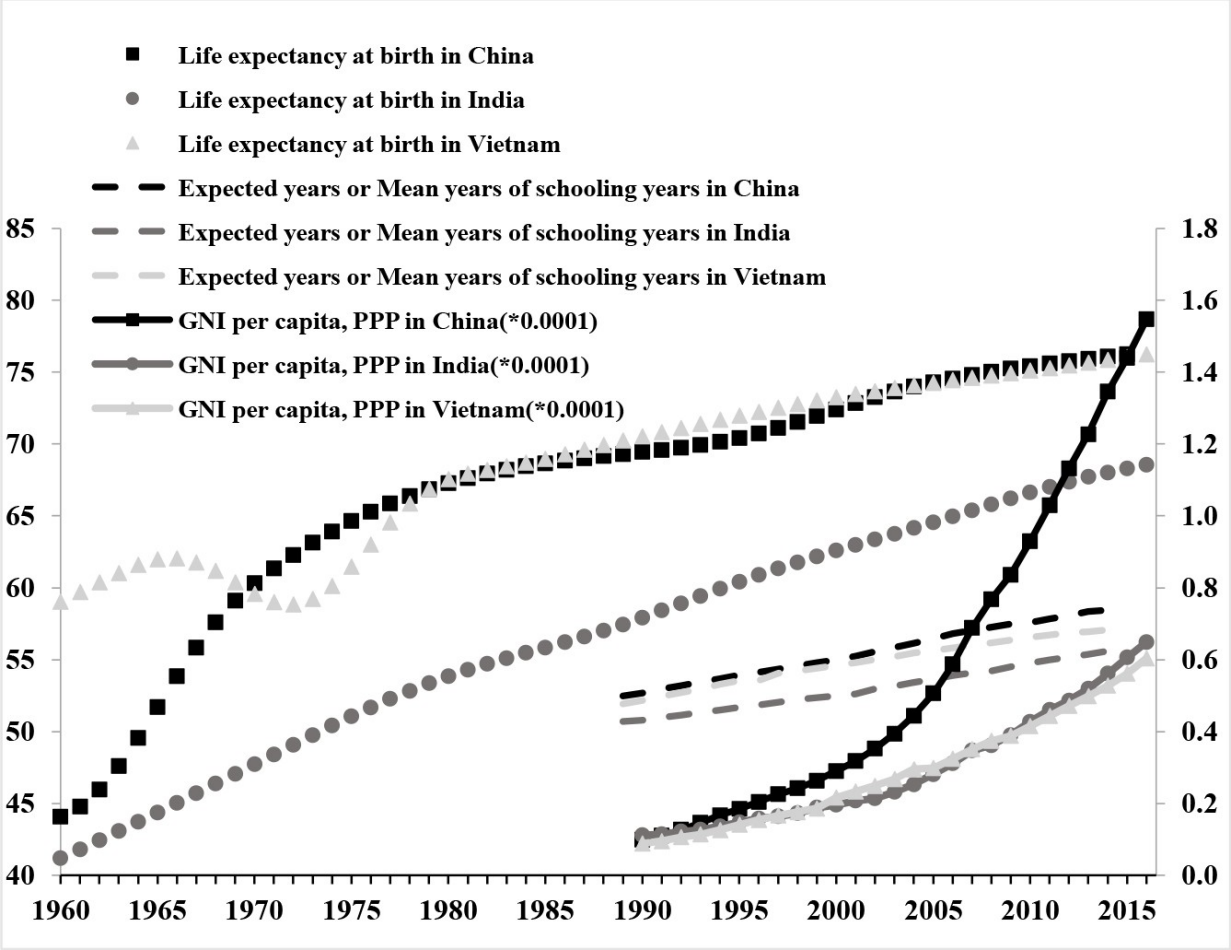
Human development index (HDI) of China, India & Vietnam from 1960 to 2015. Data source: Human Development Report of United Nations Human Development Program and World Bank, http://hdr.undp.org/en/composite/HDI.

A country’s economic and social development is, to some degree, determined by changes in its population structure. As illustrated in Fig 4, as changes take place in the population structure of the three countries under consideration, the economic and social indexes also change significantly. The present study has analyzed these indexes on the basis of the human development index (HDI). First, the longevity of the population has registered an upward trend since 1960. Meanwhile, the life expectancies in both China and Vietnam (76.25 years) have exceeded that of India (68.25 years), which is a powerful indicator of China and Vietnam’s advantages over India in terms of life quality, living conditions, and medical care, among others. Second, in an attempt to measure the national education status, the expected years of schooling or the average years of schooling is selected.

Many methods and models are employed for population projection at present, including the ARIMA model, logistic regression, the Leslie matrix, exponential smoothing model, and the Grey System [20–24]. The abovementioned models are mainly based on components of population change and the cohort component method [25–27], as well as the socioeconomic model relating population growth to economic development [28, 29], each with its own strengths and weaknesses. Although these models were comprehensible and verifiable, they are nevertheless defective in many ways. For instance, the ARIMA model is low in prediction accuracy and given to frequent errors. The logistic regression is prone to statistical structural problems such as null unit and multicollinearity. The exponential smoothing model is prone to frequent hysteresis quality. The Grey System model fails to take into consideration the influence load and that the original sequence is required to be non-negative, monotonic, and in accordance with the exponential rule, which is its greatest weakness.

This study employs compositional data analysis models to predict the dynamic changes of population structures in China, India, and Vietnam from 2016 to 2030. Compositional analysis data is commonly used to describe the internal structure of a system, such as investment structure, industrial structure, and consumption structure [30]. Compositional data prediction model is employed here on the basis of historical data and derivation of evolutionary trend with the aim of predicting the national population structure. Previously, what the traditional solution did is to derive the predictive ratio separately, which was likely to result in incomprehensible prediction outcomes. For example, if the summation of prediction ratio did not equal to 1, the outcome would become unpredictable. On the contrary, the employment of compositional data can avoid such flaws and achieve comprehensible and reasonable prediction of internal structure.

The remaining part of this paper is divided into four sections. Section 2 provides an extensive literature review on population prediction. Section 3 describes research methods and data resources. Section 4 presents the research results and discussion. Section 5 concludes the present study and offers suggestions for China, India, and Vietnam.

## Literature review

The size, structure and characteristics of population play a pivotal role in national economic policy-making. To study the errors of population prediction, statisticians and demographers has developed various methods of population prediction in the past two decades, such as ex-post evaluation, probabilistic Bayesian approach, Time Series Model [31–33] and Age Structure Prediction Model [34, 35]. An extensive literature review on population prediction and population age structure prediction is conducted to summarize the advantages and disadvantages of relevant theories and lay a solid theoretical foundation for this study.

### Population prediction methods based on statistical principles

The research method of population prediction originated in Britain. As early as 1697, Professor GKing, a prestigious sociologist, used a mathematical theory model to predict the national population structure. Although the prediction interval was relatively short, his mathematical method laid the foundation for future studies [36]. Since then, with the continuous development of demography, various population prediction models have emerged, which mainly include the Malthus model [37–39], Logistic model [40–42], Markov chain model [43–45], Keyfitz Matrix model [46], Autoregressive Integrated Moving Average (ARMA) model [47, 48], Leslie model [34, 49–52] and Bayesian model [53–55]. In 1798, Malthus, a British demographer and political economist, proposed the famous exponential model (Malthus model) in his famous work *An Essay on the Principle of Population* [56]. The model indicates that the population would display the characteristic of exponential growth when there is no restriction.

In the middle of the 19th century, Phil Haas, the Dutch biologist, proposed a formula which illustrates the relationship between population size, population growth rate and environmental carrying capacity, on the basis of the Malthus model and the restriction on the population imposed by the environment and resources was also taken into consideration. It is called the Logistic formula [57]. Both Logistic formula model and the Malthus index model are based on the population in a past year, and the fixed population growth rate is introduced to predict the future population in an enclosed environment (without consideration of the factor of population migration). However, in practice, this model is only an approximation algorithm, and there are still many shortcomings.

In the early 20th century, Andre Markov applied the transition probability model to the field of population prediction [43–45]. Since this method can effectively predict the future population with a small number of population data in a short term. This method has a greater advantage especially when the historical population data are incomplete or inaccurate [58].

Nathan Keyfitz took the lead in proposing the idea of predicting population by matrix multiplication on the basis of previous studies, and created the matrix model [46]. By processing the population according to gender, age, fertility rate and survival rate respectively, the matrix was established to predict the development trend of future population.

In 1951, Peter Whitle proposed the famous Autoregressive Integrated Moving Average (ARMA) model [47]. With its simplicity and practicability, this model has become an important method for researching time series models [47]. Then, the model was introduced into the field of population prediction. It obtained good outcomes in short-term population prediction and was widely applied. However, this model is a linear model, while population growth is not necessarily linear. Particularly for long-term population growth, the characteristics of linear growth are not likely to be displayed. Therefore, this model will show a big deviation in long-term population prediction.

Patrick h. Leslie proposed the Leshelie matrix based on age structure and the Leslie matrix model [49, 50] by adding migration factors and improving the Keyfitz matrix model. By dividing the population into different age groups and taking the factors of population migration into account, this model can dynamically predict the age structure of the population and the changes in its number. Compared with the original Keyfitz matrix, this model has been significantly improved and has become the most commonly used model in the field of population prediction.

In recent years, a large number of papers which adopted the Bayesian method to predict population structure has been increasing. Bryant and Graham (2013) used the improved Bayesian method to predict the population, where the population data of New Zealand were adopted to create an accounting framework based on region, age, gender and time to estimate the trend of population growth [54]. This accounting model constrained the real value of population components. Meanwhile, the above method is also similar to the non-Bayesian method proposed by Smith et al. (2010) and Raymer et al. (2011) [59, 60]. Subsequently, Wheldon et al. (2013) reestimated and predicted the Bayesian model by modeling fertility rate, mortality rate, net migration rate and fully considering the different qualities of population figures attainable in the census [55]. However, this method does not include age profile in the system modeling, nor does it take the behavior change over time into account. On the basis of the Lee-Carter model, Wiśniowski et al. (2015) developed a completely integrated dynamic Bayesian method for predicting mortality rate, birth rate, migrant number, migration rate, and age structures [61]. The characteristics and differences of various models are shown in Table 1.

**Table 1:** Comparisons of prediction models based on statistical principles.

Model	Statistical Principle	Factors of Consideration	Pros	Cons
Malthus Model	Exponential Function	Base annual population and growth rate	Being simple and easily calculating	The factors of consideration were oversimplified
Logistic Population Growth Model	Exponential function	Base annual population, population growth rate, environmental carrying capacity	The environmental carrying capacity of population is taken into consideration	The factors of consideration were insufficient The carrying capacity of population is hard to measure
Markov Chain Model	Probability theory	The previous one of the current transition (regardless of the historical data)	Historical data are not needed to deal with the probability of random events	Ineffective when dealing with predictions related to the past The transition probability is hard to calculate and change
Keyfitz Matrix Model	Matrix multiplication	Gender, age, fertility rate and survival rate	A large number of factors is taken in to consideration and the age structure is predictable.	The influence of population migration is not taken into consideration Large number of requirements of data No concern of factors related to text and knowledge as economic development, population policies et al.
Autoregressive Integrated Moving Average Model	Autoregressive Integrated Moving Average Function	Historical population data	Being simple, practical, and suitable for short-term prediction	Big deviation in long-term prediction Lack of consideration of factors related to text and knowledge
Leslie Matrix	Matrix multiplication	Gender, age, fertility rate, survival rate and population migration	Consideration of migrations	High demand on data precision Neglects of economic development and population policy factors
Bayesian Model	Improved Bayesian method	Region, age, gender, time, fertility rate, mortality rate and net migration	The effect of empirical knowledge is taken into account.	No consideration of changing behavior as time lapses

In addition to the above-mentioned common models, in recent years, some scholars have used the Multilevel Functional Data Model [62], Double Exponential Smoothing Model (DES) to predict the population structure. For example, by using the population data of the UK from 1975 to 2009, scholars employed the Multilevel Functional Data Model to predict the population from 2010 to 2030 [62]. Alias et al. (2016) predicted the number of people in need of housing in Johor state with statistics obtained from the Malaysian statistics bureau, and then further compared the two methods of population prediction [63]. It has been discovered that the Double Exponential Smoothing model (DES) is slightly better than the Autoregressive Integrated Moving Average model (ARIMA) [63].

### Intelligent population prediction method

In 1943, McCulloch, the psychologist, and Pitts, the logician, first proposed the concept of artificial neural network, which had a revolutionary impact on the field of statistical prediction and intelligent prediction models had gradually become a trend [64]. The intelligent prediction method has attracted the attention of scholars who study population prediction and they introduced it into this field.

In addition to neural network method, grey theory has also been rapidly developed and widely applied in the field of population prediction [65, 66]. Grey theory was first put forward by Professor Deng Julong, a famous Chinese scholar, in 1982. Based on this, GM(1,1) was established [65].

The grey model only needs to consider its own time series to find useful information, discover and understand the internal laws of things to make prediction, which significantly simplifies the amount of calculation work. It has been rapidly developed and widely used in the population prediction field. There are also certain limitations of the grey model: (1) when the dispersion degree of population data is large, the prediction accuracy will be reduced; (2) considerable deviation is shown in long-term population prediction. In addition, with the advancement of machine learning, experts and scholars have constructed Least Squares Support Vector Machine (LS-SVM) to predict age-specific fertility rate and age-specific male/female mortality rate [67].

Compared with the traditional population prediction model based on the principles of statistics, the intelligent population prediction model has the following prominent advantages: (1) it can process a large number of quantitative influencing factors simultaneously, while the driving factors processed by the statistical prediction model are relatively limited; (2) the accuracy requirement of data is lower than that of statistical prediction models; (3) it can deal with some influencing factors concerning textual knowledge that exert important influences on population growth. However, intelligent prediction models such as neural networks are not as stable as statistical prediction models in terms of the model structure, and it is more difficult to operate intelligent prediction models than statistical prediction models (see Table 2).

**Table 2:** Comparisons and analysis of the four prediction models.

Type of Model	Theoretical Basis	Structure Stability	Factors of Consideration	Data requirement	Data Processing Method	Prediction Accuracy	Operation Difficulty	Time Span Covered by Prediction	Maturity
Traditional Models	Statistical analysis theories	Stable relationship between variables	Relatively less	High accuracy complete data and textual data	Observed data are used in formula for calculation.	Fine accuracy in short-term prediction, certain deviation in long-term prediction	Operation is relatively simple.	Applicable for both short-term and long-term predictions, but better outcomes in short-term predictions.	Long history and mature
Neural Network	Artificial neural network theory, intelligent technology	Changing structure model	Various factors can be taken into consideration, including statistical and textual factors.	Statistics and textual data with lower accuracy requirement are usable.	Iterative observations of sample data, self-learning, establishing models	High accuracy in short-term prediction, certain deviations in mid-term and long-term prediction, but better than statistical models.	Operation is difficult and complex.	Extrapolation is good, and it is suitable for short-term, mid-term, and long-term predictions.	Developing
Grey Model	Grey Theory	Alterable Structure	Mainly based on statistical factors	Complete data are not required	New sequences are created by adding original data. New sequences are analyzed and figured out the laws.	High accuracy in short-term prediction, and big deviation in long-term prediction.	Relatively easy, mainly through adding layer by layer.	Suitable for short-term prediction	Developing
SVM model	Machine learning	Comparatively stable structure	Selection and optimization of data penalty term and kernel function mainly	Small sample	When linearly separable, it is converted to convex quadratic programming solution. When linearly inseparable, the appropriate kernel function is selected to map the training data to an eigenspace.	High accuracy in short-term and medium-term prediction	It is more difficult and suitable kernel function needs to be found.	Suitable for short-term and medium-term prediction	Developing

Overall, with the progress over time, population prediction methods have gradually developed from the most primitive manual calculation and pure theoretical models to population probability and age structure prediction models that rely on complex mathematical models and advanced science and technology. The accuracy of population prediction has been constantly improved. Besides, scholars have combined population prediction problems with many issues in reality, and some studies that are practical and interesting have emerged. On the basis of the existing research, this paper intends to take three populous countries including China, India and Vietnam as examples to predict the population by 2030 through improving and comparing the existing research methods, so as to provide corresponding reference support for global population development and policy-making.

## Research methods and data

**Fig 5:**
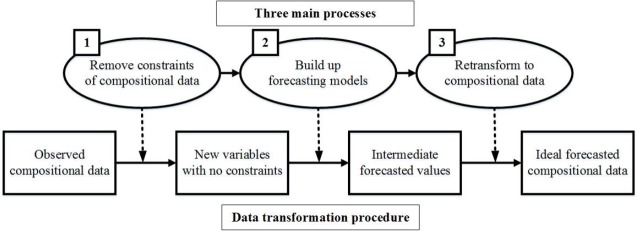
Procedures for conducting forecasting models for compositional data.

The compositional data describe the intrinsic structural information of an integrated system [68], for instance, the population proportions of three countries discussed in this paper. As shown in Fig 5, the procedure for using forecasting models for compositional data contains three key processes. First, the observed compositional data are transformed to some new variables that have no constraints. Second, traditional forecasting models can be conducted on these transformed variables so as to forecast the intermediate values. Finally, these intermediate values are then retransformed back to the ideal forecasted values of the compositional structure.

In mathematical notation, any *D*-part compositional data is expressed as a vector, ***x*** = [*x*_1_,*x*_2_,…,*x*_*D*_], which satisfies the unit-sum constraint, $\sum_{d=1}^{D}x_{d}=1$, and the positive constraint, 0≤*x*_*d*_≤1(*d* = 1,2,…,*D*).

We deal with the time series of compositional data, {***x***^(*t*)^,*t* = 1,2,…,*T*}, where the compositional observation at time *t* is $x^{\left(t\right)}=[x_{1}^{\left(t\right)},x_{2}^{\left(t\right)},\ldots ,x_{D}^{\left(t\right)}]$. We aim at applying forecasting models to these observed compositional data and deriving the forecasted compositional data value at time *T*+*k*, denoted by ***x***^(*T*+*k*)^.

### Transformation method for compositional data

The constraints in compositional data result in the inapplicability of most traditional forecasting models. Therefore, the following data transformation methods have been proposed to completely (or partly) remove the constraints in compositional data before modelling.

#### Linear combined component (LCC) method

In the LCC method, one of the inner parts within a compositional data is regarded as a linear combination of the others [69]. For example, select the last inner part $x_{D}^{\left(t\right)}$. Then the forecasting model is constructed based on the other *D*−1 inner parts (i.e. $x_{1}^{\left(t\right)},\ldots ,x_{D-1}^{\left(t\right)}$) and the forecasted values of these parts $x_{d}^{\left(T+k\right)}$ (*d* = 1,2,…,*D*−1) can be obtained. Finally, the forecasted value of the linear-combined part $x_{D}^{\left(T+k\right)}$ can be expressed as

$$x_{D}^{\left(T+k\right)}=1-\sum_{d=1}^{D-1}x_{d}^{\left(T+k\right)}.$$ (1)

#### Isometric log-ratio transformation (ILR)

The main computation steps for forecasting compositional data series by employing an ILR transformation are as follows [70–72]:

1. For time *t* (*t* = 1,2,…,*T*), compute the vector $w^{\left(t\right)}=(w_{1}^{\left(t\right)},w_{2}^{\left(t\right)},\ldots ,w_{D-1}^{\left(t\right)})$ based on ***x***^(*t*)^ as
  $$w_{d}^{\left(t\right)}=\sqrt{\frac{d}{d+1}}∙\ln{\left(\prod_{i=1}^{d}x_{i}^{\left(t\right)}\right)}^{1/d}/x_{d+1}^{\left(t\right)}(d=1,2,\cdots ,D-1).$$ (2)
2. For each *d* = 1,2,…,*D*−1, conduct the forecasting model based on the time series $\left\{w_{d}^{\left(t\right)},t=1,2,\ldots ,T\right\}$ and compute the forecasted value $w_{d}^{\left(T+k\right)}$. Then, the whole vector ***w***^(*T*+*k*)^ is expressed as
  $$w^{\left(T+k\right)}=(w_{1}^{\left(T+k\right)},w_{2}^{\left(T+k\right)},\ldots ,w_{D-1}^{\left(T+k\right)}).$$ (3)
3. Compute the vector $v^{\left(T+k\right)}=(v_{1}^{\left(T+k\right)},v_{2}^{\left(T+k\right)},\ldots ,v_{D}^{\left(T+k\right)})$ using ***w***^(*T*+*k*)^ as
  $$v_{d}^{\left(T+k\right)}=\sum_{i=d}^{D}\frac{1}{\sqrt{i(i+1)}}w_{i}^{\left(T+k\right)}-\sqrt{\frac{d-1}{d}}w_{d-1}^{\left(T+k\right)}(d=1,2,\ldots ,D),$$ (4)
  where $w_{0}^{\left(T+k\right)}$ and $w_{D}^{\left(T+k\right)}$ are set to be 0.
4. Finally, compute the forecasted compositional data ***x***^(*T*+*k*)^ as
  $$x^{\left(T+k\right)}=\left[\frac{v_{1}^{\left(T+k\right)}}{\sum_{d=1}^{D}v_{d}^{\left(T+k\right)}},\frac{v_{2}^{\left(T+k\right)}}{\sum_{d=1}^{D}v_{d}^{\left(T+k\right)}},\ldots ,\frac{v_{D}^{\left(T+k\right)}}{\sum_{d=1}^{D}v_{d}^{\left(T+k\right)}}\right].$$ (5)

#### Dimension Reduction Approach through Hyperspherical Transformation (DRHT)

The algorithm for conducting a forecasting model via DRHT can be summarized as follows [73]:

1. For time *t* (*t* = 1,2,…,*T*), compute the vector $\theta^{\left(t\right)}=(\theta_{2}^{\left(t\right)},\theta_{3}^{\left(t\right)},\cdots ,\theta_{D}^{\left(t\right)})$ based on ***x***^(*t*)^ as
  $$\left\{ \begin{array}{l} \theta_{d}^{\left(t\right)}=\arccos\sqrt{x_{d}^{\left(t\right)}},d=D,\\ \theta_{d}^{\left(t\right)}=\arccos\left(\frac{\sqrt{x_{d}^{\left(t\right)}}}{\prod_{i=d+1}^{D}\sin\theta_{i}^{\left(t\right)}}\right),d=D-1,D-2,\cdots ,2. \end{array}\right.$$ (6)
2. For each *d* = 2,3,…,*D*, construct the forecasting model based on the time series $\left\{\theta_{d}^{\left(t\right)},t=1,2,\ldots ,T\right\}$ and forecast the component value $\theta_{d}^{\left(T+k\right)}$. Then, the whole vector ***θ***^(*T*+*k*)^ is expressed as
  $$\theta^{\left(T+k\right)}=(\theta_{2}^{\left(T+k\right)},\theta_{3}^{\left(T+k\right)},\cdots ,\theta_{D}^{\left(T+k\right)}).$$ (7)
3. Finally, compute the forecasted compositional data ***x***^(*T*+*k*)^ from ***θ***^(*T*+*k*)^ as $x^{\left(T+k\right)}=[x_{1}^{\left(T+k\right)},x_{2}^{\left(T+k\right)},\cdots ,x_{D}^{\left(T+k\right)}]$, where
  $$\left\{ \begin{array}{l} x_{d}^{\left(T+k\right)}={\left(\prod_{i=2}^{D}\sin\theta_{i}^{\left(T+k\right)}\right)}^{2},d=1,\\ x_{d}^{\left(T+k\right)}={\left(\cos\theta_{d}^{\left(T+k\right)}∙\prod_{i=d+1}^{D}\sin\theta_{i}^{\left(T+k\right)}\right)}^{2},d=2,3,\cdots ,D-1,\\ x_{d}^{\left(T+k\right)}={\left(\cos\theta_{d}^{\left(T+k\right)}\right)}^{2},d=D. \end{array}\right.$$ (8)

The abovementioned methods describe the forecasting procedure (Step 2 in Fig 5). The advantages and disadvantages of the abovementioned methods for compositional data are described below.

1. The LCC method is intuitive and easy to perform. However, owing to the cumulative error, the results obtained by the LCC method may be uninterpretable and even paradoxical. For example, the inner parts within the forecasted compositional data are beyond the range of 0 to 1 [70].
2. The ILR transformation provides a good solution for most types of compositional data. Yet, this method is based on a relatively restrictive assumption, i.e., all the inner parts are supposed to be strictly positive [73].
3. The DRHT method seems more flexible, since it allows some inner parts to be zero. However, this method still does not allow for the existence of an inner part with the value of 1.

### Forecasting model

Many traditional forecasting methods can be adapted for the forecasting procedure using the observed compositional data that can be processed by the methods discussed in Section 3.1. The feasible approaches include the autoregressive integrated moving average model (ARIMA), exponential smoothing model (ETS), vector autoregressive model (VAR), and neural network nonlinear autoregressive model for time series (NNETTS). These popular forecasting models for time series are discussed in the following paragraphs.

#### Autoregressive integrated moving average

The ARIMA model is proposed by Box et al. [74] and is one of the most popular time-series forecasting techniques [75] Considering observations as the random consequences in time order, the ARIMA model captures the features of the latent random consequence and then the fitted or forecasted values are obtained from the constructed model.

#### Exponential smoothing

The ETS model is another common time-series forecasting method [76]. It assumes that the trend of the time series is stable and regular. In the ETS model, the forecasted values at a certain time can be derived by a weighted average of the previous observations. The single, second, and cubic ETS methods are three traditional ETS methods.

#### Vector autoregressive

The VAR model is one of the most widely used time-series models for analysing and forecasting multiple indicators [77]. This method regards the model as a function of the lagged time-series values of all the endogenous variables. Under some conditions, multivariate moving average (MA) and autoregressive moving average (ARMA) models can also be reformulated to the VAR model.

#### Neural network method for time series

The NNETTS model regards the time series as an input–output system determined by a nonlinear mechanism [78], which can better reveal the correlation of nonlinear time series in the delay state space. Specifically, in this study, the NNETTS model was implemented as a single-hidden layer nonlinear autoregressive model with related parameters, that is,

$$x_{t+h}=\beta_{0}+\sum_{j=1}^{D}\beta_{j}g(\gamma_{0j}+\sum_{i=1}^{m}\gamma_{ij}x_{t-i+1}),$$ (9)

where *x*_*t*+*h*_ denotes the *h*-ahead forecast, *D* and *m* denote the numbers of hidden units and lagged variables, respectively, and *β*_0_, *β*_*j*_ and *γ*_*ij*_ (*i* = 0,1,…,*m*;*j* = 1,2,…,*D*) are coefficients to be estimated, and *g*(∙) denotes the activation function.

The advantages and disadvantages of these four forecasting methods are summarised as follows:

1. The ARIMA model is widely used as a reliable forecasting method in general, with its consolidated theoretical underpinnings. However, this method requires the amount of the observed data to be large enough to capture the information of the latent model.
2. The ETS model has a relaxed data requirement, which can be conducted with a small sample size. However, the model has apparent disadvantages. Since the forecasting values at certain times are subjective to the closest several data in time order, the ETS method is more suitable for medium- and short-term forecasting.
3. The VAR model captures the linear interdependencies among multiple time series corresponding to all the inner parts of a compositional data. It allows for more than one evolving variable. All the variables in a VAR model are explained not only by their own lagged values but also by those of other variables. Nonetheless, the VAR model has relatively strong model assumptions and needs a large sample size [79].
4. The NNETTS model has been shown by numerous studies to have high forecasting accuracy in nonlinear time-series analysis. The downsides are that the NNETTS model needs a large amount of the sample size and takes a longer time to conduct.

Compared with the procedure for the traditional time-series forecasting, two processes for compositional data are of importance. One is the data transformation that removes the constraints in compositional data before modelling. This step succeeds in removing the influence of the constraints in compositional data during modelling. And the other is the data retransformation from the intermediate forecasted values back to the compositional data, which ensures that the final forecasted values satisfy the compositional structure. Through these two data transformations, most traditional forecasting methods can be adapted for the time series with compositional data.

### Developing a forecasting model

In this paper, we performed the out-of-sample forecasting to evaluate the efficiency of the alternative models. The whole observed compositional data, ***x***^(1)^,***x***^(2)^,…,***x***^(*Q*)^, are partitioned into two groups, i.e. the training data, ***x***^(1)^,***x***^(2)^,…,***x***^(*P*)^, and the forecasting data, ***x***^(*P*+1)^,***x***^(*P*+2)^,…,***x***^(*Q*)^. The training data are used to conduct the forecasting models and obtain the forecasted values associated with the forecasting data. Then the forecasting data and their forecasted values, denoted by ${\hat{x}}^{\left(P+1\right)},{\hat{x}}^{\left(P+2\right)},\ldots ,{\hat{x}}^{\left(Q\right)}$, are used to qualify the forecasting accuracy of the alternative model.

Two measures for evaluating the forecasting accuracy are introduced in this paper: the root mean squared error (RMSE) and mean absolute percent error (MAPE) for compositional data, denoted by CoDa-RMSE and CoDa-MAPE, respectively. They are defined by Aitchison (1982) as

$$CoDa-RMSE=\frac{1}{Q-P}\sum_{i=P+1}^{Q}d_{S}(x^{\left(i\right)},{\hat{x}}^{\left(i\right)})$$ (10)

and

$$CoDa-MAPE=\frac{1}{Q-P}\sum_{i=P+1}^{Q}\frac{d_{S}(x^{\left(i\right)},{\hat{x}}^{\left(i\right)})}{{\left\|x^{\left(i\right)}\right\|}_{S}}\times 100\%,$$ (11)

where $d_{S}(x^{\left(i\right)},{\hat{x}}^{\left(i\right)})$ and ‖***x***^(*i*)^‖_*S*_ are defined as

$$d_{S}(x^{\left(i\right)},{\hat{x}}^{\left(i\right)})=\sqrt{\sum_{d=1}^{D}{\left(\ln\frac{x_{d}^{\left(i\right)}}{gm(x^{\left(i\right)})}-\ln\frac{{\hat{x}}_{d}^{\left(i\right)}}{gm({\hat{x}}^{\left(i\right)})}\right)}^{2}},$$ (12)

$${\left\|x^{\left(i\right)}\right\|}_{S}=\sqrt{\sum_{d=1}^{D}{\left(\ln\frac{x_{d}^{\left(i\right)}}{gm(x^{\left(i\right)})}\right)}^{2}},$$ (13)

and gm(∙) denotes the geometric mean operator, e.g., $g(x^{\left(i\right)})={\left(\prod_{d=1}^{D}x_{d}^{\left(i\right)}\right)}^{1/D}$.

Compared with the traditional RMSE and MAPE, CoDa-RMSE and CoDa-MAPE are analogous definitions for compositional data [80]. Here, $d_{S}(x^{\left(i\right)},{\hat{x}}^{\left(i\right)})$ qualifies the absolute amount of the difference between the observed and forecasted values and ‖***x***^(*i*)^‖_*S*_ measures the magnitude of the observation. Lower values of CoDa-RMSE and CoDa-MAPE imply a more accurate and reliable forecasting model. The model with the lowest CoDa-RMSE and CoDa-MAPE was chosen as the best-suited compositional data forecasting model in this study.

### Data source and processing

This study used the yearly population data of China, India, and Vietnam from 1960 to 2016, sourced from the World Bank. Based on the age classification, there are three age periods: the young, middle-aged, and old populations. Thus, there are 57 observed population data for each country, along with three values corresponding to these three periods in each observation. Historical data for the populations of three countries are illustrated in Fig 1, and the main characteristics of the populations of three countries are summarized in Section 2.

In this study, the forecasting model of the three countries were conducted independently. To evaluate the forecasting performance of each alternative model and select the best-performing one for compositional data forecasting, this study partitioned the population data in two sets, namely the training and test ones. Specifically, for each country, the first 48-year (1960–2007) data were partitioned in the training set and the latest 9-year (1908–2016) data were partitioned in the test set. The model with the lowest CoDa-RMSE and CoDa-MAPE will be chosen as the best-performing forecasting model for compositional data.

## Results and discussion

Table 3 illustrates both the data transformation methods for compositional data and forecasting models. The test data (i.e., from 2008 to 2016) were used to measure the accuracy of all the alternative models. The forecasting performances of the family of NNETTS with different settings of parameters are summarized in Table 4, and the best ones related to three countries are then selected to compare with the other models in Table 5. Specifically, we chose NNETTS with *D* = 2 and *m* = 3 for China, *D* = 3 and *m* = 3 for India, and *D* = 2 and *m* = 2 for Vietnam in Table 5, respectively. To forecast the compositional data, two key processes are important. One is the transformation method for compositional data, and the other is the forecasting model. Rows and columns in Table 5 denote data transformation methods for compositional data and forecasting models, respectively, where the rows “Base” denote the corresponding conventional time series forecasting models and the sub-columns “RMSE” and “MAPE” denote the CoDa-RMSE and CoDa-MAPE values corresponding to the specified forecasting model for compositional data.

**Table 3:** Description of the data transformation methods for compositional data and forecasting models.

**Data transformation methods for compositional data**	
**LCC.Y**	LCC method regarding the proportion of young people as the linear combination
**LCC.M**	LCC method regarding the proportion of middle-aged people as the linear combination
**LCC.O**	LCC method regarding the proportion of old people as the linear combination
**ILR**	Isometric logratio transformation
**DRHT**	Dimension-Reduction approach through a Hyperspherical Transformation
**Forecasting models**	
**ARIMA**	Autoregressive integrated moving average model
**ETS**	Exponential smoothing model
**VAR**	Vector autoregressive model
**NNETTS**	Best neural network model for time series for the corresponding country

**Table 4:** CoDa-RMSE and CoDa-MAPE (in brackets) values of NNETTS combined with different data transformation methods in the test set.

Lagged variables	Method	Number of hidden units				
		*D* = 1	*D* = 2	*D* = 3	*D* = 4	*D* = 5
China						
*m* = 1	LCC.Y	0.19 (12.71%)	0.11 (7.41%)	0.09 (5.84%)	0.12 (7.75%)	0.19 (12.72%)
	LCC.M	0.13 (8.47%)	0.18 (12.14%)	0.23 (15.67%)	0.1 (6.89%)	0.13 (8.39%)
	LCC.O	0.09 (6.04%)	0.23 (15.13%)	0.33 (21.88%)	0.1 (6.72%)	0.09 (6.19%)
	ILR	0.09 (6.26%)	0.12 (8.1%)	0.11 (7.31%)	0.13 (8.58%)	0.12 (7.83%)
	DRHT	0.19 (12.53%)	0.12 (8.12%)	0.2 (13.55%)	0.11 (7.72%)	0.19 (12.63%)
*m* = 2	LCC.Y	0.22 (14.87%)	0.08 (5.09%)	0.1 (6.74%)	0.14 (9.4%)	0.09 (6.03%)
	LCC.M	0.1 (6.43%)	0.08 (5.49%)	0.11 (7.15%)	0.21 (14.2%)	0.26 (17.06%)
	LCC.O	0.17 (11.24%)	0.06 (4.2%)	0.07 (4.48%)	0.3 (20.31%)	0.41 (26.9%)
	ILR	0.13 (8.72%)	0.11 (7.46%)	0.08 (5.43%)	0.09 (6.2%)	0.11 (7.56%)
	DRHT	0.07 (4.81%)	0.08 (5.32%)	0.1 (6.94%)	0.09 (5.97%)	0.14 (9.36%)
*m* = 3	LCC.Y	0.47 (30.93%)	0.14 (9.73%)	0.07 (4.59%)	0.13 (8.91%)	0.06 (4.3%)
	LCC.M	0.43 (28.47%)	0.06 (3.94%)	0.08 (5.52%)	0.09 (6.17%)	0.1 (6.96%)
	LCC.O	0.13 (8.49%)	0.13 (8.67%)	0.11 (7.04%)	0.24 (16.3%)	0.17 (11.66%)
	ILR	0.12 (8.21%)	0.08 (5.5%)	0.12 (7.98%)	0.08 (5.59%)	0.1 (6.63%)
	DRHT	0.08 (5.61%)	0.06 (4.21%)	0.09 (6.33%)	0.1 (6.45%)	0.13 (8.61%)
India						
*m* = 1	LCC.Y	0.51 (28.57%)	0.21 (11.87%)	0.21 (11.91%)	0.16 (9.15%)	0.19 (10.35%)
	LCC.M	0.45 (25.39%)	0.2 (11.18%)	0.25 (13.83%)	0.18 (9.93%)	0.17 (9.73%)
	LCC.O	0.04 (2.47%)	0.32 (17.87%)	0.53 (29.63%)	0.47 (26.14%)	0.28 (15.97%)
	ILR	0.02 (0.98%)	0.33 (18.43%)	0.04 (2.01%)	0.03 (1.74%)	0.05 (2.9%)
	DRHT	0.09 (5.3%)	0.09 (5.15%)	0.19 (10.7%)	0.12 (6.48%)	0.09 (4.87%)
*m* = 2	LCC.Y	0.14 (7.94%)	0.14 (7.96%)	0.19 (10.45%)	0.1 (5.68%)	0.2 (10.95%)
	LCC.M	0.15 (8.14%)	0.16 (8.98%)	0.17 (9.76%)	0.12 (6.64%)	0.18 (10.21%)
	LCC.O	0.24 (13.46%)	0.32 (17.89%)	0.09 (5.06%)	0.27 (14.86%)	0.16 (9.07%)
	ILR	0.04 (2.44%)	0.02 (1.27%)	0.02 (1.24%)	0.04 (2.16%)	0.03 (1.41%)
	DRHT	0.03 (1.65%)	0.1 (5.42%)	0.19 (9.35%)	0.08 (4.33%)	0.12 (6.58%)
*m* = 3	LCC.Y	0.15 (8.53%)	0.05 (2.76%)	0.07 (4.06%)	0.13 (7.13%)	0.14 (7.8%)
	LCC.M	0.17 (9.38%)	0.03 (1.74%)	0.1 (5.64%)	0.11 (6.33%)	0.11 (6.09%)
	LCC.O	0.24 (13.51%)	0.24 (13.51%)	0.26 (14.78%)	0.13 (7.05%)	0.12 (6.55%)
	ILR	0.03 (1.51%)	0.03 (1.52%)	0.02 (0.93%)	0.16 (8.97%)	0.05 (2.74%)
	DRHT	0.2 (11.34%)	0.07 (4.03%)	0.28 (15.33%)	0.1 (5.44%)	0.08 (4.69%)
Vietnam						
*m* = 1	LCC.Y	0.18 (11.15%)	0.19 (11.21%)	0.06 (3.72%)	0.09 (5.48%)	0.09 (5.28%)
	LCC.M	0.12 (7.49%)	0.12 (7.3%)	0.5 (30.33%)	0.22 (13.01%)	0.15 (9.3%)
	LCC.O	0.28 (16.88%)	0.2 (12.24%)	0.94 (56.55%)	0.38 (22.89%)	0.39 (23.2%)
	ILR	0.11 (6.75%)	0.07 (4.5%)	0.11 (6.32%)	0.11 (6.85%)	0.08 (4.65%)
	DRHT	0.3 (17.9%)	0.15 (8.85%)	0.52 (31.26%)	0.17 (10.13%)	0.14 (8.58%)
*m* = 2	LCC.Y	0.14 (8.55%)	0.06 (3.69%)	0.2 (11.89%)	0.19 (11.36%)	0.14 (8.24%)
	LCC.M	0.06 (3.7%)	0.16 (9.84%)	0.08 (4.69%)	0.13 (8.12%)	0.15 (9.2%)
	LCC.O	0.27 (16.51%)	0.38 (22.86%)	0.14 (8.65%)	0.24 (14.7%)	0.56 (33.35%)
	ILR	0.24 (14.39%)	0.12 (7.09%)	0.07 (4.22%)	0.24 (14.39%)	0.07 (3.93%)
	DRHT	0.17 (9.99%)	0.22 (13.35%)	0.3 (18.05%)	0.16 (9.44%)	0.41 (24.46%)
*m* = 3	LCC.Y	0.13 (8.05%)	0.2 (12.03%)	0.08 (4.88%)	0.07 (4.39%)	0.41 (24.74%)
	LCC.M	0.13 (7.95%)	0.23 (13.62%)	0.09 (5.27%)	0.24 (14.56%)	0.19 (11.47%)
	LCC.O	0.11 (6.68%)	0.25 (14.84%)	0.14 (8.44%)	0.44 (26.47%)	0.4 (23.81%)
	ILR	0.24 (14.2%)	0.1 (6.14%)	0.1 (5.78%)	0.11 (6.76%)	0.24 (14.14%)
	DRHT	0.08 (4.61%)	0.31 (18.92%)	0.15 (8.94%)	0.23 (13.93%)	0.22 (13.54%)

**Table 5:** CoDa-RMSE and CoDa-MAPE comparisons of alternative forecasting models using compositional data.

	ARIMA		ETS		VAR		NNETTS	
	CoDa-RMSE	CoDa-MAPE	CoDa-RMSE	CoDa-MAPE	CoDa-RMSE	CoDa-MAPE	CoDa-RMSE	CoDa-MAPE
**China**								
**LCC.Y**	0.09	6.1%	0.09	6.1%	0.17	11.48%	0.14	9.73%
**LCC.M**	0.07	4.87%	0.09	6.17%	0.17	11.48%	0.06	3.94%
**LCC.O**	0.09	6.24%	0.09	6.17%	0.17	11.48%	0.13	8.67%
**ILR**	0.06*	3.94%*	0.08	5.38%	0.1	6.61%	0.08	5.5%
**DRHT**	0.08	5.67%	0.08	5.67%	0.1	6.91%	0.06	4.21%
**Base**	0.08	5.1%	0.09	6.09%	0.17	11.48%	0.15	10.22%
**India**								
**LCC.Y**	0.02	1.06%	0.01	0.83%	0.02	1.11%	0.07	4.06%
**LCC.M**	0.02	1.09%	0.01	0.83%	0.02	1.11%	0.1	5.64%
**LCC.O**	0.02	1.28%	0.01	0.84%	0.02	1.11%	0.26	14.78%
**ILR**	0.02	1.09%	0.01*	0.81%*	0.03	1.61%	0.02	0.93%
**DRHT**	0.02	1.07%	0.01	0.81%	0.02	1.11%	0.28	15.33%
**Base**	0.02	1.09%	0.02	0.9%	0.02	1.11%	0.08	4.81%
**Vietnam**								
**LCC.Y**	0.1	6.16%	0.08	5%	0.07	4.35%	0.06	3.43%
**LCC.M**	0.1	6.11%	0.08	5%	0.07	4.35%	0.17	10.22%
**LCC.O**	0.1	6.05%	0.08	4.99%	0.07	4.35%	0.62	37.12%
**ILR**	0.08	4.64%	0.07*	4.14%*	0.07	4.41%	0.24	14.33%
**DRHT**	0.09	5.69%	0.07	4.49%	0.07	4.27%	0.44	26.37%
**Base**	0.1	6.12%	0.07	4.1%	0.07	4.35%	0.07	4.51%

Rows “Base” indicate the results obtaining by the related conventional forecasting model for time series.

* indicates the best-performing forecasting models with lowest CoDa-RMSE and CoDa-MAPE values for three countries.

CoDa-RMSE and CoDa-MAPE are introduced as the key indicators for measuring the forecasting accuracy of the alternative models. As shown in Table 5, the forecasting performance of the alternative models is determined by both data transformation methods for compositional data (by row) and forecasting methods (by column). For the data transformation methods, the family of LCC methods have a large and uncertain volatility when combined with the NNETTS model; as, for Vietnam, the CoDa-RMSE value varies from 0.06 to 0.62 and the CoDa-MAPE value from 3.43% to 37.12%. By contrast, the ILR and DRHT methods perform well with more stable CoDa-RMSE and CoDa-MAPE values. For forecasting models, the CoDa-RMSE and CoDa-MAPE values of ARIMA, ETS, and VAR are relatively small. Moreover, comparing with the conventional time series forecasting approaches, the proposed compositional ones can obtain the more accurate results for the population age structure of three countries in general. This is reasonable since they utilize the correlation information between three periods of population ages.

**Fig 6:**
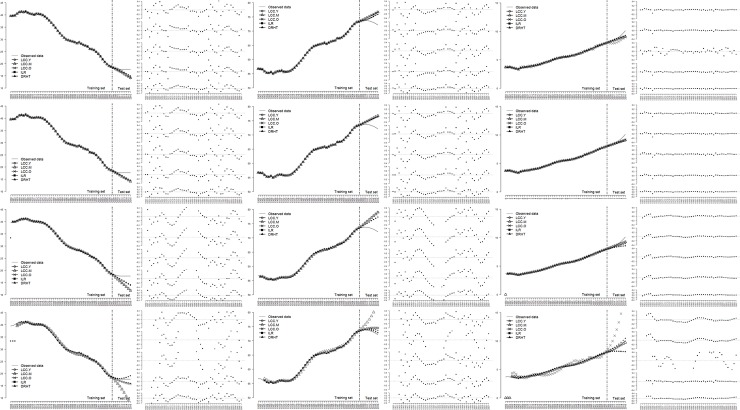
Comparisons of the forecasting performance of all the alternative models for China.

**Fig 7:**
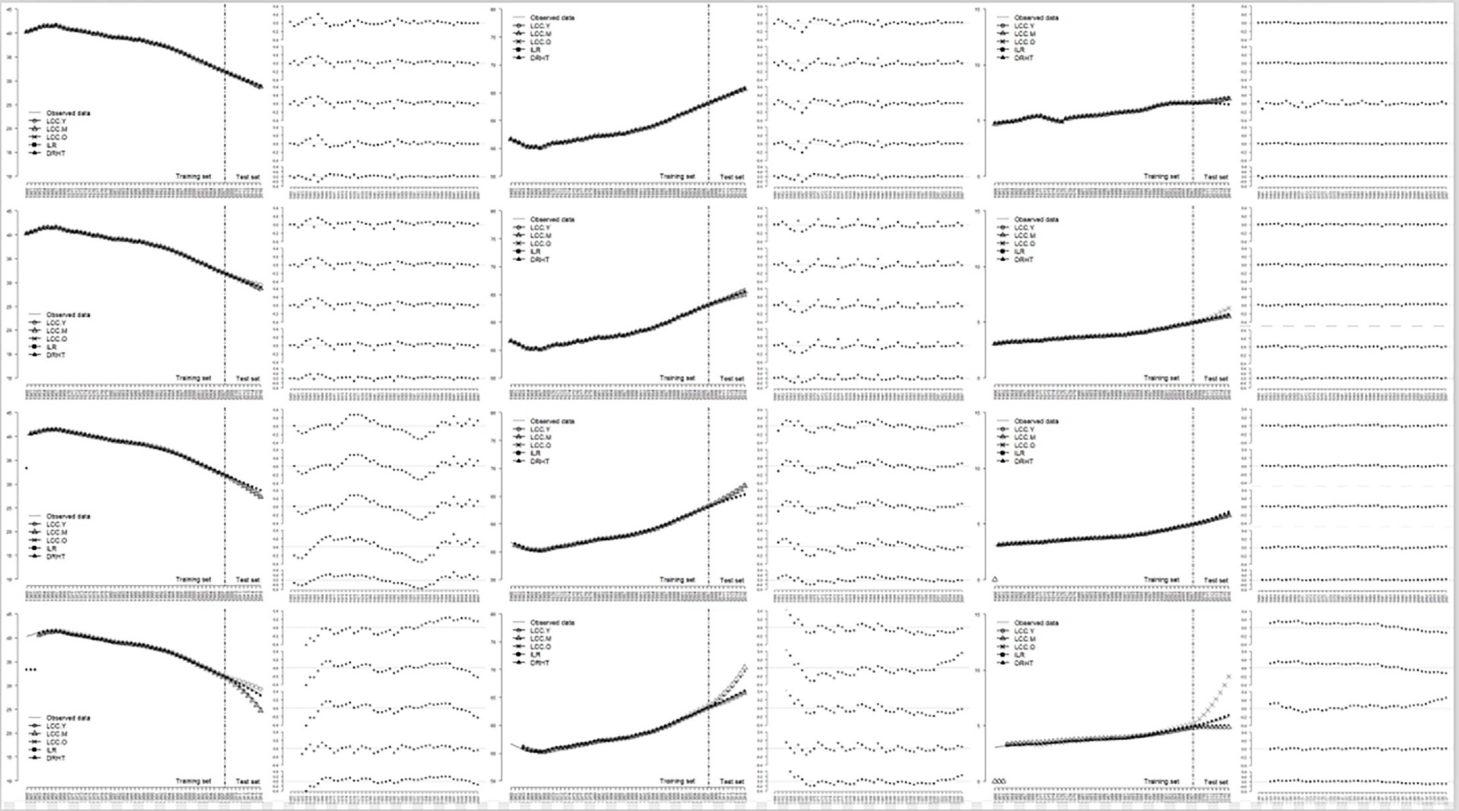
Comparisons of the forecasting performance of all the alternative models for India.

**Fig 8:**
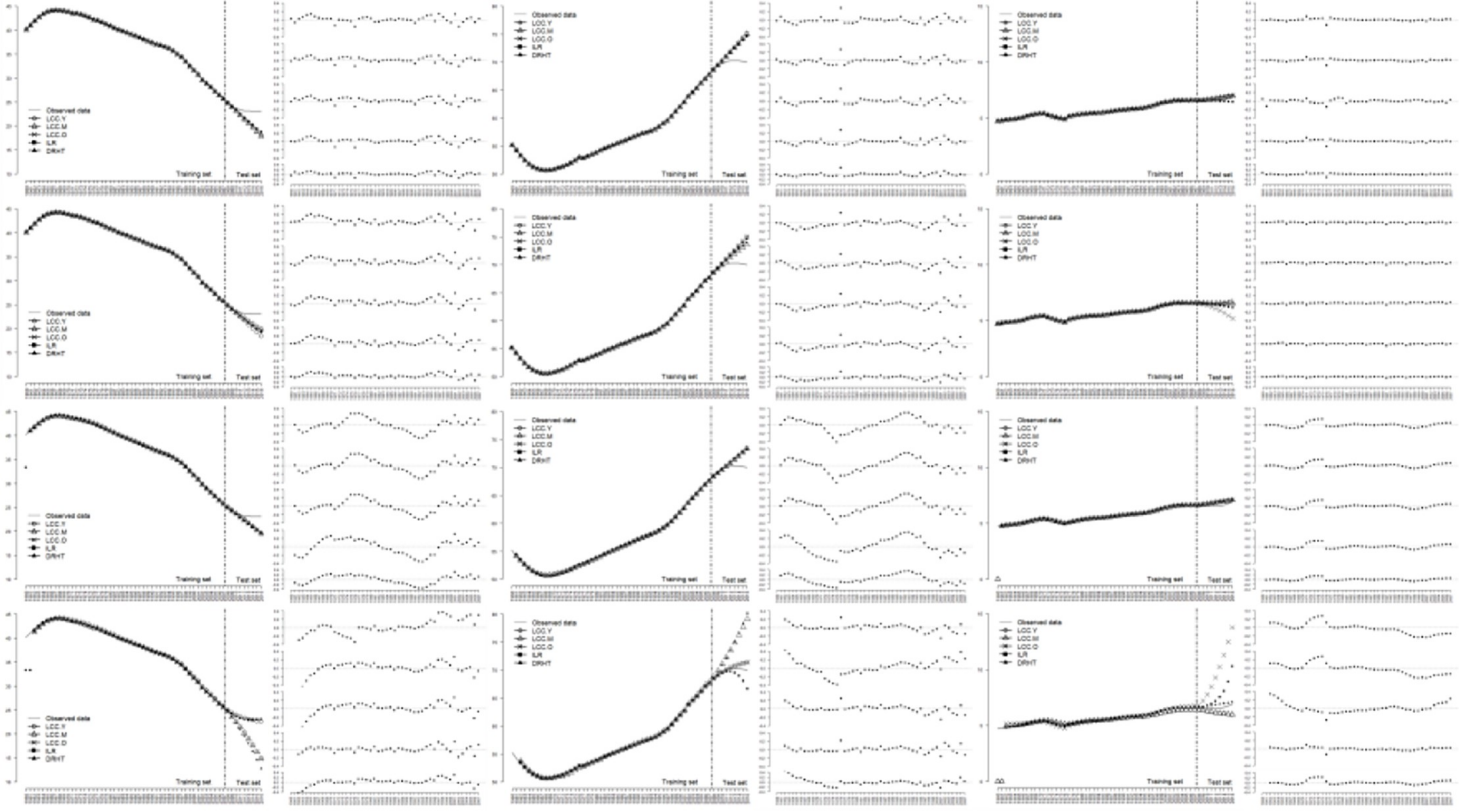
Comparison of the forecasting performance of all the alternative models for Vietnam.

Figs 6–8 illustrated the line charts of the estimated proportions of three age periods obtained by all the alternative models for three countries, along with the residual plots. For visualization, we considered 3 age periods for 3 countries separately and drew the estimated results obtained by 5 transformation methods in the same figure.

**Fig 9:**
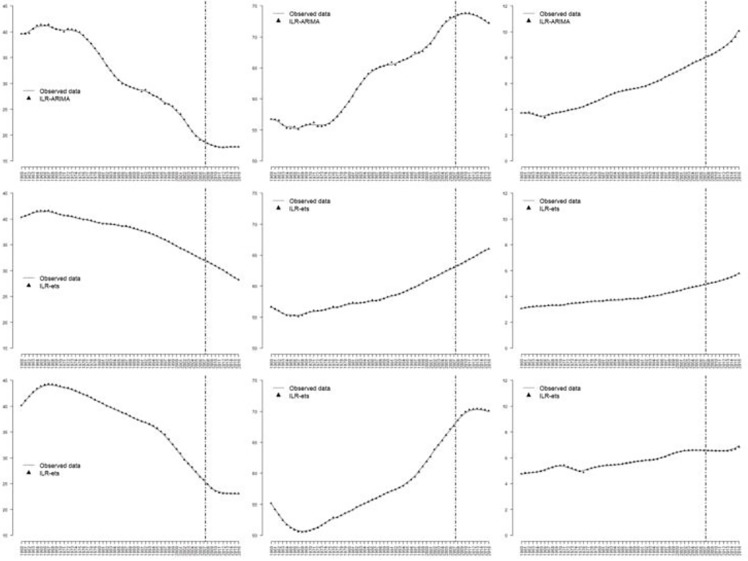
Performance of the best-performed forecasting models for three countries. Three columns indicate the estimated population age propositions of the young, middle-aged and old populations from left to right. Three rows indicate the related results for China, India and Vietnam from top to bottom. Detailed information refers to S1–S72 Figs.

For China, as shown in Fig 6, all the alternative models performed well in the training set. This result can also be concluded through the corresponding residual plots, where residuals for three age periods ranged from -0.4% to 0.4%. For the old population, most residuals approximated extremely to 0. Moreover, in the test set, all the models continued the downtrend of the young proportion and failed to detect the slowdown of the decline since 2008. This is common in the context of time series forecasting, as no enough evidences from historical data imply the change. As will be shown in Fig 9, the forecasting model can well capture the slowdown once it is given more information.

Four rows of plots indicate the related results of the ARIMA, ETS, VAR and NNETTS forecasting models from top to bottom. Three groups of columns, combined with the plots of both the estimated population age proportions and corresponding residuals, coincide with the young, middle-aged and old populations from left to right, where five residual plots indicate the LCC.Y, LCC.M, LCC.Y, ILR and DRHT transformation methods from top to bottom.

For India and Vietnam, as shown in Figs 7 and 8, most models were able to obtain reasonable results. Similar to that found in Fig 6, some models performed a little bad in detecting the change in the test set.

Detailed information refers to S73–S144 Figs.

Detailed information refers to S145–S216 Figs.

Next, we examined whether the residual series is white noise significantly through both the Box-Pierce [74] and Ljung-Box [81] tests. Table 6 reports the p-values of these two types of test statistics for all the models. Specifically, if the p-value is larger than the given significance level (say 0.05), we can conclude that the corresponding residual series is white noise significantly; vice versa. As shown in Table 6, most residual series for China and Vietnam can be regarded as white noise, while most for India cannot. The result implies that there exists information possible to be further utilized from most models for India, however, the volume of this part of information would be limited due to the low CoDa-RMSE and CoDa-MAPE values for corresponding models. We conclude from these results that the alternative models, including the best-performing ones, are reliable in general.

**Table 6:** White noise tests for error series of three age periods for China, India and Vietnam.

Period	Method	ARIMA		ETS		VAR		NNETTS	
		BP	LB	BP	LB	BP	LB	BP	LB
China									
Young	LCC.Y	0.438	0.309	0.305	0.194	>0.999	>0.999	0.004	0.002
	LCC.M	0.461	0.251	0.407	0.279	>0.999	>0.999	0.002	0.001
	LCC.O	0.461	0.251	0.407	0.279	>0.999	>0.999	0.002	0.001
	ILR	0.633	0.427	0.401	0.274	0.999	0.999	0.002	0.001
	DRHT	0.372	0.246	0.169	0.096	>0.999	>0.999	0.003	0.001
Middle	LCC.Y	0.705	0.588	0.503	0.372	>0.999	>0.999	0.003	0.002
	LCC.M	0.655	0.455	0.643	0.517	>0.999	>0.999	0.002	0.001
	LCC.O	0.705	0.588	0.503	0.372	>0.999	>0.999	0.003	0.002
	ILR	0.802	0.646	0.637	0.509	>0.999	>0.999	0.003	0.002
	DRHT	0.595	0.463	0.362	0.248	>0.999	>0.999	0.003	0.002
Old	LCC.Y	0.623	0.511	0.597	0.482	>0.999	>0.999	0.006	0.003
	LCC.M	0.623	0.511	0.597	0.482	>0.999	>0.999	0.006	0.003
	LCC.O	0.208	0.114	0.968	0.95	>0.999	>0.999	0.003	0.001
	ILR	0.697	0.576	0.605	0.488	>0.999	>0.999	0.003	0.002
	DRHT	0.348	0.224	0.619	0.505	>0.999	>0.999	0.003	0.002
India									
Young	LCC.Y	0.122	0.044	0.006	0.001	>0.999	>0.999	0.003	0.002
	LCC.M	0.035	0.007	0.009	0.001	>0.999	>0.999	0.003	0.001
	LCC.O	0.035	0.007	0.009	0.001	>0.999	>0.999	0.003	0.001
	ILR	0.137	0.049	0.005	0.001	>0.999	>0.999	0.003	0.001
	DRHT	0.198	0.085	0.012	0.002	>0.999	>0.999	0.003	0.001
Middle	LCC.Y	0.043	0.011	<0.001	<0.001	>0.999	>0.999	0.003	0.002
	LCC.M	0.047	0.009	<0.001	<0.001	>0.999	>0.999	0.003	0.001
	LCC.O	0.043	0.011	<0.001	<0.001	>0.999	>0.999	0.003	0.002
	ILR	0.055	0.015	<0.001	<0.001	>0.999	>0.999	0.003	0.002
	DRHT	0.088	0.028	<0.001	<0.001	>0.999	>0.999	0.004	0.002
Old	LCC.Y	0.05	0.023	<0.001	<0.001	>0.999	>0.999	0.003	0.002
	LCC.M	0.05	0.023	<0.001	<0.001	>0.999	>0.999	0.003	0.002
	LCC.O	0.546	0.383	<0.001	<0.001	>0.999	>0.999	0.003	0.002
	ILR	0.091	0.045	<0.001	<0.001	>0.999	>0.999	0.003	0.002
	DRHT	0.832	0.739	<0.001	<0.001	>0.999	>0.999	0.003	0.002
Vietnam									
Young	LCC.Y	0.89	0.817	0.048	0.021	>0.999	>0.999	0.295	0.246
	LCC.M	0.977	0.957	0.102	0.046	>0.999	>0.999	0.266	0.219
	LCC.O	0.977	0.957	0.102	0.046	>0.999	>0.999	0.266	0.219
	ILR	0.986	0.974	0.053	0.023	>0.999	>0.999	0.279	0.231
	DRHT	0.99	0.98	0.048	0.021	>0.999	>0.999	0.27	0.222
Middle	LCC.Y	0.999	0.998	0.1	0.05	>0.999	>0.999	0.292	0.244
	LCC.M	0.998	0.996	0.236	0.132	>0.999	>0.999	0.273	0.225
	LCC.O	0.999	0.998	0.1	0.05	>0.999	>0.999	0.292	0.244
	ILR	0.999	0.999	0.155	0.086	>0.999	>0.999	0.294	0.245
	DRHT	>0.999	>0.999	0.093	0.045	>0.999	>0.999	0.3	0.25
Old	LCC.Y	0.91	0.858	0.001	<0.001	>0.999	>0.999	0.307	0.257
	LCC.M	0.91	0.858	0.001	<0.001	>0.999	>0.999	0.307	0.257
	LCC.O	0.228	0.131	0.004	0.001	>0.999	>0.999	0.281	0.233
	ILR	0.885	0.819	0.015	0.005	>0.999	>0.999	0.285	0.237
	DRHT	0.912	0.861	0.001	<0.001	>0.999	>0.999	0.287	0.23

The sub-columns “BP” and “LB” denote the p-values of Box-Pierce or Ljung-Box tests, respectively.

Finally, the model with the lowest CoDa-RMSE and CoDa-MAPE values is recognized as the best-performing forecasting model with the lowest prediction error. In Table 5, the forecasting models ILR-ARIMA, ILR-ETS, and ILR-ETS achieve the lowest values of CoDa-RMSE and CoDa-MAPE in China, India, and Vietnam, respectively, with 0.06 and 3.94%, 0.01 and 0.81%, and 0.07 and 4.14%. Therefore, this study selects these forecasting models as the best-performing forecasting model for the population structure predictions of China, India, and Vietnam from 2017 to 2035, respectively. The forecasting results for the three countries are presented in Table 7.

**Table 7:** Forecasting Results of the population structures for China, India, and Vietnam (Unit: %).

Year	China			India			Vietnam		
	Young	Middle	Old	Young	Middle	Old	Young	Middle	Old
2017	17.69	71.72	10.58	27.74	66.29	5.97	23.06	69.84	7.1
2018	17.66	71.29	11.06	27.29	66.57	6.14	23.04	69.68	7.29
2019	17.6	70.85	11.55	26.84	66.85	6.31	23.01	69.51	7.48
2020	17.54	70.4	12.06	26.4	67.12	6.48	22.98	69.35	7.67
2021	17.46	69.96	12.58	25.96	67.38	6.66	22.95	69.18	7.87
2022	17.37	69.51	13.12	25.52	67.63	6.84	22.92	69.01	8.07
2023	17.28	69.04	13.68	25.09	67.88	7.03	22.88	68.84	8.28
2024	17.19	68.55	14.26	24.66	68.12	7.22	22.85	68.67	8.49
2025	17.09	68.05	14.86	24.24	68.35	7.41	22.81	68.49	8.7
2026	16.99	67.52	15.49	23.82	68.57	7.61	22.76	68.31	8.92
2027	16.9	66.97	16.14	23.41	68.78	7.82	22.72	68.13	9.15
2028	16.79	66.4	16.8	23	68.98	8.02	22.67	67.95	9.38
2029	16.69	65.82	17.5	22.59	69.18	8.24	22.62	67.76	9.61
2030	16.58	65.21	18.21	22.19	69.36	8.45	22.57	67.57	9.85
2031	16.47	64.59	18.95	21.79	69.54	8.67	22.52	67.38	10.1
2032	16.34	63.95	19.7	21.39	69.71	8.9	22.47	67.18	10.35
2033	16.22	63.3	20.49	21	69.87	9.13	22.41	66.98	10.61
2034	16.08	62.63	21.29	20.62	70.02	9.36	22.35	66.78	10.87

Detailed information refers to S217–S225 Figs.

**Fig 10:**
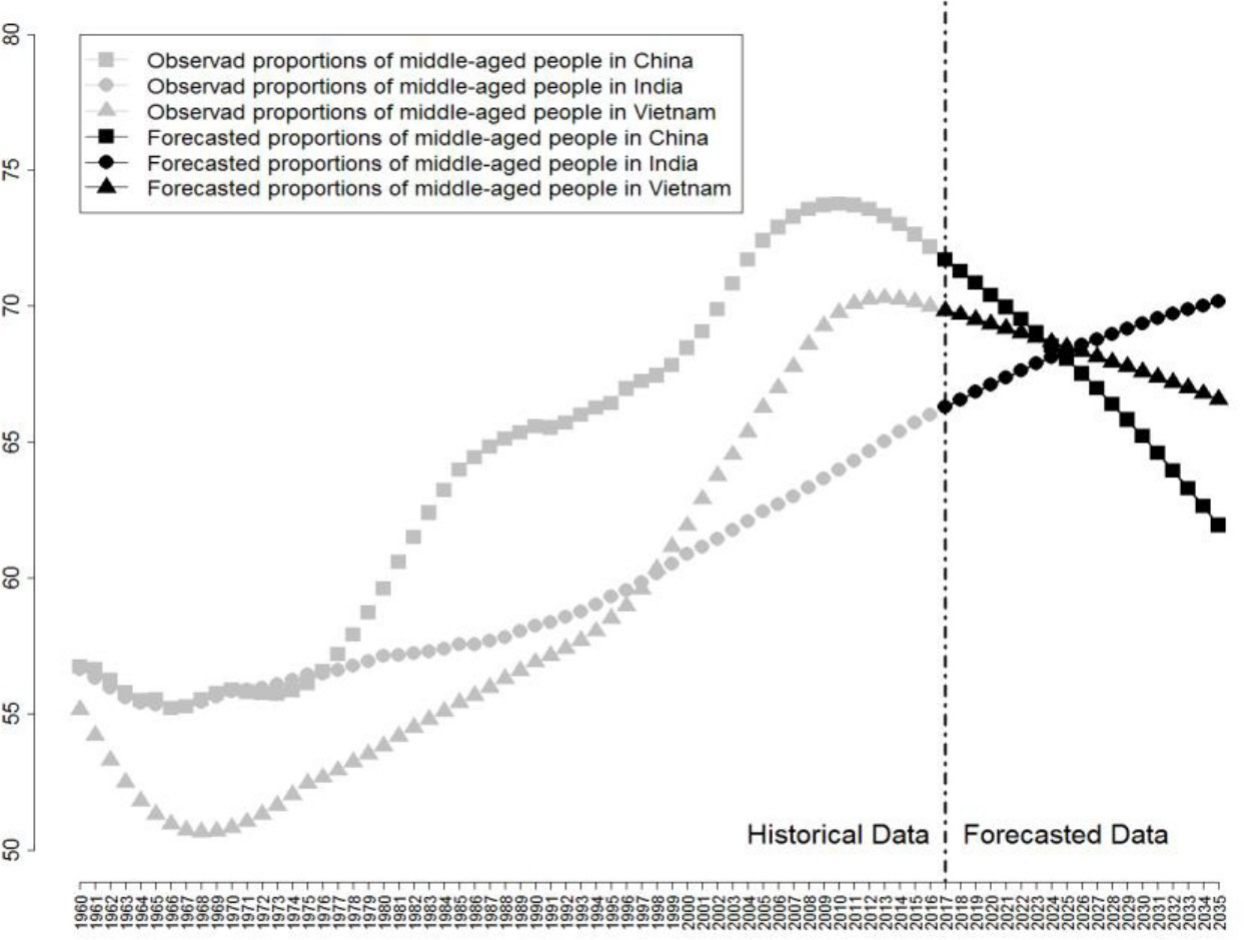
Proportions of middle-aged people (aged 15–64) and Future Trend of China, India, and Vietnam from 1960 to 2030.

**Fig 11:**
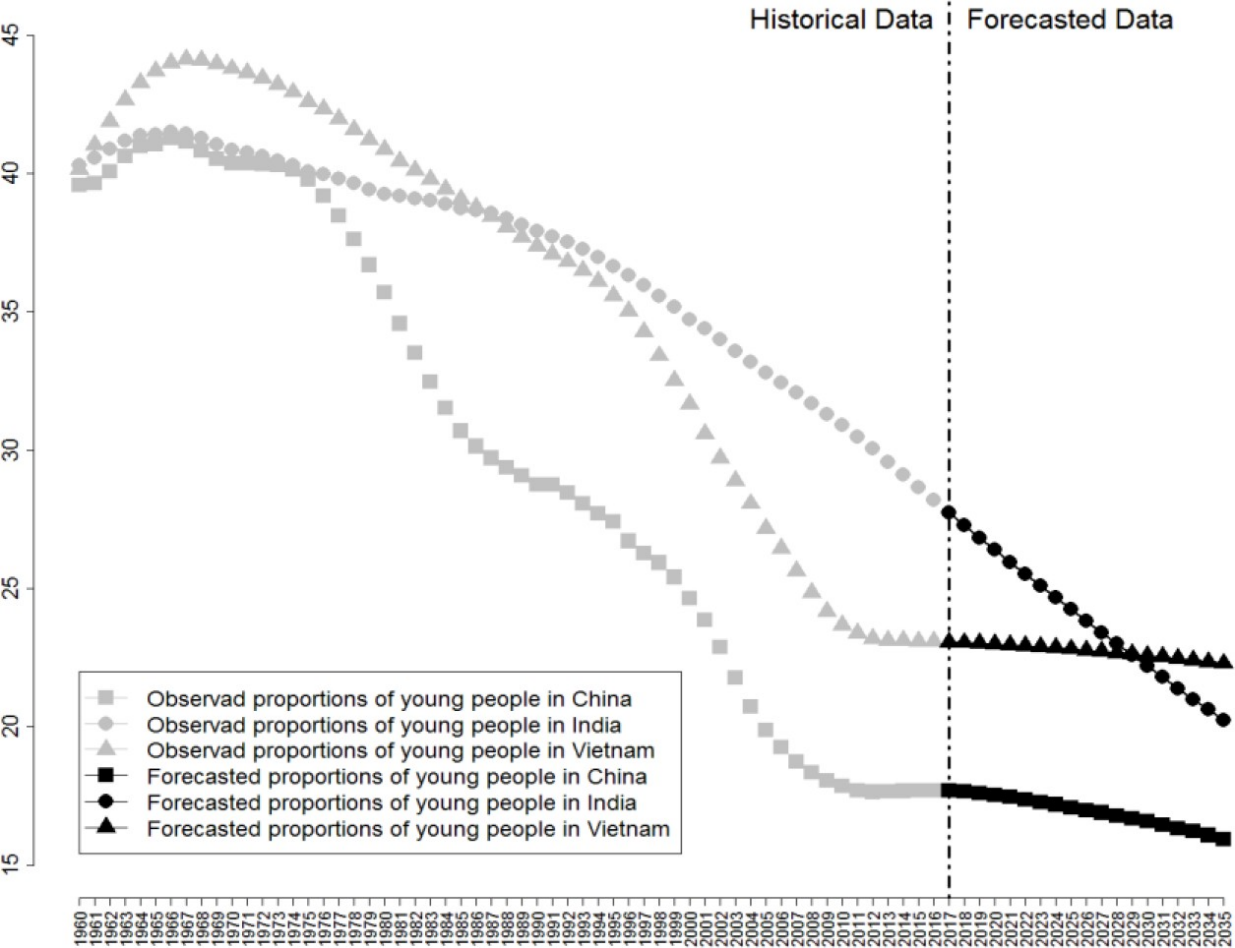
Proportion of Young People (aged 0–14) and Future Trend of China, India, and Vietnam from 1960 to 2030.

**Fig 12:**
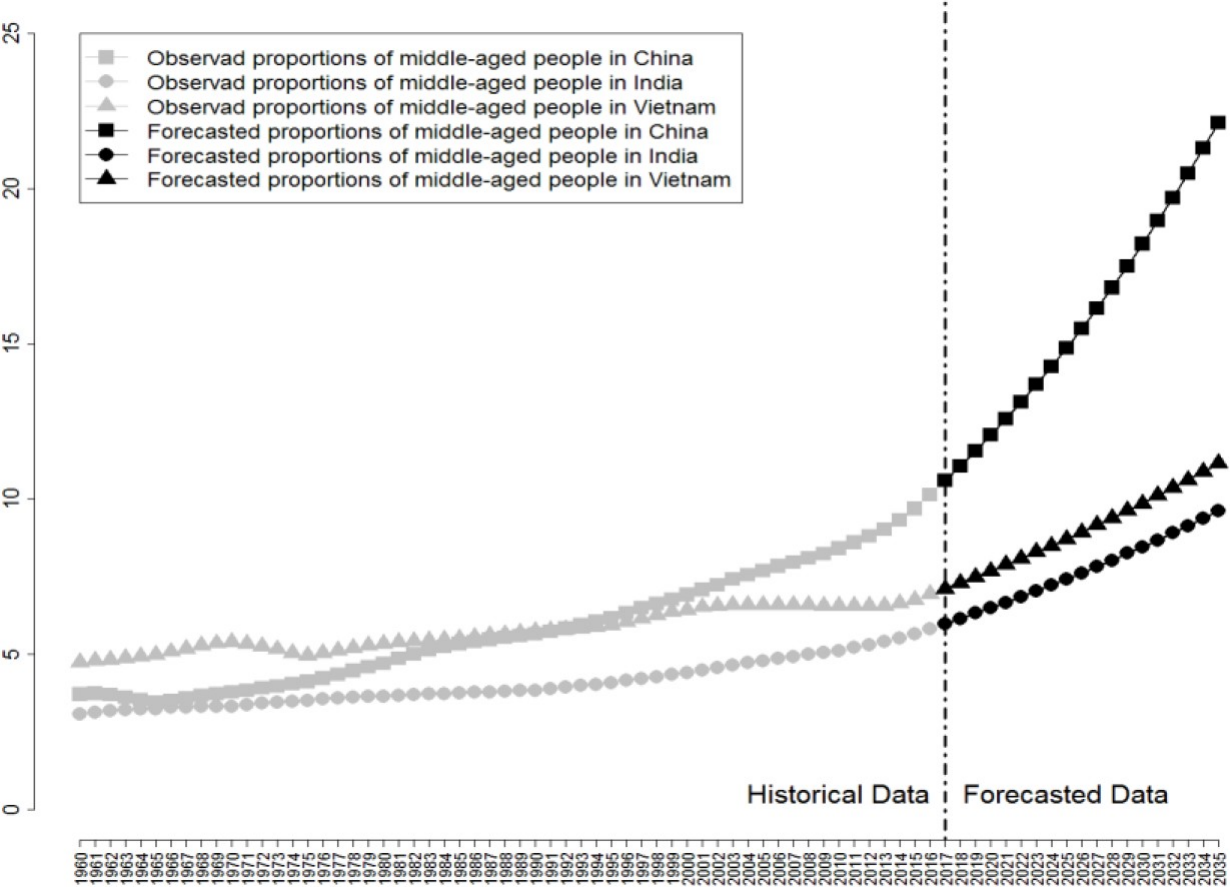
Proportion of Old People (aged older than 65) and Future Trend of China, India, and Vietnam from 1960 to 2030.

Figs 10–12 reveal respectively the proportion and future development trend of middle-aged people (aged 15–64), young people (aged 0–14), and old people (aged older than 65) from China, India, and Vietnam from 1960 to 2030.

As revealed by Fig 10, the population structures of the three countries from 1960 to 2030 appear to follow different trends. In China, during the early stage of reform and opening up, the proportion of middle-aged people in population remained above 60%; it continued to rise and surpassed 70% in 2003. The year 2010 was a critical turning point in China’s population structure. Ever since the proportion of middle-aged population peaked at 73.75%, it started to decrease year by year, and is estimated to experience a drastic reduction to approximately 63.44% in 2030, approximately 10% lower than that in 2010. The trend in the change of Vietnam’s population is similar to that of China, except that the proportion of its middle-aged people will not fall as steeply as that of China. The proportion of middle-aged people in Vietnam’s population is estimated to fall from the 70.30% peak in 2013 to 67.46% in 2030, by less than 3%, which is a comparatively more moderate decrease. Clearly different from China and Vietnam, the proportion of middle-aged people in India is expected to sustain its increase and reach 70.48% in 2030, which is a sufficient indicator that the middle-aged population will continue to play a crucial role in India’s population, and that the middle-aged people-oriented population structure will remain stable for the time being. From the above analysis, despite differences in the proportion of middle-aged people in China, India, and Vietnam, there is no denying that middle-aged people are playing an equally crucial role in the economic development of the three countries.

The reasons for the great discrepancies between the proportions of middle-aged people in the three countries are the following. Although China’s population has already completed its transition from traditional stage to the modern stage, its proportion of labor force is nevertheless in gradual decline, exhausting the demographic dividend. Not only rooted in the birth control policy, such current situations are also attributed to the decrease in the desire for children among the Chinese, caused by high cost of child-rearing, difficulties in child-care service, as well as economic growth, high urbanization migration rate, and popularization of higher education. Since 1993, Vietnam has strictly implemented the population and family planning policy, which proved to be a sufficient tool for tackling the massive size and high birth rate of its population. As for India, its proportion of middle-aged people continues to rise, vitalizing its labor force supply. The Indian government released its population policy in the year 2000, in which it claimed that the population size of India will stabilize at around 1.45 billion in 2045. However, concerning the estimated result, it would be an enormous difficulty if not impossible for India to achieve this goal. Yet, on the other hand, despite the tension caused by its yearly 0.4% increase in the proportion of middle-aged population, India’s population will nonetheless remain in a young and vital stage, which is the fuel for India to rise in Asia [82].

Fig 11 reveals the proportion of young people (aged 0–14) and its future trend in China, India, and Vietnam from 1960 to 2030. As observed in Fig 11, the proportions of young people in the three countries appear to be declining. The changes in China and Vietnam are similar, with both the countries registering an increase but reaching a turning point around 1967, after which the proportion of young people in the population started to decline. Until around 2011, the proportions of young people of the two countries ceased to decline and gradually leveled off. As estimated, the proportion of young people in China’s population will remain at 17.7% during 2011–2030, whereas it will be 23.0% in Vietnam. In accordance with the global trend, recently, the proportion of young people in India’s population has plunged from 40.30% in 1960 to 28.66% in 2015; it is estimated to shrink to 21.20% in 2030 and maintain a downward trend. As shown is Fig 11, all three countries, to varying degrees, are going through a downward movement with regard to proportion of young people in the population, which also directly reflects the influence of population control measures worldwide.

The proportion of China’s young people in the population has gone through three stages: it first remained relatively high during the 1970s, then declined drastically during the 1990s, and finally leveled off and remained relatively low starting from 2010. According to the estimation, the absolute quantity and the proportion of young people will remain stable if not slightly shrink, which further indicates that the bottom of China’s population pyramid will gradually shrink, having a huge impact on China’s labor market, depleting the labor cost advantage and suppressing China’s international competitiveness. In Vietnam, the changing proportion of young people in the population resembles China; it also leveled off after a drastic decline and is estimated to remain stable until 2030. India is witnessing the fastest population growth, at a speed much higher than that of China. Despite the fact that the low-price Indian labor market and its endless supply of labor force appear to be an irresistible attraction to multinational corporations from developed countries, India is attempting to upgrade its labor market structure, gear up corporation development vitality, and strengthen its comparative advantages in demographic dividend by providing higher-quality education for young and middle-aged people. Above all, due to the gradual leveling off in the proportion of young people in the population, offering better education for young and middle-aged people and in the meantime ensuring their basic living needs has become a global challenge.

Fig 12 reveals the proportion of elder people (aged older than 65) and its future trend in China, India, and Vietnam from 1960 to 2030. It can be observed that the proportions of old people in the three countries are all in continuous increase, with that of China being the fastest. By estimation, the proportion of old people in China’s population will reach 19.25% in 2030, far outnumbering Vietnam’s at 10.18% and India’s at 8.03%, which indicates that China will become an overall aging society, featuring continuously accelerating aging tendency, gradual loss of labor cost advantage, and decline in demographic dividend. Obviously, countries around the globe are more or less faced with the aging problem—how to tackle problems caused by the ever increasing proportion of old people has become a pressing concern worldwide.

The aging population, albeit a global challenge, is much tougher to handle in China than elsewhere. Apart from its massive size and high aging speed, China’s aging population is also characterized by deep aging degree and fragile health condition. In comparison, Vietnam’s old-age dependency ratio has already peaked at 8% around the 1960s, marking its early entrance into aging society. As the old-age dependency is rising continuously across the globe, Vietnam’s population, however, has seen a downward trend in this ratio, which indicates a rare opportunity for Vietnam’s development. India is the youngest of the three countries, with the lowest proportion of old people, offering it with sufficient demographic dividend and market potential, making it stand out on the background of global aging. In addition, the total birth rate of India in 2015 still remained at around 2%, which indicated that its demographic dividend could last for ten years or even longer. In order to fully utilize its demographic dividend, the Indian government has been playing an active role. In 2015, the child dependency ratio of India was 44%, well above China’s 24%; in the same year, the old-age dependency ratio of India was 8%, higher than China’s 13%. Although the current total dependency ratio of China is 37%, well below India’s 52%, as seen from the predicted data, this ratio in China is witnessing a speedy increase, as it is predicted to rise from the 13% in 2015 to 29% in 2030, while in India, it will only rise from 8% to 11% during the same period. As a result, up to 2030, the total dependency ratio of China will reach 55%, far above India’s 40%, which could be the heaviest burden ever for China’s population, posing tremendous challenges to China’s development.

As mentioned before, it is the old-age dependency ratio that dominates China’s total dependency ratio growth, whereas for India, the child dependency ratio dominates. China has already entered a high-speed channel of population aging, yet India’s population still remains at a relatively young stage. The gradual decrease and disappearance of China’s demographic dividend will result in an increase in the proportion of old people in the population as well as decrease in the proportion of labor force. China is fast becoming an aging society. The home-based care system for old people will sooner or later be overwhelming and superseded by an overall social welfare system yet to be completed, which is a higher requirement as well as expectation for China’s economy, politics, culture, and numerous other aspects of society.

## Conclusion and policy recommendation

To explore the development of population structure and predict the changes in population size are of crucial importance for socioeconomic policy enactment. In recent years, China and the Southeast Asian countries will enter or have already entered the aging society. The increase of old people inevitably turns demographic dividend into a demographic burden. It is of urgent importance to not only China but every country around the world to fully utilize the potential contribution of population transformation. According to historical data, the present study has conducted quantitative analysis on the development pattern and future trend of the population structures of China, India, and Vietnam, and predicted the development trends of different age groups till the year 2030 for the three countries. In an attempt to propose strategic and practical suggestions to avoid the aging problem for planners of the global development blueprint, the findings of present study can be meaningful for the formulation and implementation of future demographic policies.

The proposed forecasting models using compositional data, compared with the conventional time series approaches, performed better in forecasting trends of the population age structures for China, India and Vietnam. The data transformation methods for compositional data and forecasting models are two key steps for forecasting using compositional data. First, to eliminate the nonnegative and constant-sum constraints of compositional data, three data transformation methods, namely the LCC, ILR and DRHT methods, were introduced. The pros and cons of each transformations methods are compared as follows.

1. The logics and implementation of LCC methods are straightforward and ease of using. However, as shown in Table 5, this method may result in a large and uncertain bias when it is directly combined with some complex forecasting approaches. For example, the CoDa-MAPE value of LCC.O reaches 37.12% for Vietnam, which is far more than that of LCC.Y (3.43%). The subjective choice of the combined component in LCC methods also causes extra issues to be discussed.
2. The ILR method performed relatively well in general; specifically, the best-performed models for three countries all involve the ILR method. As a mature technique in compositional data analysis, the ILR method proves effective methods to the population age structure prediction. However, the downsides of this method are its complex formulas in computation and the failure to dealing with zero.
3. The DRHT method allows the existence of part of zero, which also can realizes reasonable results when combined with most of forecasting methods. However, its performance was slightly lower than the ILR method in terms of prediction errors.

Second, four popular forecasting methods, namely ARIMA, ETS, VAR and NNETTS, were conducted to forecast the population age structures for three countries by 2035. The pros and cons of each forecasting methods are compared as follows:

1. The ARIMA model is generally reliable; specifically in Table 5, it was selected as the best-performed model for China, combined with the ILR method. However, as determined by a large amount of historical data, this method was not sensitive to the recent changes of time series; its performance was sometimes slightly worse than other short-term forecasting approaches such as ETS.
2. The data requirement of the ETS model is more flexible; therefore, it was selected as the best-performed model for both India and Vietnam, combined with the ILR method.
3. As a multivariate time series forecasting approach, the VAR model can captures the linear correlation among different periods of the population. However, the requirement of a large sample size reduced its performance in the test set that was ordered behind the ILR and ETS models.
4. The family of NNETTS models performed a little bad in the population age structure prediction in this study. As an advanced non-linear forecasting approach, this method involves some tuning parameters to be determined empirically, which increased the difficulty of modeling. As shown in Table 4, under different settings of these parameters, the results varied dramatically (e.g., the CoDa-MAPE values from 3.94% to 30.93% for China), when they are combined data transformations for compositional data.

According to the prediction results, several important findings are revealed.

1. By the year 2030, the proportion of middle-aged people (aged 15–64) will make up, respectively, 63.44%, 70.77%, and 67.26% of the populations of China, India, and Vietnam. Among the three countries, China is of the least advantage in terms of labor force population. The proportions of young people (aged 0–14) are 17.30%, 21.20%, and 22.56%, respectively, among which those of China and Vietnam are gradually leveling off, whereas that of India is still in continuous decline. The proportions of old people in the three countries are 19.25%, 8.03%, and 10.18%, respectively.
2. The aging degree of China’s population is far more rapid then that of Vietnam or India. Moreover, the proportion of middle-aged people in China’s population will see a continuous downtrend in the near future, and decreased rapidly from the 73.75% of 2010 to 63.44% in 2030, with an above 10% fall, while the proportion of young people will remain stable and without much fluctuation.
3. The proportions of middle-aged and young people in Vietnam’s population will experience similar future trends compared to China, despite the relatively more moderate decrease in the proportion of middle-aged people. Vietnam’s proportion of young people will remain in a healthy stage, while the proportion of old people will see a slight increase.
4. As for the future of India’s population structure, the proportion of labor force will rise continuously and rapidly. Meanwhile, its proportion of young people will continue to fall, and is estimated to sink to 21.20% by 2030. The proportion of old people will be in slow increase, and is estimated to reach 8.03% by 2030, the lowest of the three countries regarding old people’s proportion.

Based on the results of population projection, the present study attempts to propose suggestions in accordance with the economic, political, and cultural situations of the three countries, and, to an extent, provide inspirations for other countries.

1. For China, the ever-intensifying aging problem, disappearance of demographic dividend, and the continuously shrinking labor force resources has become an undeniable truth. However, China’s current massive-sized and high-quality labor force appear to be the greatest advantage in international competition, as well as a major fuel of China’s economic development. Therefore, what China should do is to increase the investment of human capital, improve the competitiveness of labor force, fully utilize the residual demographic dividend, and sustain current competitiveness of China’s manufacturing industry. In addition, due to the huge discrepancies between China’s rural and urban population, it is also suggested to balance and integrate the labor markets, both urban and rural, and to facilitate the development of labor force market. Meanwhile, it is also crucial to accelerate the upgradation of industrial structure and the transformation of economic growth. In order to achieve sustainable economic development, China should also increase the investment of technology to advance technological development. Overall, it is urgent for China to change its previous extensive way of labor force utilization, increase investment in human capital, conduct secondary labor force development, and further, strengthen industrial competitiveness in an aging society and promote comprehensive social development.
2. For India, given its massive population size and young population age structure, it is suggested to improve the overall quality of population and increase the stock of human capital. The demographic economic development of India should focus on the improvement of population quality, rather than over-relying on birth control policy to maintain the so-called “economically beneficial population age structure”. For instance, considering the huge gap between urban and rural areas and different regions, the Indian government should provide certain political preference for areas with low human capital stock, in order to improve the overall stock of human capital speedily and economically. It is also a plausible solution to speed up the popularization of compulsory education.
3. Vietnam, with a relatively comprehensive population age structure, is currently still enjoying its demographic dividend. What the Vietnamese government should do is to complete its caring system for senior citizens, in order to cope actively with the aging tendency. Facilitating public services can relieve the aging problem to a certain extent, stimulate domestic demand in the short run, ensure the supply of labor force in the long run, and meanwhile offer a way out of the decline of home-based caring resources. Specifically speaking, the establishment of a wide-ranging, multi-level old-age caring system can prove useful for easing off the caring burden of the labor force within families, as well as satisfying the basic needs of old people both materially and spiritually.

## Supporting information

S1 Fig Estimated-China-1-ARIMA. (PNG)

S2 Fig Estimated-China-1-ETS. (PNG)

S3 Fig Estimated-China-1-NNETTS. (PNG)

S4 Fig Estimated-China-1-VAR. (PNG)

S5 Fig Estimated-China-2-ARIMA. (PNG)

S6 Fig Estimated-China-2-ETS. (PNG)

S7 Fig Estimated-China-2-NNETTS. (PNG)

S8 Fig Estimated-China-2-VAR. (PNG)

S9 Fig Estimated-China-3-ARIMA. (PNG)

S10 Fig Estimated-China-3-ETS. (PNG)

S11 Fig Estimated-China-3-NNETTS. (PNG)

S12 Fig Estimated-China-3-VAR. (PNG)

S13 Fig Residuals-China-1-ARIMA-DRHT. (PNG)

S14 Fig Residuals-China-1-ARIMA-ILR. (PNG)

S15 Fig Residuals-China-1-ARIMA-LCCM. (PNG)

S16 Fig Residuals-China-1-ARIMA-LCCO. (PNG)

S17 Fig Residuals-China-1-ARIMA-LCCY. (PNG)

S18 Fig Residuals-China-1-ETS-DRHT. (PNG)

S19 Fig Residuals-China-1-ETS-ILR. (PNG)

S20 Fig Residuals-China-1-ETS-LCCM. (PNG)

S21 Fig Residuals-China-1-ETS-LCCO. (PNG)

S22 Fig Residuals-China-1-ETS-LCCY. (PNG)

S23 Fig Residuals-China-1-NNETTS-DRHT. (PNG)

S24 Fig Residuals-China-1-NNETTS-ILR. (PNG)

S25 Fig Residuals-China-1-NNETTS-LCCM. (PNG)

S26 Fig Residuals-China-1-NNETTS-LCCO. (PNG)

S27 Fig Residuals-China-1-NNETTS-LCCY. (PNG)

S28 Fig Residuals-China-1-VAR-DRHT. (PNG)

S29 Fig Residuals-China-1-VAR-ILR. (PNG)

S30 Fig Residuals-China-1-VAR-LCCM. (PNG)

S31 Fig Residuals-China-1-VAR-LCCO. (PNG)

S32 Fig Residuals-China-1-VAR-LCCY. (PNG)

S33 Fig Residuals-China-2-ARIMA-DRHT. (PNG)

S34 Fig Residuals-China-2-ARIMA-ILR. (PNG)

S35 Fig Residuals-China-2-ARIMA-LCCM. (PNG)

S36 Fig Residuals-China-2-ARIMA-LCCO. (PNG)

S37 Fig Residuals-China-2-ARIMA-LCCY. (PNG)

S38 Fig Residuals-China-2-ETS-DRHT. (PNG)

S39 Fig Residuals-China-2-ETS-ILR. (PNG)

S40 Fig Residuals-China-2-ETS-LCCM. (PNG)

S41 Fig Residuals-China-2-ETS-LCCO. (PNG)

S42 Fig Residuals-China-2-ETS-LCCY. (PNG)

S43 Fig Residuals-China-2-NNETTS-DRHT. (PNG)

S44 Fig Residuals-China-2-NNETTS-ILR. (PNG)

S45 Fig Residuals-China-2-NNETTS-LCCM. (PNG)

S46 Fig Residuals-China-2-NNETTS-LCCO. (PNG)

S47 Fig Residuals-China-2-NNETTS-LCCY. (PNG)

S48 Fig Residuals-China-2-VAR-DRHT. (PNG)

S49 Fig Residuals-China-2-VAR-ILR. (PNG)

S50 Fig Residuals-China-2-VAR-LCCM. (PNG)

S51 Fig Residuals-China-2-VAR-LCCO. (PNG)

S52 Fig Residuals-China-2-VAR-LCCY. (PNG)

S53 Fig Residuals-China-3-ARIMA-DRHT. (PNG)

S54 Fig Residuals-China-3-ARIMA-ILR. (PNG)

S55 Fig Residuals-China-3-ARIMA-LCCM. (PNG)

S56 Fig Residuals-China-3-ARIMA-LCCO. (PNG)

S57 Fig Residuals-China-3-ARIMA-LCCY. (PNG)

S58 Fig Residuals-China-3-ETS-DRHT. (PNG)

S59 Fig Residuals-China-3-ETS-ILR. (PNG)

S60 Fig Residuals-China-3-ETS-LCCM. (PNG)

S61 Fig Residuals-China-3-ETS-LCCO. (PNG)

S62 Fig Residuals-China-3-ETS-LCCY. (PNG)

S63 Fig Residuals-China-3-NNETTS-DRHT. (PNG)

S64 Fig Residuals-China-3-NNETTS-ILR. (PNG)

S65 Fig Residuals-China-3-NNETTS-LCCM. (PNG)

S66 Fig Residuals-China-3-NNETTS-LCCO. (PNG)

S67 Fig Residuals-China-3-NNETTS-LCCY. (PNG)

S68 Fig Residuals-China-3-VAR-DRHT. (PNG)

S69 Fig Residuals-China-3-VAR-ILR. (PNG)

S70 Fig Residuals-China-3-VAR-LCCM. (PNG)

S71 Fig Residuals-China-3-VAR-LCCO. (PNG)

S72 Fig Residuals-China-3-VAR-LCCY. (PNG)

S73 Fig Estimated-India-1-ARIMA. (PNG)

S74 Fig Estimated-India-1-ETS. (PNG)

S75 Fig Estimated-India-1-NNETTS. (PNG)

S76 Fig Estimated-India-1-VAR. (PNG)

S77 Fig Estimated-India-2-ARIMA. (PNG)

S78 Fig Estimated-India-2-ETS. (PNG)

S79 Fig Estimated-India-2-NNETTS. (PNG)

S80 Fig Estimated-India-2-VAR. (PNG)

S81 Fig Estimated-India-3-ARIMA. (PNG)

S82 Fig Estimated-India-3-ETS. (PNG)

S83 Fig Estimated-India-3-NNETTS. (PNG)

S84 Fig Estimated-India-3-VAR. (PNG)

S85 Fig Residuals-India-1-ARIMA-DRHT. (PNG)

S86 Fig Residuals-India-1-ARIMA-ILR. (PNG)

S87 Fig Residuals-India-1-ARIMA-LCCM. (PNG)

S88 Fig Residuals-India-1-ARIMA-LCCO. (PNG)

S89 Fig Residuals-India-1-ARIMA-LCCY. (PNG)

S90 Fig Residuals-India-1-ETS-DRHT. (PNG)

S91 Fig Residuals-India-1-ETS-ILR. (PNG)

S92 Fig Residuals-India-1-ETS-LCCM. (PNG)

S93 Fig Residuals-India-1-ETS-LCCO. (PNG)

S94 Fig Residuals-India-1-ETS-LCCY. (PNG)

S95 Fig Residuals-India-1-NNETTS-DRHT. (PNG)

S96 Fig Residuals-India-1-NNETTS-ILR. (PNG)

S97 Fig Residuals-India-1-NNETTS-LCCM. (PNG)

S98 Fig Residuals-India-1-NNETTS-LCCO. (PNG)

S99 Fig Residuals-India-1-NNETTS-LCCY. (PNG)

S100 Fig Residuals-India-1-VAR-DRHT. (PNG)

S101 Fig Residuals-India-1-VAR-ILR. (PNG)

S102 Fig Residuals-India-1-VAR-LCCM. (PNG)

S103 Fig Residuals-India-1-VAR-LCCO. (PNG)

S104 Fig Residuals-India-1-VAR-LCCY. (PNG)

S105 Fig Residuals-India-2-ARIMA-DRHT. (PNG)

S106 Fig Residuals-India-2-ARIMA-ILR. (PNG)

S107 Fig Residuals-India-2-ARIMA-LCCM. (PNG)

S108 Fig Residuals-India-2-ARIMA-LCCO. (PNG)

S109 Fig Residuals-India-2-ARIMA-LCCY. (PNG)

S110 Fig Residuals-India-2-ETS-DRHT. (PNG)

S111 Fig Residuals-India-2-ETS-ILR. (PNG)

S112 Fig Residuals-India-2-ETS-LCCM. (PNG)

S113 Fig Residuals-India-2-ETS-LCCO. (PNG)

S114 Fig Residuals-India-2-ETS-LCCY. (PNG)

S115 Fig Residuals-India-2-NNETTS-DRHT. (PNG)

S116 Fig Residuals-India-2-NNETTS-ILR. (PNG)

S117 Fig Residuals-India-2-NNETTS-LCCM. (PNG)

S118 Fig Residuals-India-2-NNETTS-LCCO. (PNG)

S119 Fig Residuals-India-2-NNETTS-LCCY. (PNG)

S120 Fig Residuals-India-2-VAR-DRHT. (PNG)

S121 Fig Residuals-India-2-VAR-ILR. (PNG)

S122 Fig Residuals-India-2-VAR-LCCM. (PNG)

S123 Fig Residuals-India-2-VAR-LCCO. (PNG)

S124 Fig Residuals-India-2-VAR-LCCY. (PNG)

S125 Fig Residuals-India-3-ARIMA-DRHT. (PNG)

S126 Fig Residuals-India-3-ARIMA-ILR. (PNG)

S127 Fig Residuals-India-3-ARIMA-LCCM. (PNG)

S128 Fig Residuals-India-3-ARIMA-LCCO. (PNG)

S129 Fig Residuals-India-3-ARIMA-LCCY. (PNG)

S130 Fig Residuals-India-3-ETS-DRHT. (PNG)

S131 Fig Residuals-India-3-ETS-ILR. (PNG)

S132 Fig Residuals-India-3-ETS-LCCM. (PNG)

S133 Fig Residuals-India-3-ETS-LCCO. (PNG)

S134 Fig Residuals-India-3-ETS-LCCY. (PNG)

S135 Fig Residuals-India-3-NNETTS-DRHT. (PNG)

S136 Fig Residuals-India-3-NNETTS-ILR. (PNG)

S137 Fig Residuals-India-3-NNETTS-LCCM. (PNG)

S138 Fig Residuals-India-3-NNETTS-LCCO. (PNG)

S139 Fig Residuals-India-3-NNETTS-LCCY. (PNG)

S140 Fig Residuals-India-3-VAR-DRHT. (PNG)

S141 Fig Residuals-India-3-VAR-ILR. (PNG)

S142 Fig Residuals-India-3-VAR-LCCM. (PNG)

S143 Fig Residuals-India-3-VAR-LCCO. (PNG)

S144 Fig Residuals-India-3-VAR-LCCY. (PNG)

S145 Fig Estimated-Vietnam-1-ARIMA. (PNG)

S146 Fig Estimated-Vietnam-1-ETS. (PNG)

S147 Fig Estimated-Vietnam-1-NNETTS. (PNG)

S148 Fig Estimated-Vietnam-1-VAR. (PNG)

S149 Fig Estimated-Vietnam-2-ARIMA. (PNG)

S150 Fig Estimated-Vietnam-2-ETS. (PNG)

S151 Fig Estimated-Vietnam-2-NNETTS. (PNG)

S152 Fig Estimated-Vietnam-2-VAR. (PNG)

S153 Fig Estimated-Vietnam-3-ARIMA. (PNG)

S154 Fig Estimated-Vietnam-3-ETS. (PNG)

S155 Fig Estimated-Vietnam-3-NNETTS. (PNG)

S156 Fig Estimated-Vietnam-3-VAR. (PNG)

S157 Fig Residuals-Vietnam-1-ARIMA-DRHT. (PNG)

S158 Fig Residuals-Vietnam-1-ARIMA-ILR. (PNG)

S159 Fig Residuals-Vietnam-1-ARIMA-LCCM. (PNG)

S160 Fig Residuals-Vietnam-1-ARIMA-LCCO. (PNG)

S161 Fig Residuals-Vietnam-1-ARIMA-LCCY. (PNG)

S162 Fig Residuals-Vietnam-1-ETS-DRHT. (PNG)

S163 Fig Residuals-Vietnam-1-ETS-ILR. (PNG)

S164 Fig Residuals-Vietnam-1-ETS-LCCM. (PNG)

S165 Fig Residuals-Vietnam-1-ETS-LCCO. (PNG)

S166 Fig Residuals-Vietnam-1-ETS-LCCY. (PNG)

S167 Fig Residuals-Vietnam-1-NNETTS-DRHT. (PNG)

S168 Fig Residuals-Vietnam-1-NNETTS-ILR. (PNG)

S169 Fig Residuals-Vietnam-1-NNETTS-LCCM. (PNG)

S170 Fig Residuals-Vietnam-1-NNETTS-LCCO. (PNG)

S171 Fig Residuals-Vietnam-1-NNETTS-LCCY. (PNG)

S172 Fig Residuals-Vietnam-1-VAR-DRHT. (PNG)

S173 Fig Residuals-Vietnam-1-VAR-ILR. (PNG)

S174 Fig Residuals-Vietnam-1-VAR-LCCM. (PNG)

S175 Fig Residuals-Vietnam-1-VAR-LCCO. (PNG)

S176 Fig Residuals-Vietnam-1-VAR-LCCY. (PNG)

S177 Fig Residuals-Vietnam-2-ARIMA-DRHT. (PNG)

S178 Fig Residuals-Vietnam-2-ARIMA-ILR. (PNG)

S179 Fig Residuals-Vietnam-2-ARIMA-LCCM. (PNG)

S180 Fig Residuals-Vietnam-2-ARIMA-LCCO. (PNG)

S181 Fig Residuals-Vietnam-2-ARIMA-LCCY. (PNG)

S182 Fig Residuals-Vietnam-2-ETS-DRHT. (PNG)

S183 Fig Residuals-Vietnam-2-ETS-ILR. (PNG)

S184 Fig Residuals-Vietnam-2-ETS-LCCM. (PNG)

S185 Fig Residuals-Vietnam-2-ETS-LCCO. (PNG)

S186 Fig Residuals-Vietnam-2-ETS-LCCY. (PNG)

S187 Fig Residuals-Vietnam-2-NNETTS-DRHT. (PNG)

S188 Fig Residuals-Vietnam-2-NNETTS-ILR. (PNG)

S189 Fig Residuals-Vietnam-2-NNETTS-LCCM. (PNG)

S190 Fig Residuals-Vietnam-2-NNETTS-LCCO. (PNG)

S191 Fig Residuals-Vietnam-2-NNETTS-LCCY. (PNG)

S192 Fig Residuals-Vietnam-2-VAR-DRHT. (PNG)

S193 Fig Residuals-Vietnam-2-VAR-ILR. (PNG)

S194 Fig Residuals-Vietnam-2-VAR-LCCM. (PNG)

S195 Fig Residuals-Vietnam-2-VAR-LCCO. (PNG)

S196 Fig Residuals-Vietnam-2-VAR-LCCY. (PNG)

S197 Fig Residuals-Vietnam-3-ARIMA-DRHT. (PNG)

S198 Fig Residuals-Vietnam-3-ARIMA-ILR. (PNG)

S199 Fig Residuals-Vietnam-3-ARIMA-LCCM. (PNG)

S200 Fig Residuals-Vietnam-3-ARIMA-LCCO. (PNG)

S201 Fig Residuals-Vietnam-3-ARIMA-LCCY. (PNG)

S202 Fig Residuals-Vietnam-3-ETS-DRHT. (PNG)

S203 Fig Residuals-Vietnam-3-ETS-ILR. (PNG)

S204 Fig Residuals-Vietnam-3-ETS-LCCM. (PNG)

S205 Fig Residuals-Vietnam-3-ETS-LCCO. (PNG)

S206 Fig Residuals-Vietnam-3-ETS-LCCY. (PNG)

S207 Fig Residuals-Vietnam-3-NNETTS-DRHT. (PNG)

S208 Fig Residuals-Vietnam-3-NNETTS-ILR. (PNG)

S209 Fig Residuals-Vietnam-3-NNETTS-LCCM. (PNG)

S210 Fig Residuals-Vietnam-3-NNETTS-LCCO. (PNG)

S211 Fig Residuals-Vietnam-3-NNETTS-LCCY. (PNG)

S212 Fig Residuals-Vietnam-3-VAR-DRHT. (PNG)

S213 Fig Residuals-Vietnam-3-VAR-ILR. (PNG)

S214 Fig Residuals-Vietnam-3-VAR-LCCM. (PNG)

S215 Fig Residuals-Vietnam-3-VAR-LCCO. (PNG)

S216 Fig Residuals-Vietnam-3-VAR-LCCY. (PNG)

S217 Fig F orecast-China-1. (PNG)

S218 Fig Forecast-China-2. (PNG)

S219 Fig Forecast-China-3. (PNG)

S220 Fig Forecast-India-1. (PNG)

S221 Fig Forecast-India-2. (PNG)

S222 Fig Forecast-India-3. (PNG)

S223 Fig Forecast-Vietnam-1. (PNG)

S224 Fig Forecast-Vietnam-2. (PNG)

S225 Fig Forecast-Vietnam-3. (PNG)
