# Supplementary figures and images for: Predicting population age structures of China, India, and Vietnam by 2030 based on compositional data (part 1 of 2)
Source: PLoS One. 2019 Apr 11;14(4):e0212772. doi: 10.1371/journal.pone.0212772 (PMC6459537; doi:10.1371/journal.pone.0212772)

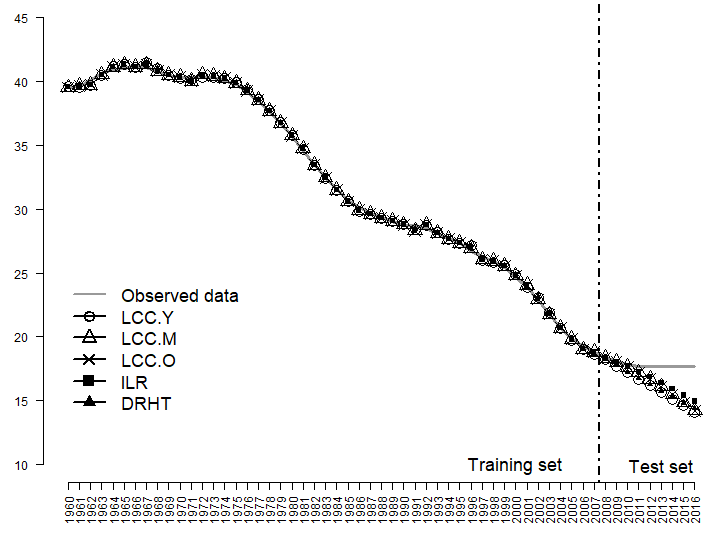

Supplement: S1 Fig — (PNG) [file pone.0212772.s001.png]

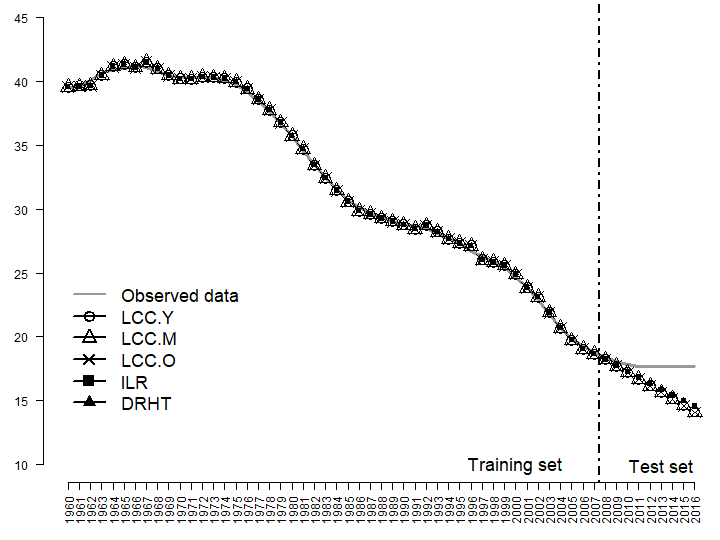

Supplement: S2 Fig — (PNG) [file pone.0212772.s002.png]

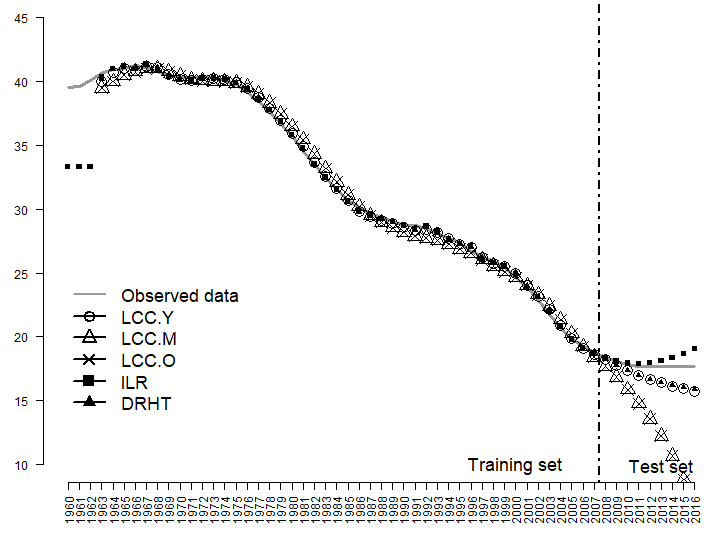

Supplement: S3 Fig — (PNG) [file pone.0212772.s003.png]

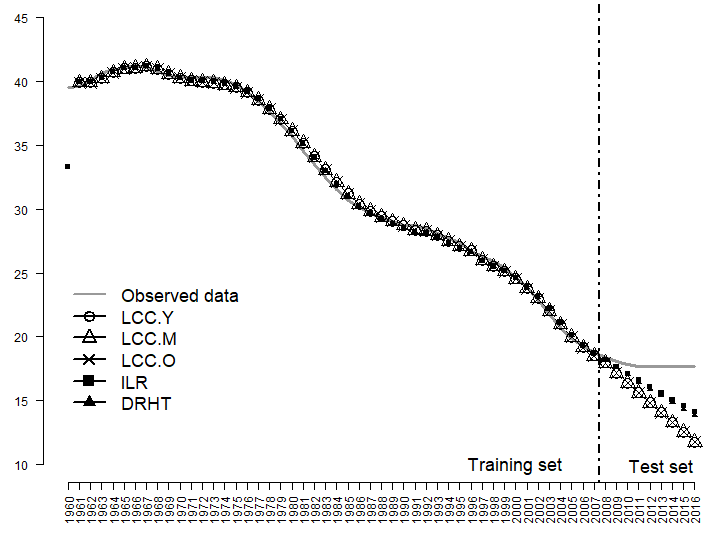

Supplement: S4 Fig — (PNG) [file pone.0212772.s004.png]

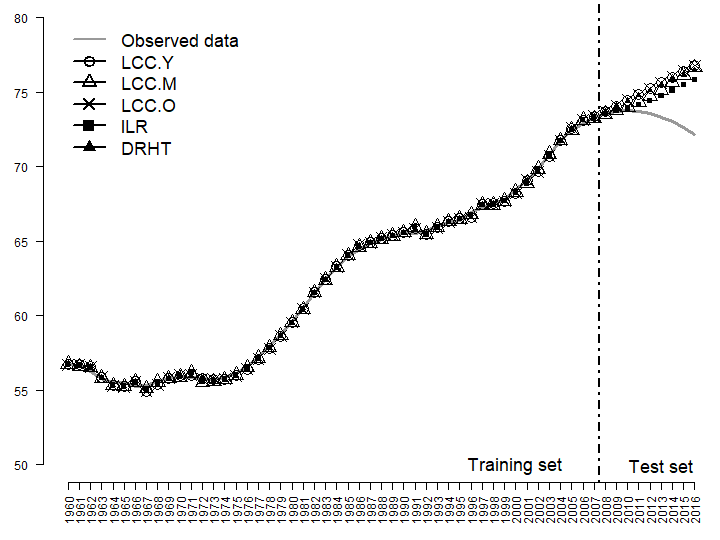

Supplement: S5 Fig — (PNG) [file pone.0212772.s005.png]

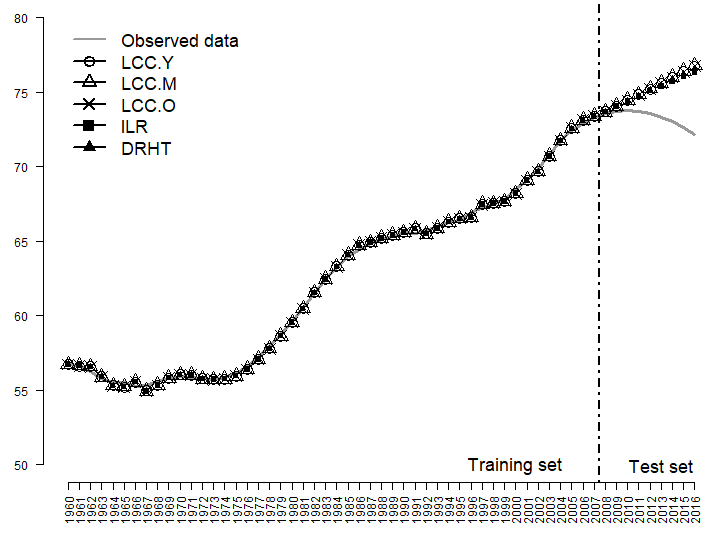

Supplement: S6 Fig — (PNG) [file pone.0212772.s006.png]

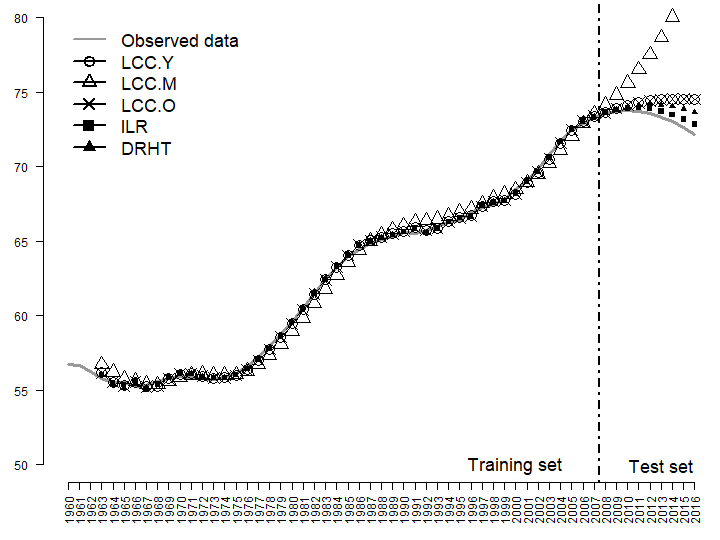

Supplement: S7 Fig — (PNG) [file pone.0212772.s007.png]

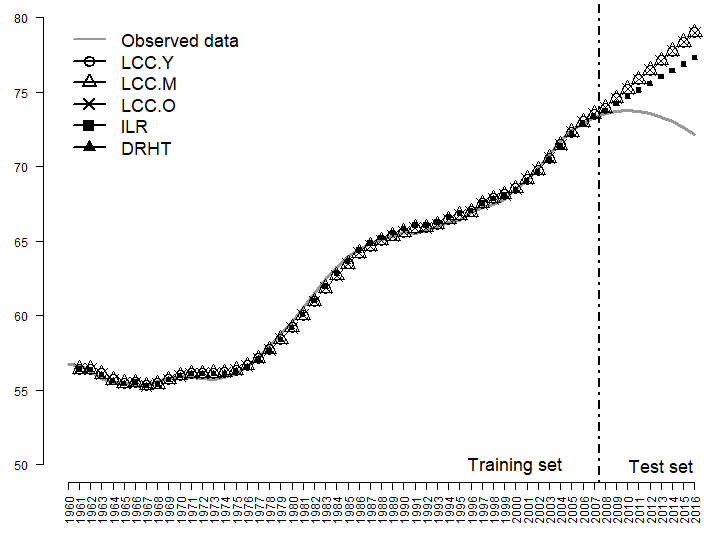

Supplement: S8 Fig — (PNG) [file pone.0212772.s008.png]

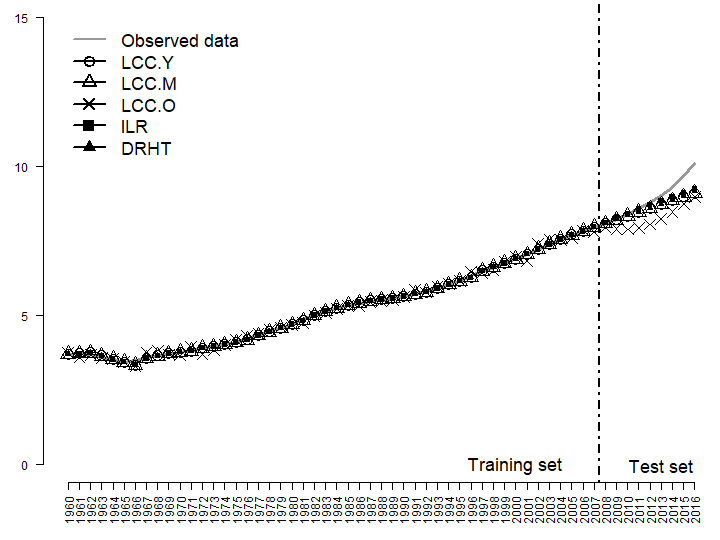

Supplement: S9 Fig — (PNG) [file pone.0212772.s009.png]

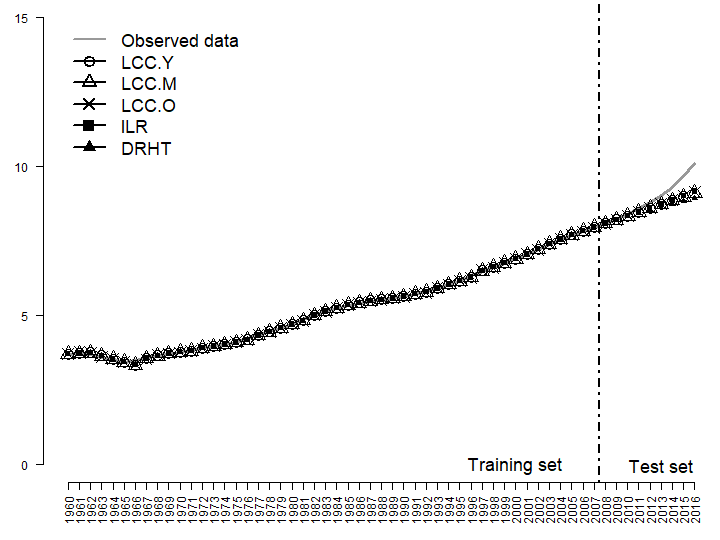

Supplement: S10 Fig — (PNG) [file pone.0212772.s010.png]

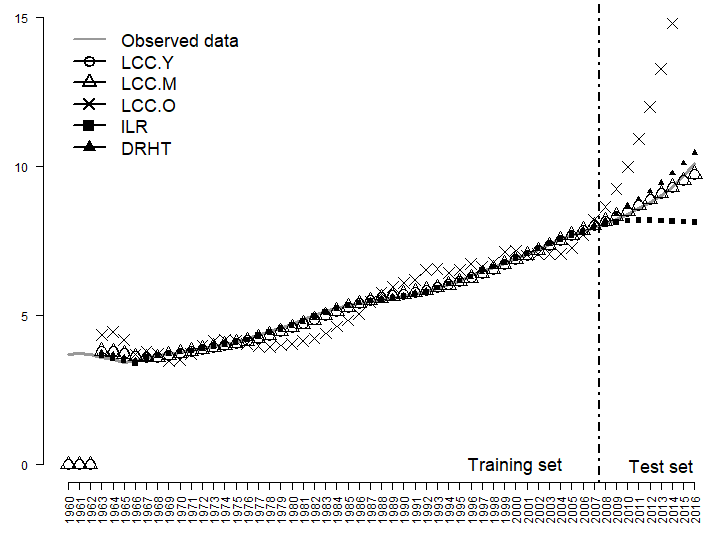

Supplement: S11 Fig — (PNG) [file pone.0212772.s011.png]

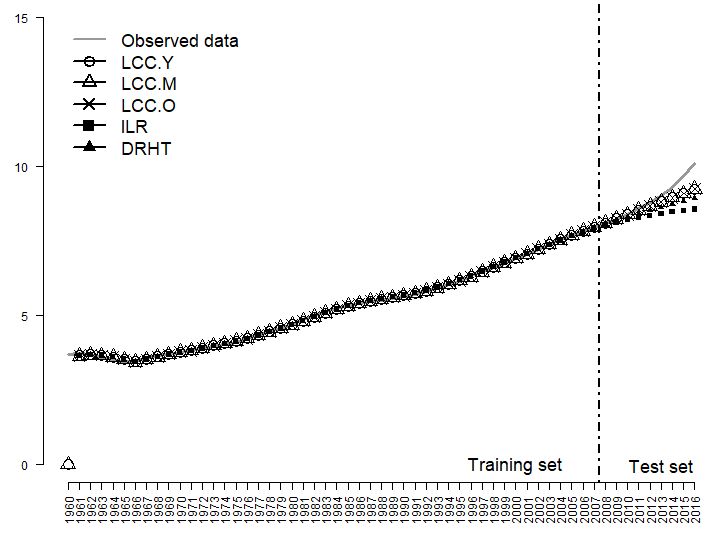

Supplement: S12 Fig — (PNG) [file pone.0212772.s012.png]

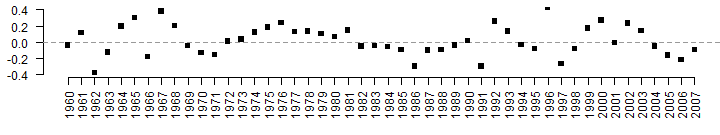

Supplement: S13 Fig — (PNG) [file pone.0212772.s013.png]

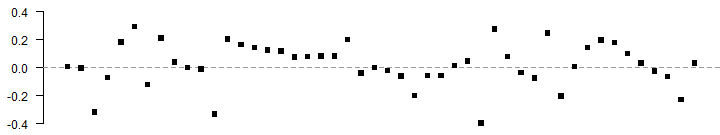

Supplement: S14 Fig — (PNG) [file pone.0212772.s014.png]

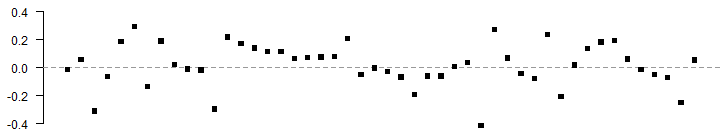

Supplement: S15 Fig — (PNG) [file pone.0212772.s015.png]

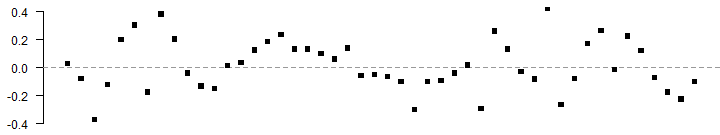

Supplement: S17 Fig — (PNG) [file pone.0212772.s017.png]

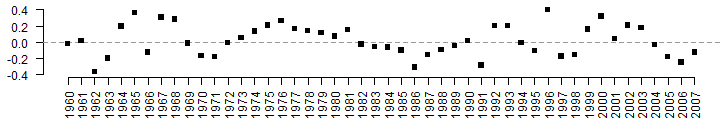

Supplement: S18 Fig — (PNG) [file pone.0212772.s018.png]

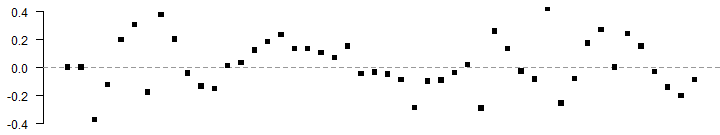

Supplement: S19 Fig — (PNG) [file pone.0212772.s019.png]

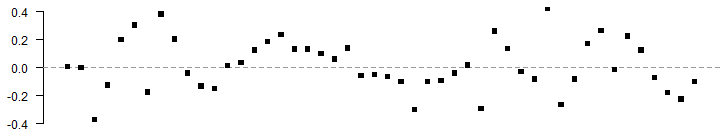

Supplement: S20 Fig — (PNG) [file pone.0212772.s020.png]

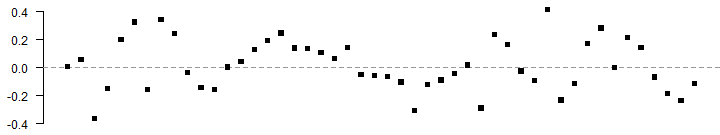

Supplement: S22 Fig — (PNG) [file pone.0212772.s022.png]

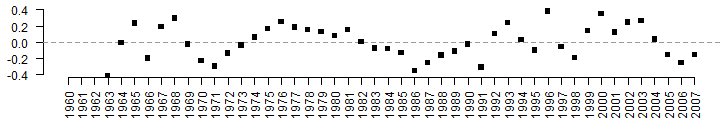

Supplement: S23 Fig — (PNG) [file pone.0212772.s023.png]

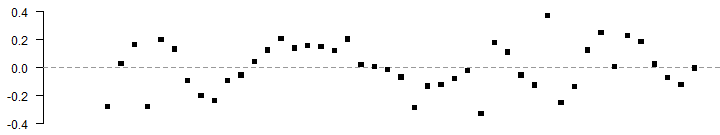

Supplement: S24 Fig — (PNG) [file pone.0212772.s024.png]

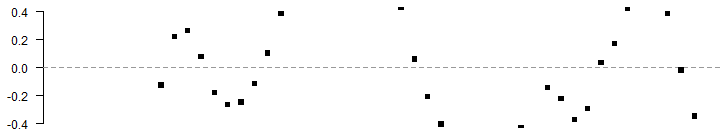

Supplement: S25 Fig — (PNG) [file pone.0212772.s025.png]

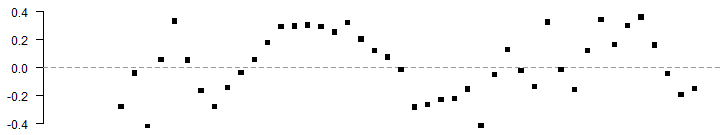

Supplement: S27 Fig — (PNG) [file pone.0212772.s027.png]

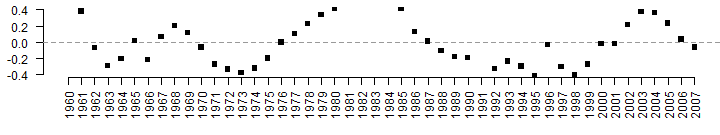

Supplement: S28 Fig — (PNG) [file pone.0212772.s028.png]

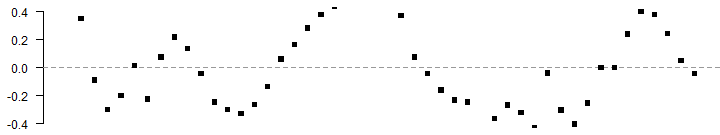

Supplement: S29 Fig — (PNG) [file pone.0212772.s029.png]

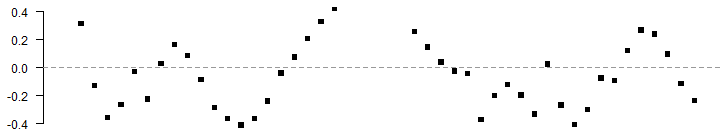

Supplement: S30 Fig — (PNG) [file pone.0212772.s030.png]

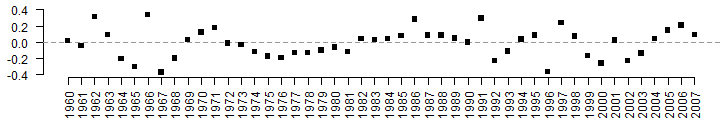

Supplement: S33 Fig — (PNG) [file pone.0212772.s033.png]

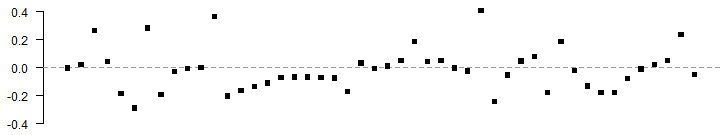

Supplement: S34 Fig — (PNG) [file pone.0212772.s034.png]

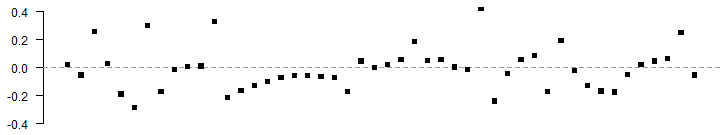

Supplement: S35 Fig — (PNG) [file pone.0212772.s035.png]

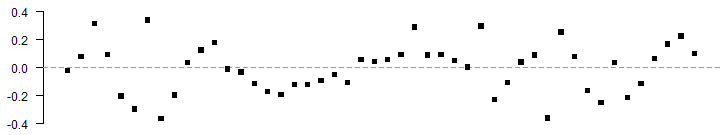

Supplement: S36 Fig — (PNG) [file pone.0212772.s036.png]

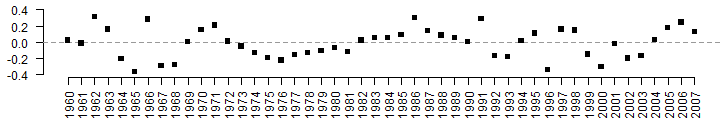

Supplement: S38 Fig — (PNG) [file pone.0212772.s038.png]

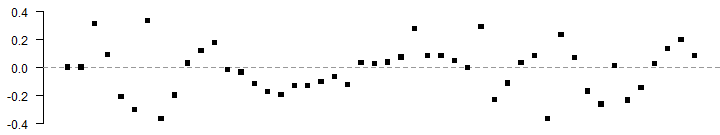

Supplement: S39 Fig — (PNG) [file pone.0212772.s039.png]

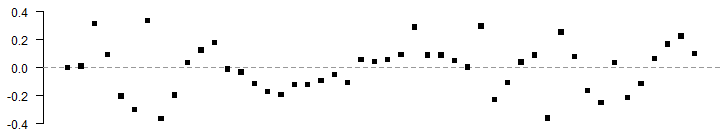

Supplement: S40 Fig — (PNG) [file pone.0212772.s040.png]

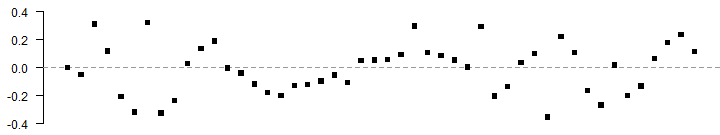

Supplement: S41 Fig — (PNG) [file pone.0212772.s041.png]

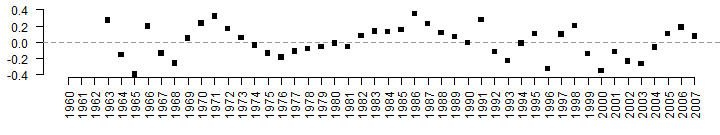

Supplement: S43 Fig — (PNG) [file pone.0212772.s043.png]

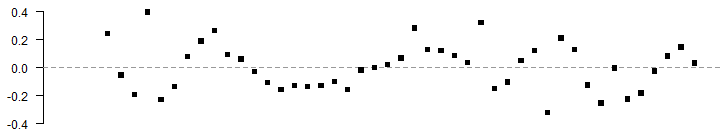

Supplement: S44 Fig — (PNG) [file pone.0212772.s044.png]

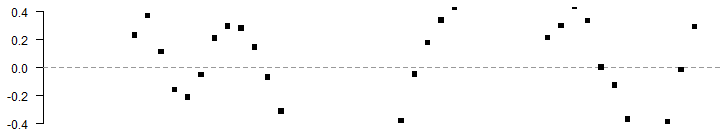

Supplement: S45 Fig — (PNG) [file pone.0212772.s045.png]

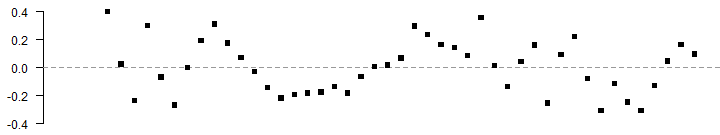

Supplement: S46 Fig — (PNG) [file pone.0212772.s046.png]

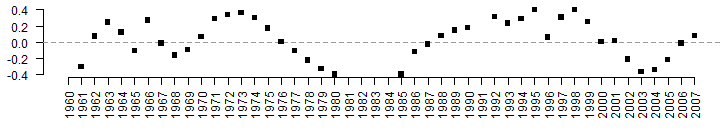

Supplement: S48 Fig — (PNG) [file pone.0212772.s048.png]

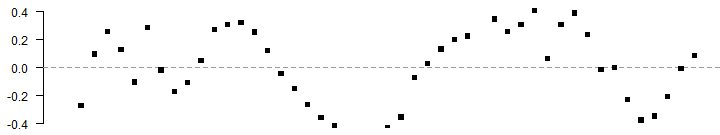

Supplement: S49 Fig — (PNG) [file pone.0212772.s049.png]

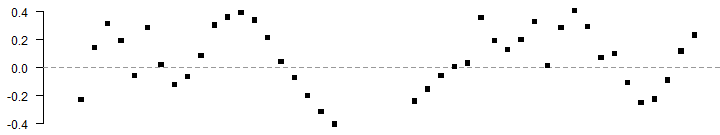

Supplement: S50 Fig — (PNG) [file pone.0212772.s050.png]

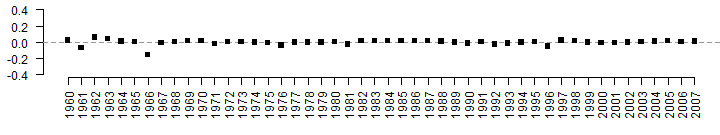

Supplement: S53 Fig — (PNG) [file pone.0212772.s053.png]

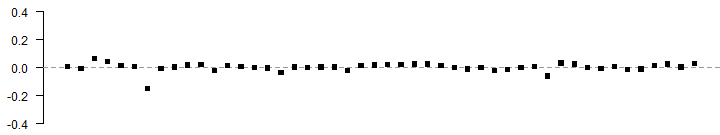

Supplement: S54 Fig — (PNG) [file pone.0212772.s054.png]

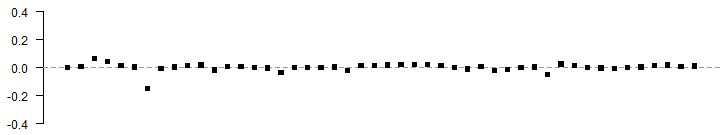

Supplement: S55 Fig — (PNG) [file pone.0212772.s055.png]

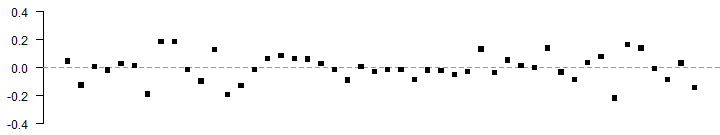

Supplement: S56 Fig — (PNG) [file pone.0212772.s056.png]

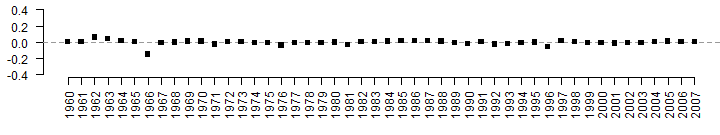

Supplement: S58 Fig — (PNG) [file pone.0212772.s058.png]

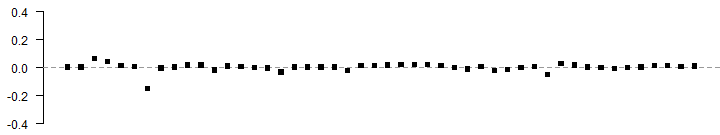

Supplement: S59 Fig — (PNG) [file pone.0212772.s059.png]

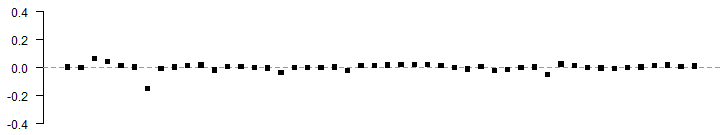

Supplement: S60 Fig — (PNG) [file pone.0212772.s060.png]

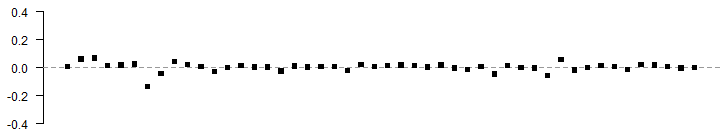

Supplement: S61 Fig — (PNG) [file pone.0212772.s061.png]

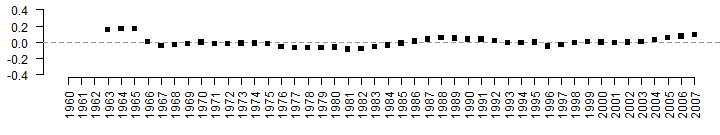

Supplement: S63 Fig — (PNG) [file pone.0212772.s063.png]

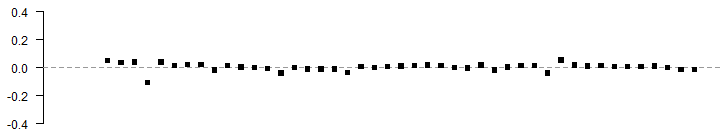

Supplement: S64 Fig — (PNG) [file pone.0212772.s064.png]

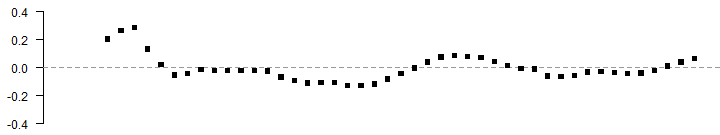

Supplement: S65 Fig — (PNG) [file pone.0212772.s065.png]

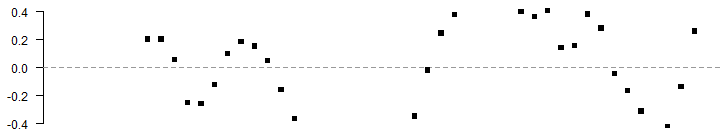

Supplement: S66 Fig — (PNG) [file pone.0212772.s066.png]

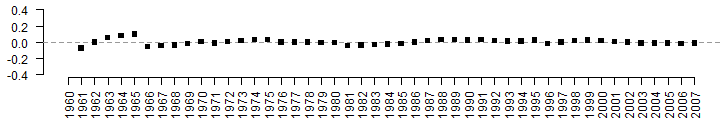

Supplement: S68 Fig — (PNG) [file pone.0212772.s068.png]

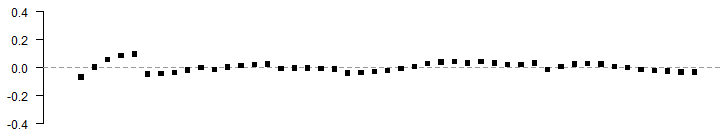

Supplement: S69 Fig — (PNG) [file pone.0212772.s069.png]

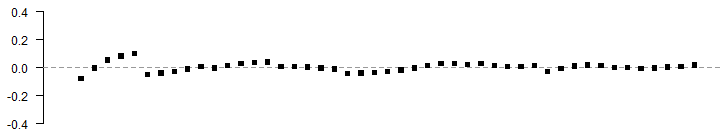

Supplement: S70 Fig — (PNG) [file pone.0212772.s070.png]

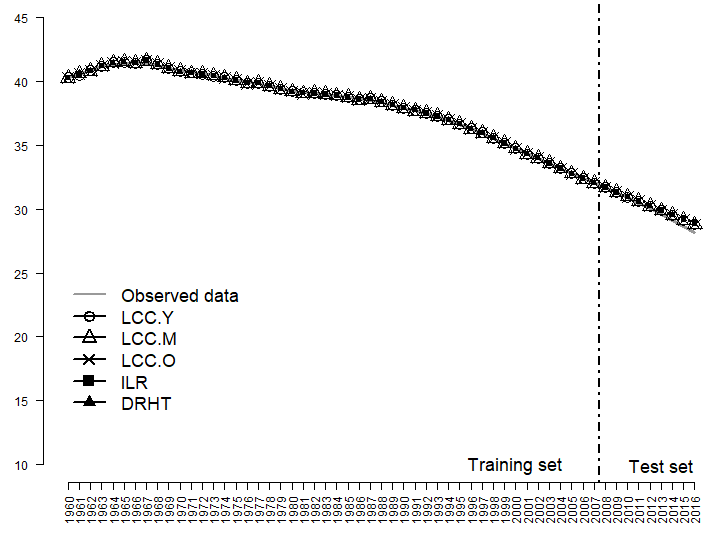

Supplement: S73 Fig — (PNG) [file pone.0212772.s073.png]

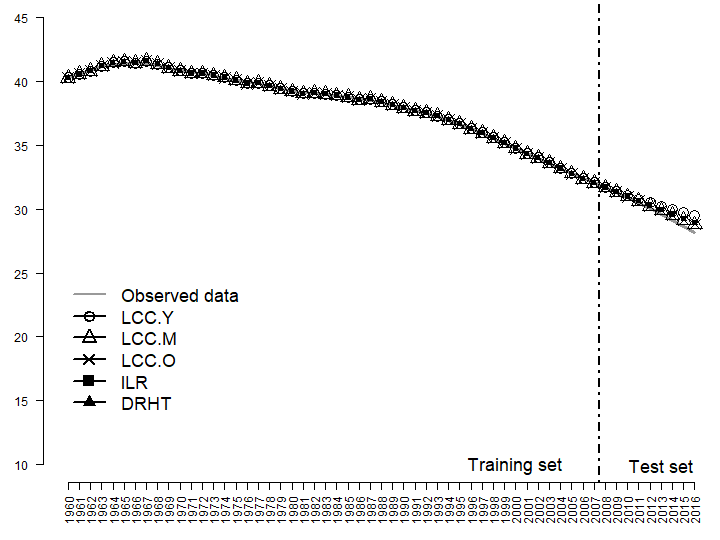

Supplement: S74 Fig — (PNG) [file pone.0212772.s074.png]

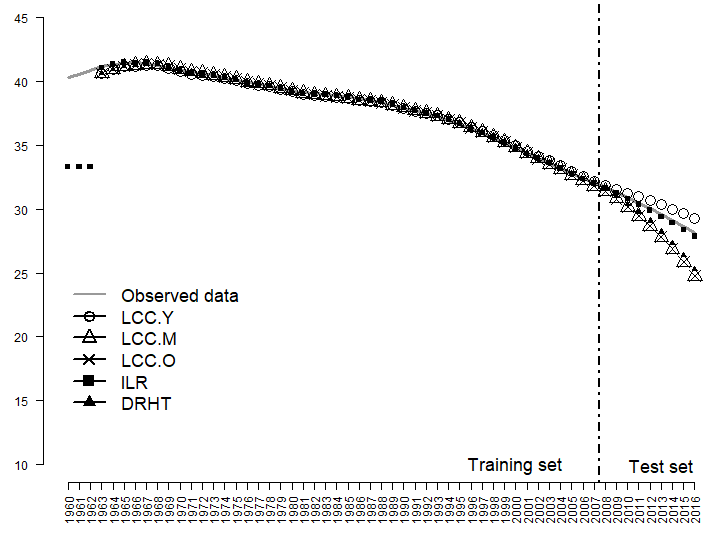

Supplement: S75 Fig — (PNG) [file pone.0212772.s075.png]

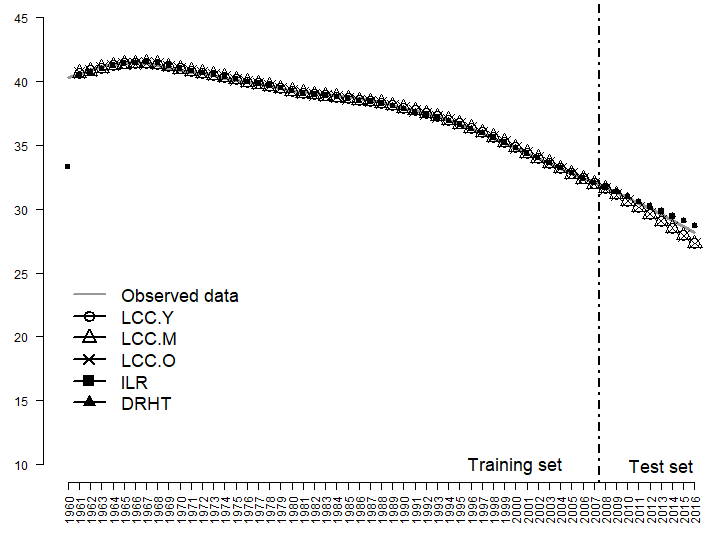

Supplement: S76 Fig — (PNG) [file pone.0212772.s076.png]

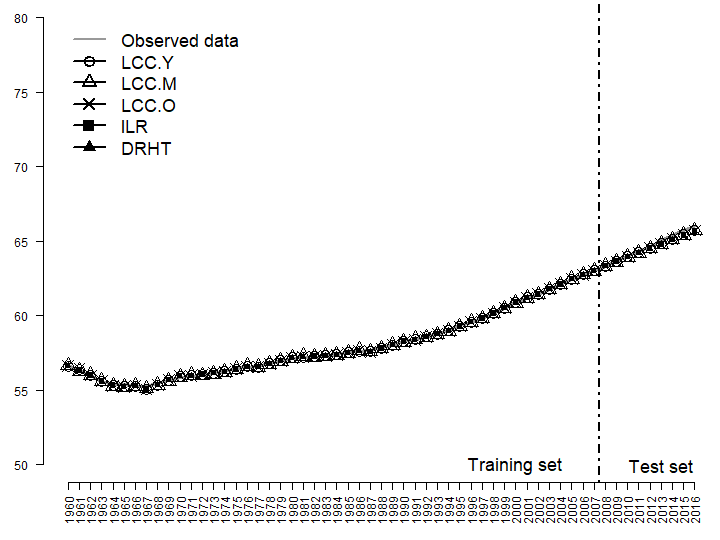

Supplement: S77 Fig — (PNG) [file pone.0212772.s077.png]

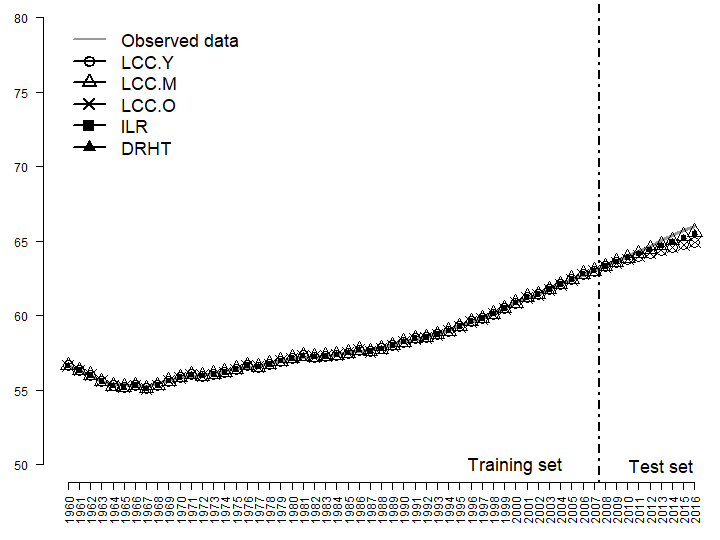

Supplement: S78 Fig — (PNG) [file pone.0212772.s078.png]

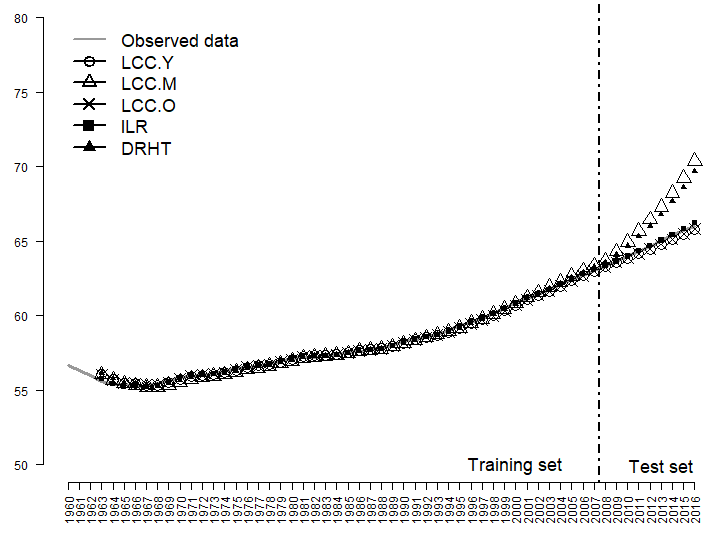

Supplement: S79 Fig — (PNG) [file pone.0212772.s079.png]

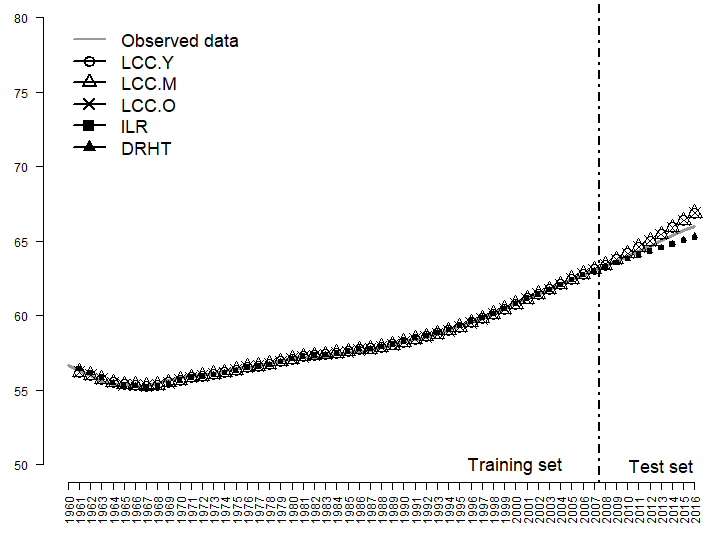

Supplement: S80 Fig — (PNG) [file pone.0212772.s080.png]

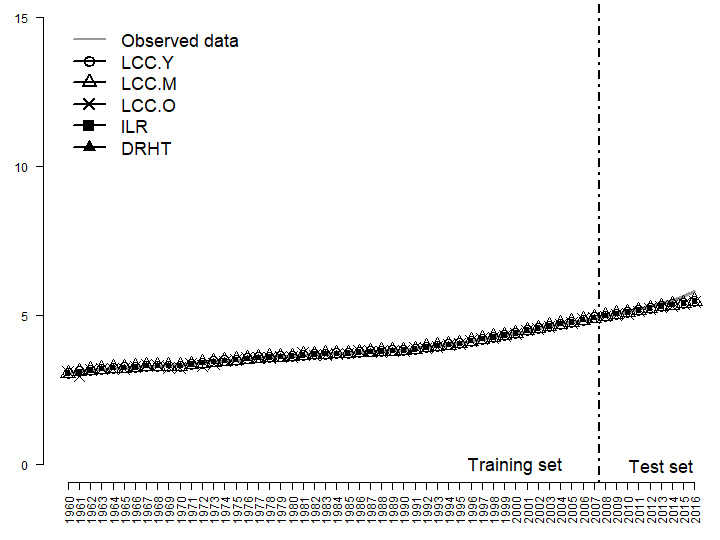

Supplement: S81 Fig — (PNG) [file pone.0212772.s081.png]

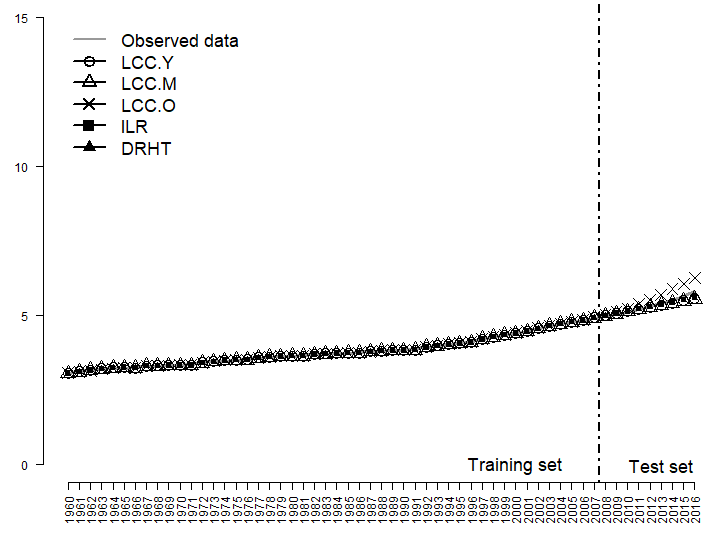

Supplement: S82 Fig — (PNG) [file pone.0212772.s082.png]

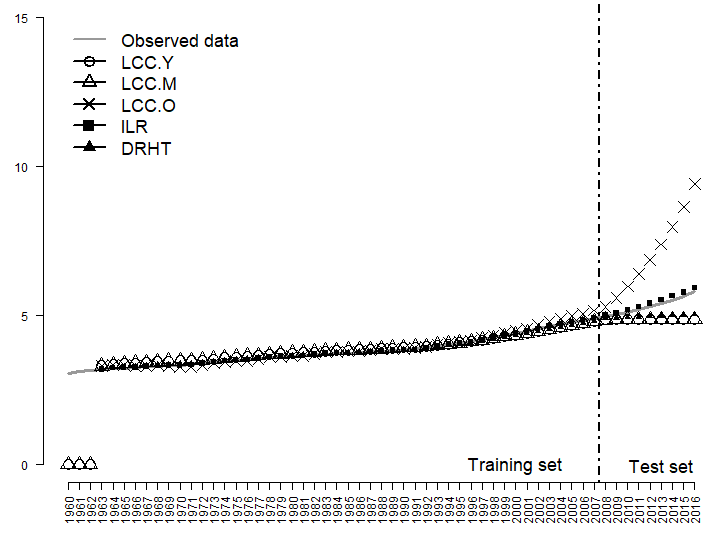

Supplement: S83 Fig — (PNG) [file pone.0212772.s083.png]

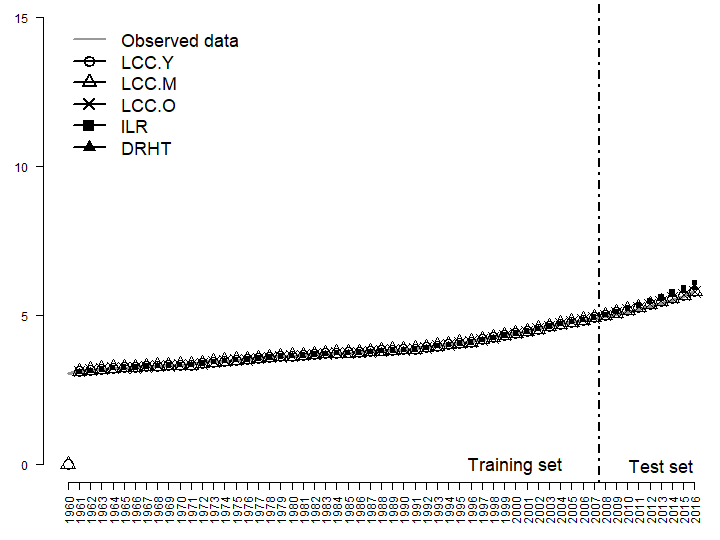

Supplement: S84 Fig — (PNG) [file pone.0212772.s084.png]

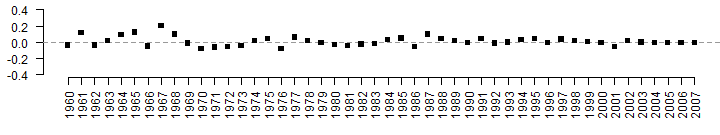

Supplement: S85 Fig — (PNG) [file pone.0212772.s085.png]

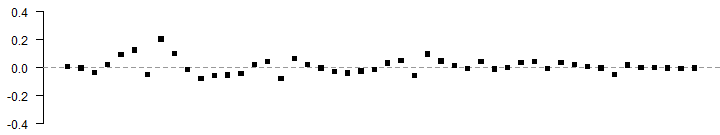

Supplement: S86 Fig — (PNG) [file pone.0212772.s086.png]

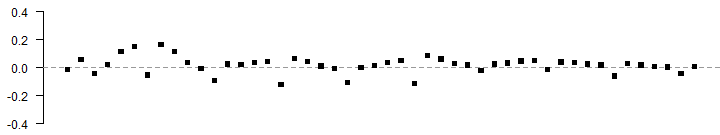

Supplement: S87 Fig — (PNG) [file pone.0212772.s087.png]

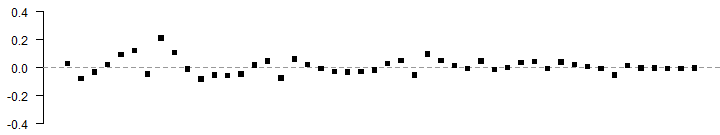

Supplement: S89 Fig — (PNG) [file pone.0212772.s089.png]

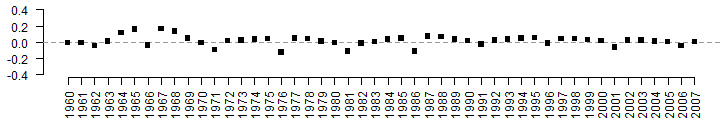

Supplement: S90 Fig — (PNG) [file pone.0212772.s090.png]

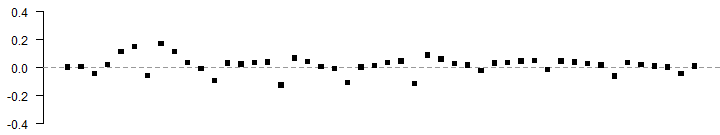

Supplement: S91 Fig — (PNG) [file pone.0212772.s091.png]

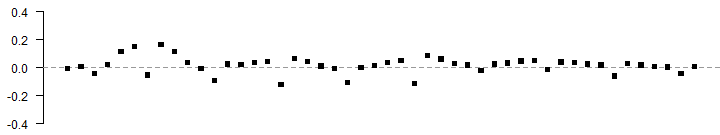

Supplement: S92 Fig — (PNG) [file pone.0212772.s092.png]

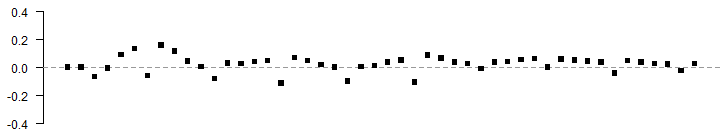

Supplement: S94 Fig — (PNG) [file pone.0212772.s094.png]

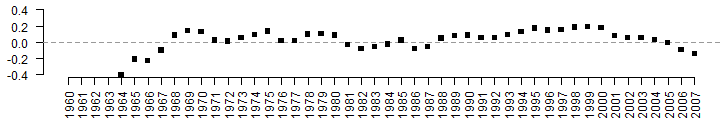

Supplement: S95 Fig — (PNG) [file pone.0212772.s095.png]

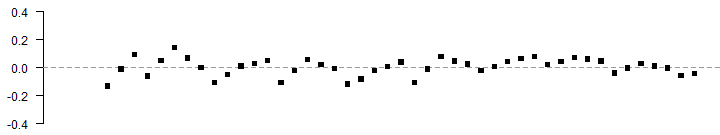

Supplement: S96 Fig — (PNG) [file pone.0212772.s096.png]

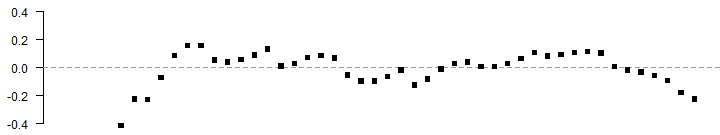

Supplement: S97 Fig — (PNG) [file pone.0212772.s097.png]

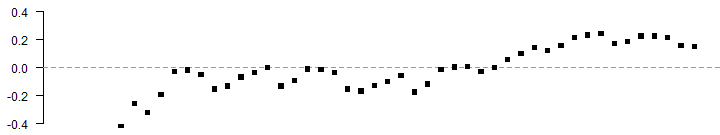

Supplement: S99 Fig — (PNG) [file pone.0212772.s099.png]

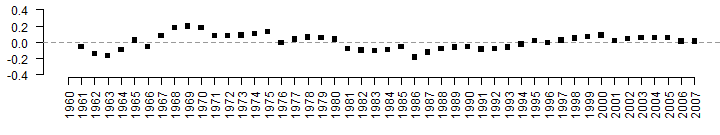

Supplement: S100 Fig — (PNG) [file pone.0212772.s100.png]

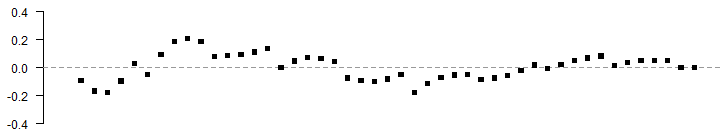

Supplement: S101 Fig — (PNG) [file pone.0212772.s101.png]

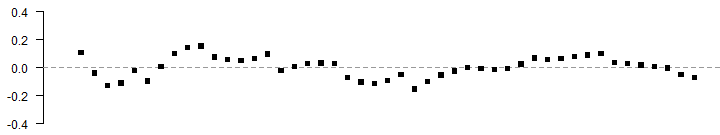

Supplement: S102 Fig — (PNG) [file pone.0212772.s102.png]

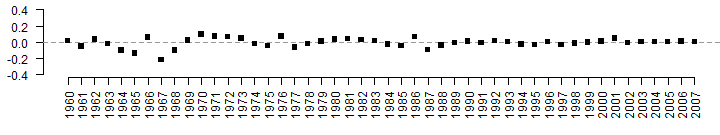

Supplement: S105 Fig — (PNG) [file pone.0212772.s105.png]

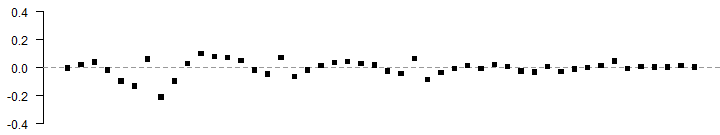

Supplement: S106 Fig — (PNG) [file pone.0212772.s106.png]

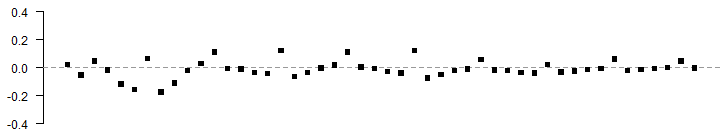

Supplement: S107 Fig — (PNG) [file pone.0212772.s107.png]

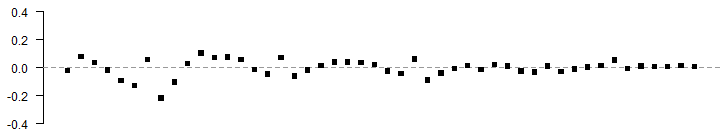

Supplement: S108 Fig — (PNG) [file pone.0212772.s108.png]

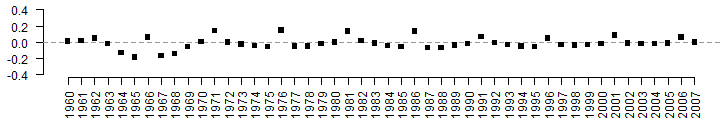

Supplement: S110 Fig — (PNG) [file pone.0212772.s110.png]

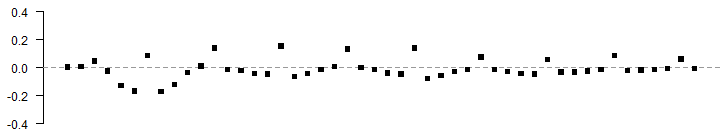

Supplement: S111 Fig — (PNG) [file pone.0212772.s111.png]

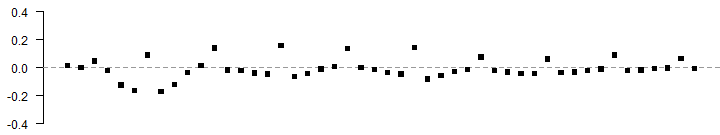

Supplement: S112 Fig — (PNG) [file pone.0212772.s112.png]

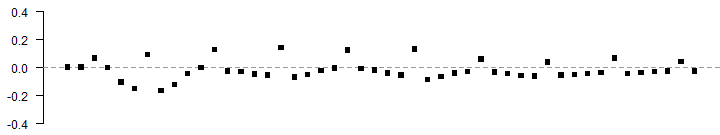

Supplement: S113 Fig — (PNG) [file pone.0212772.s113.png]

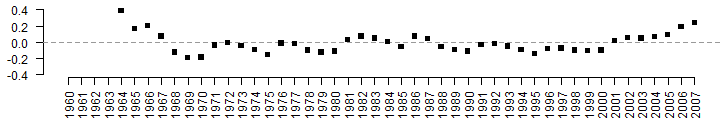

Supplement: S115 Fig — (PNG) [file pone.0212772.s115.png]

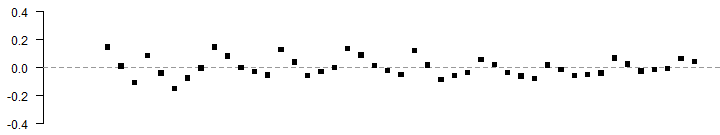

Supplement: S116 Fig — (PNG) [file pone.0212772.s116.png]

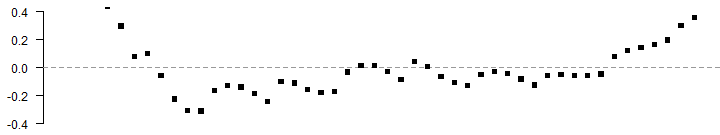

Supplement: S117 Fig — (PNG) [file pone.0212772.s117.png]

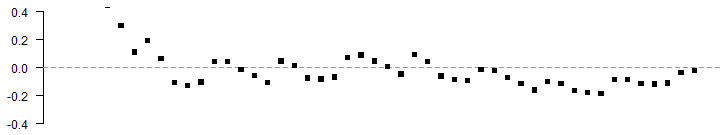

Supplement: S118 Fig — (PNG) [file pone.0212772.s118.png]

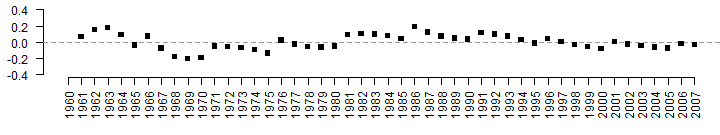

Supplement: S120 Fig — (PNG) [file pone.0212772.s120.png]

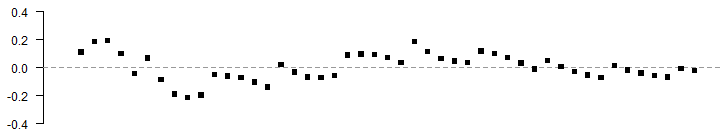

Supplement: S121 Fig — (PNG) [file pone.0212772.s121.png]

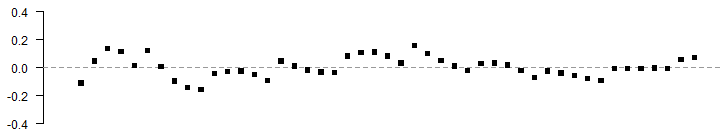

Supplement: S122 Fig — (PNG) [file pone.0212772.s122.png]

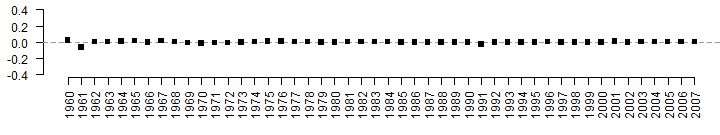

Supplement: S125 Fig — (PNG) [file pone.0212772.s125.png]
