# Supplementary figures and images for: Predicting population age structures of China, India, and Vietnam by 2030 based on compositional data (part 2 of 2)
Source: PLoS One. 2019 Apr 11;14(4):e0212772. doi: 10.1371/journal.pone.0212772 (PMC6459537; doi:10.1371/journal.pone.0212772)

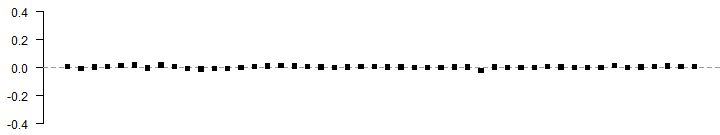

Supplement: S126 Fig — (PNG) [file pone.0212772.s126.png]

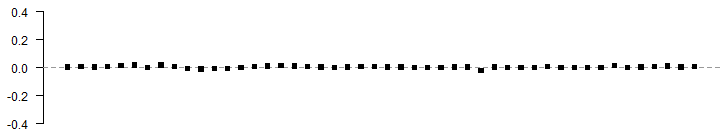

Supplement: S127 Fig — (PNG) [file pone.0212772.s127.png]

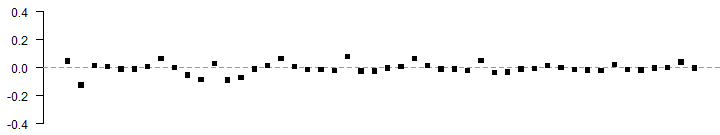

Supplement: S128 Fig — (PNG) [file pone.0212772.s128.png]

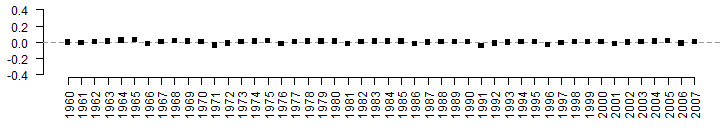

Supplement: S130 Fig — (PNG) [file pone.0212772.s130.png]

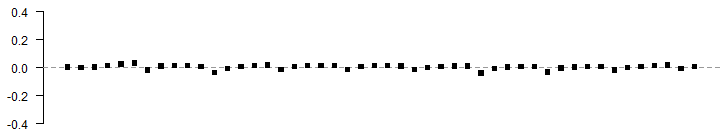

Supplement: S131 Fig — (PNG) [file pone.0212772.s131.png]

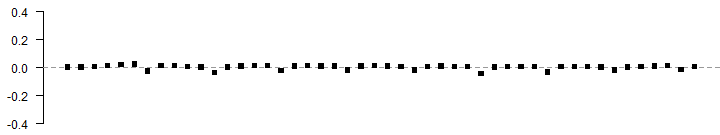

Supplement: S132 Fig — (PNG) [file pone.0212772.s132.png]

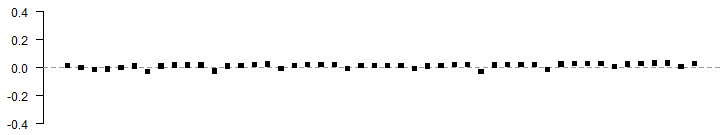

Supplement: S133 Fig — (PNG) [file pone.0212772.s133.png]

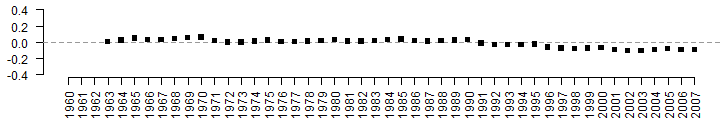

Supplement: S135 Fig — (PNG) [file pone.0212772.s135.png]

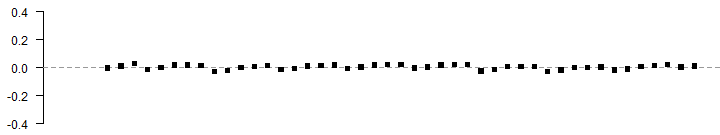

Supplement: S136 Fig — (PNG) [file pone.0212772.s136.png]

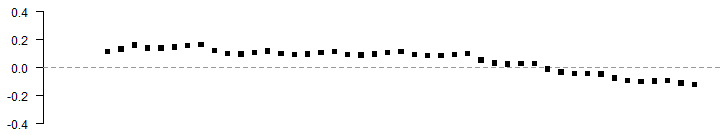

Supplement: S137 Fig — (PNG) [file pone.0212772.s137.png]

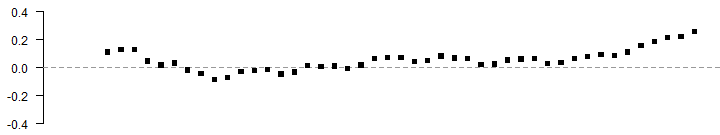

Supplement: S138 Fig — (PNG) [file pone.0212772.s138.png]

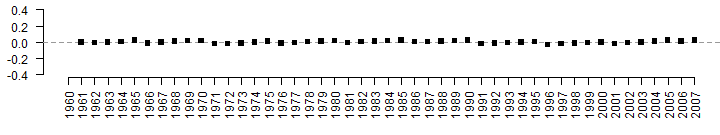

Supplement: S140 Fig — (PNG) [file pone.0212772.s140.png]

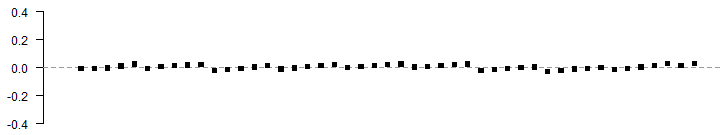

Supplement: S141 Fig — (PNG) [file pone.0212772.s141.png]

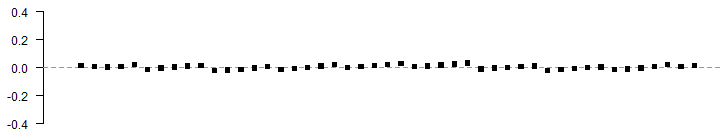

Supplement: S142 Fig — (PNG) [file pone.0212772.s142.png]

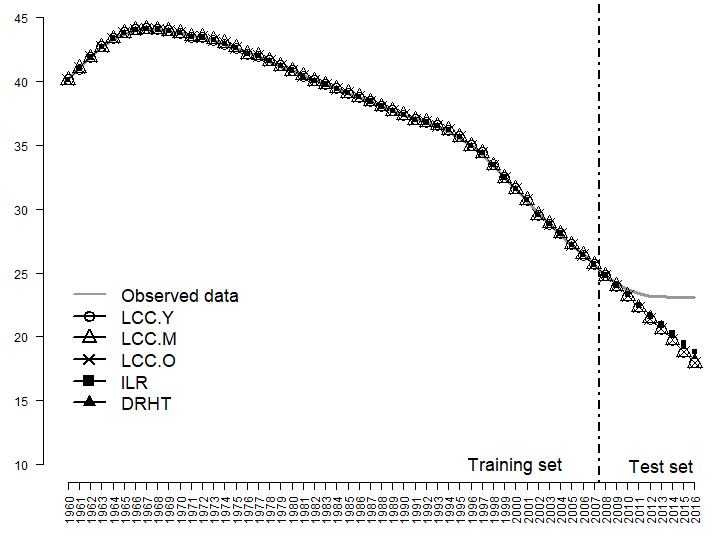

Supplement: S145 Fig — (PNG) [file pone.0212772.s145.png]

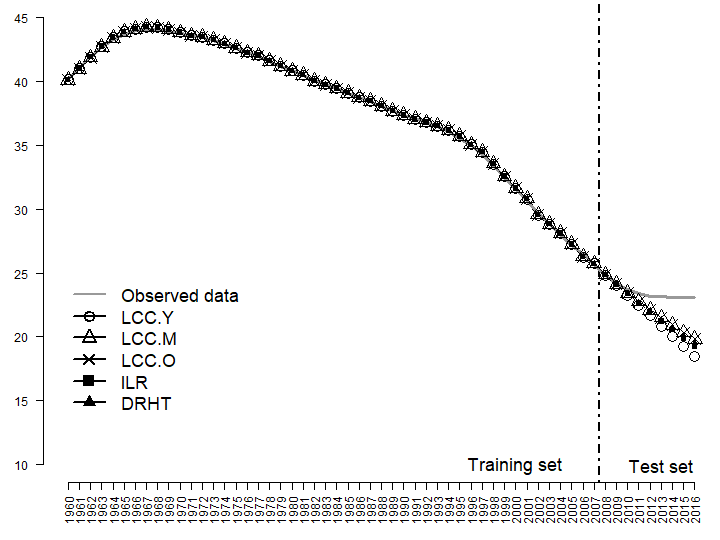

Supplement: S146 Fig — (PNG) [file pone.0212772.s146.png]

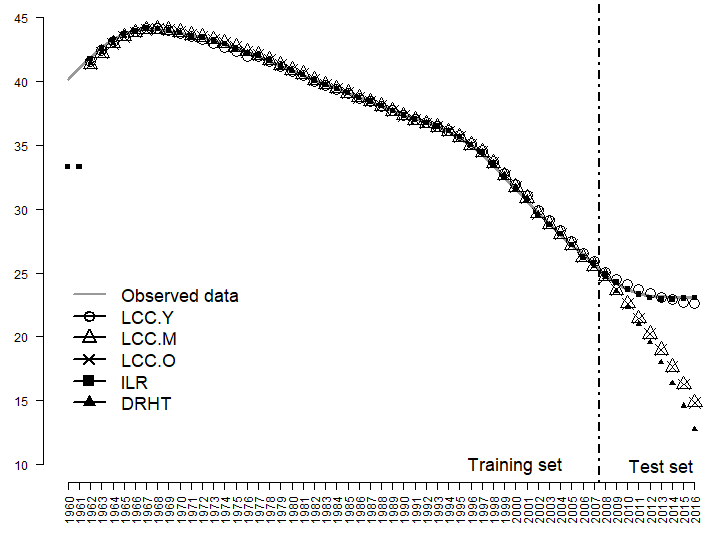

Supplement: S147 Fig — (PNG) [file pone.0212772.s147.png]

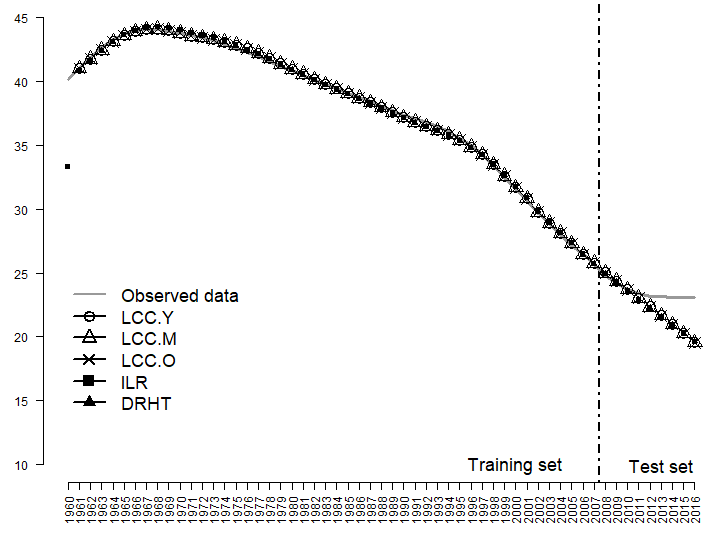

Supplement: S148 Fig — (PNG) [file pone.0212772.s148.png]

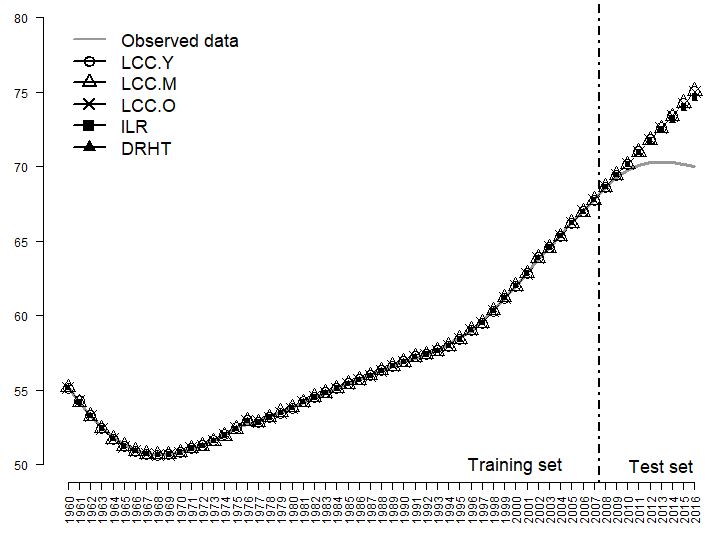

Supplement: S149 Fig — (PNG) [file pone.0212772.s149.png]

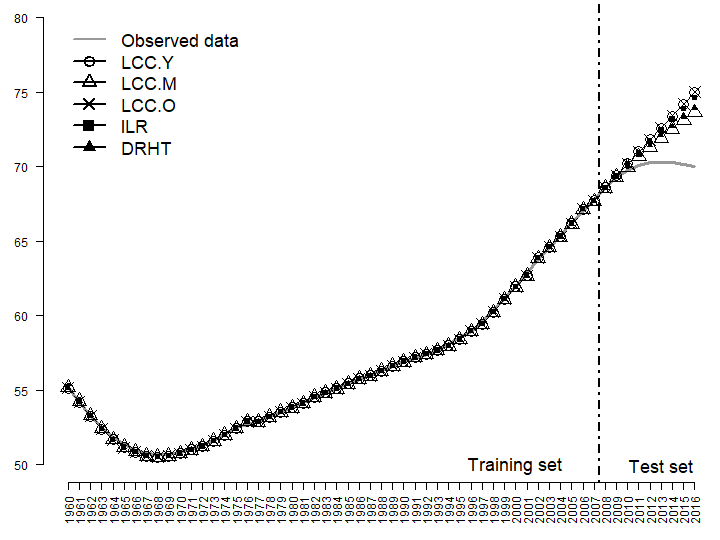

Supplement: S150 Fig — (PNG) [file pone.0212772.s150.png]

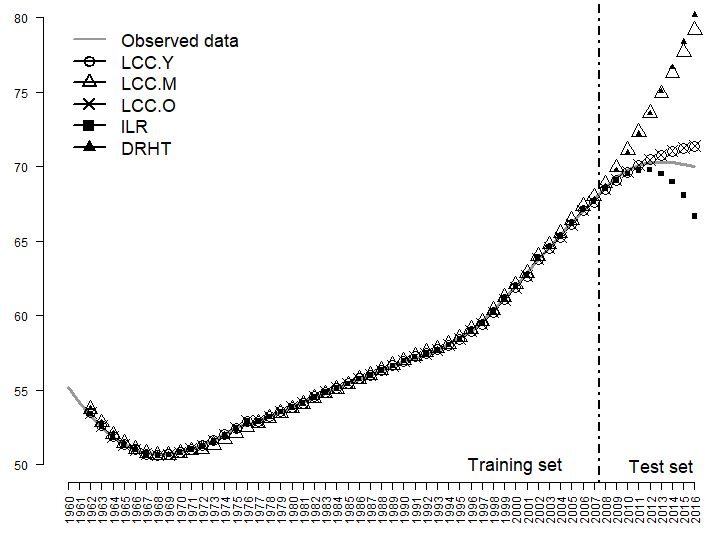

Supplement: S151 Fig — (PNG) [file pone.0212772.s151.png]

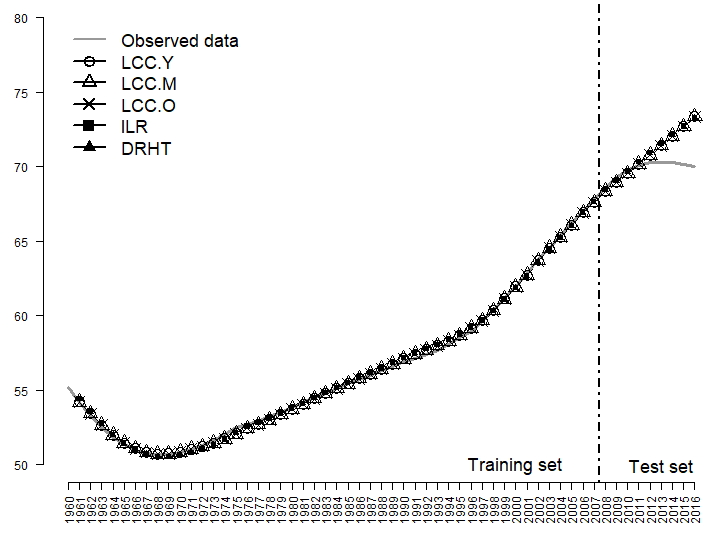

Supplement: S152 Fig — (PNG) [file pone.0212772.s152.png]

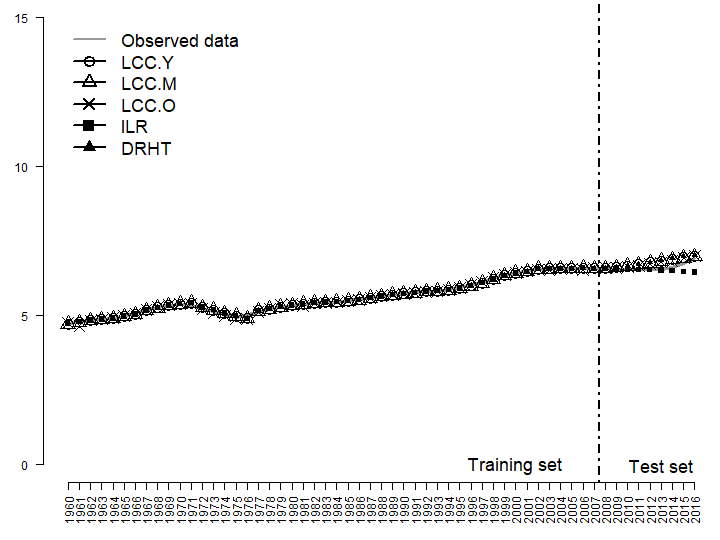

Supplement: S153 Fig — (PNG) [file pone.0212772.s153.png]

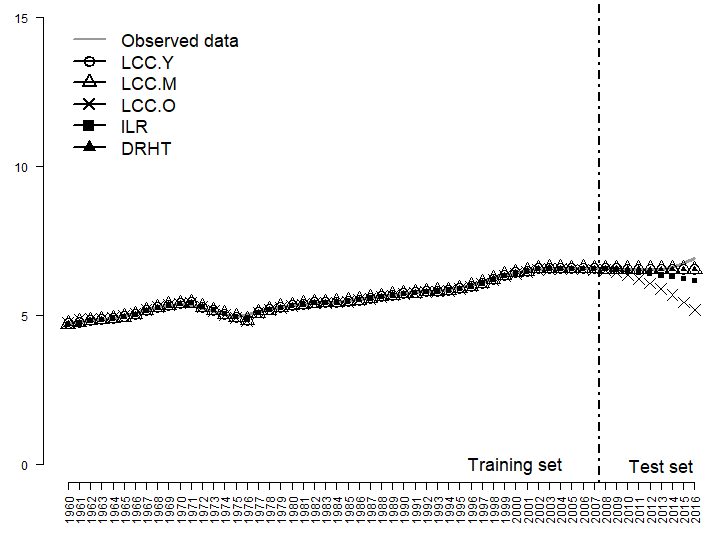

Supplement: S154 Fig — (PNG) [file pone.0212772.s154.png]

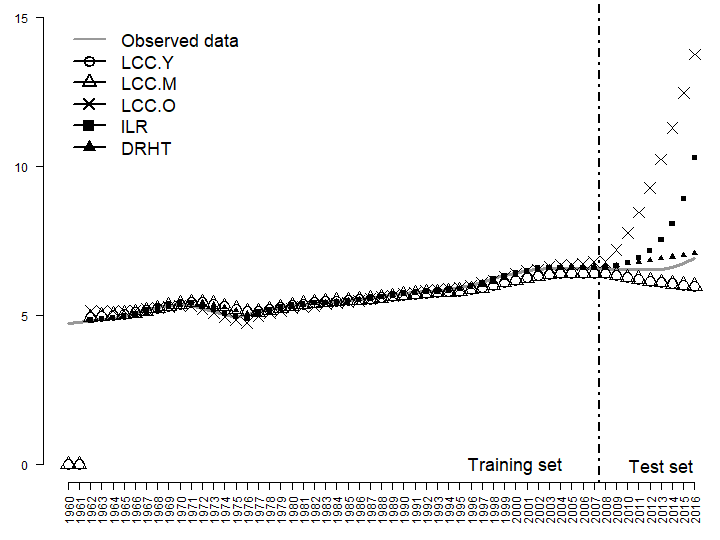

Supplement: S155 Fig — (PNG) [file pone.0212772.s155.png]

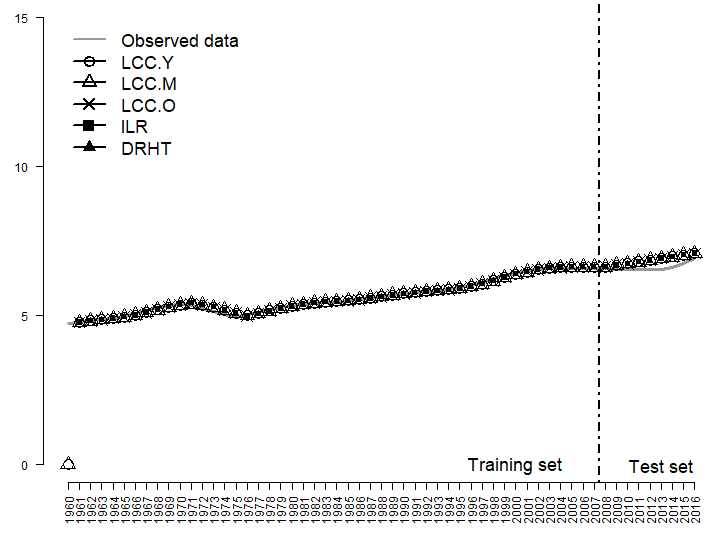

Supplement: S156 Fig — (PNG) [file pone.0212772.s156.png]

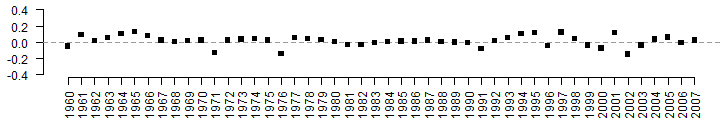

Supplement: S157 Fig — (PNG) [file pone.0212772.s157.png]

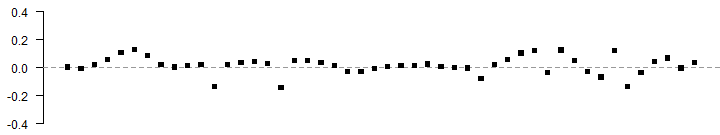

Supplement: S158 Fig — (PNG) [file pone.0212772.s158.png]

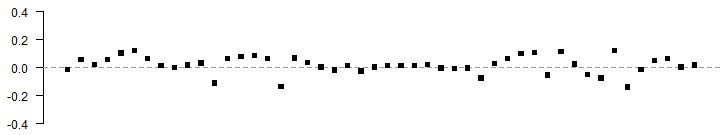

Supplement: S159 Fig — (PNG) [file pone.0212772.s159.png]

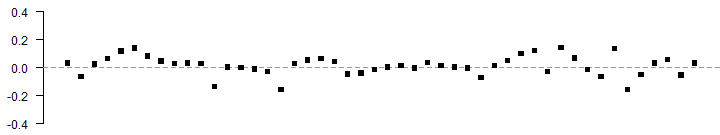

Supplement: S161 Fig — (PNG) [file pone.0212772.s161.png]

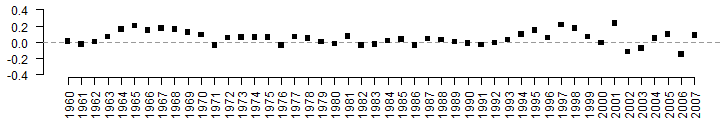

Supplement: S162 Fig — (PNG) [file pone.0212772.s162.png]

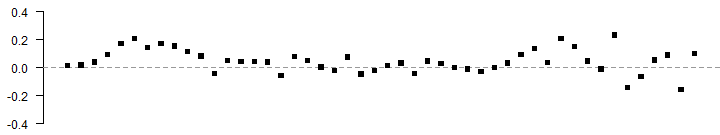

Supplement: S163 Fig — (PNG) [file pone.0212772.s163.png]

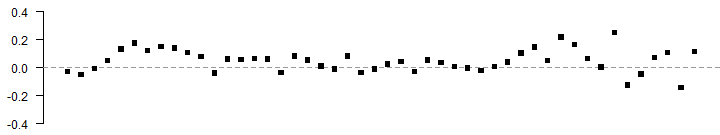

Supplement: S164 Fig — (PNG) [file pone.0212772.s164.png]

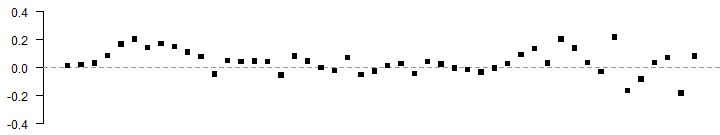

Supplement: S166 Fig — (PNG) [file pone.0212772.s166.png]

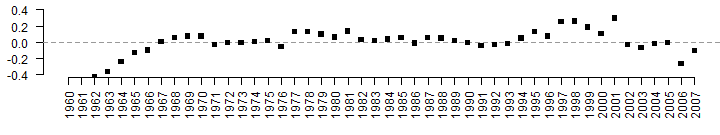

Supplement: S167 Fig — (PNG) [file pone.0212772.s167.png]

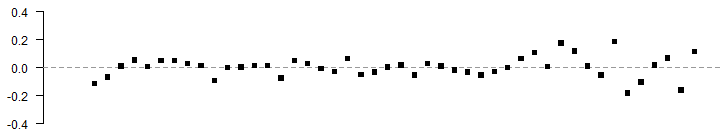

Supplement: S168 Fig — (PNG) [file pone.0212772.s168.png]

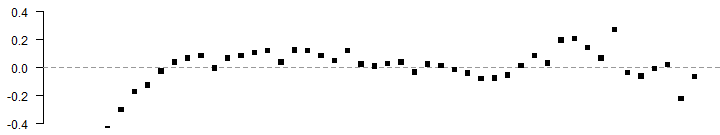

Supplement: S169 Fig — (PNG) [file pone.0212772.s169.png]

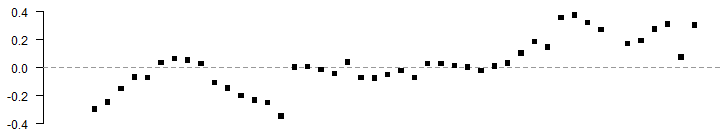

Supplement: S171 Fig — (PNG) [file pone.0212772.s171.png]

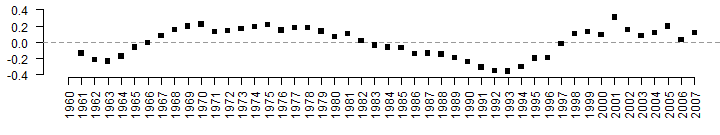

Supplement: S172 Fig — (PNG) [file pone.0212772.s172.png]

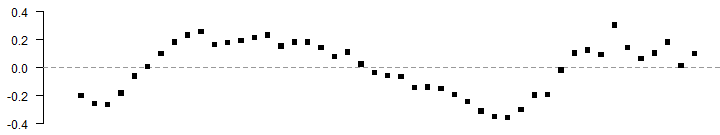

Supplement: S173 Fig — (PNG) [file pone.0212772.s173.png]

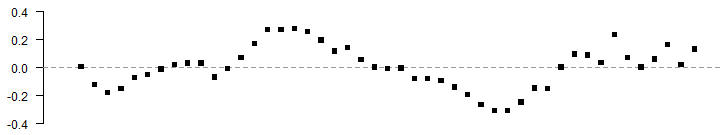

Supplement: S174 Fig — (PNG) [file pone.0212772.s174.png]

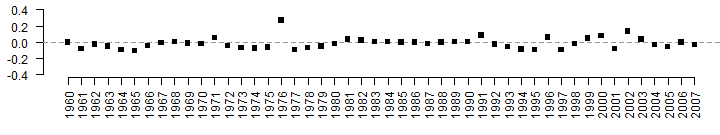

Supplement: S177 Fig — (PNG) [file pone.0212772.s177.png]

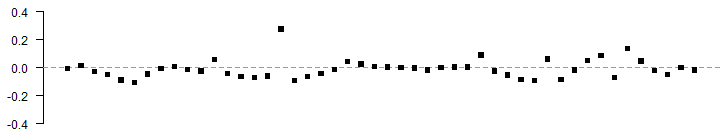

Supplement: S178 Fig — (PNG) [file pone.0212772.s178.png]

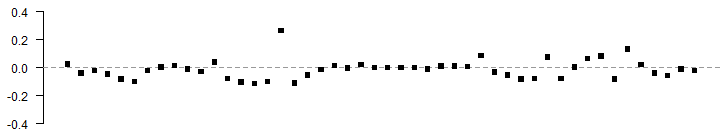

Supplement: S179 Fig — (PNG) [file pone.0212772.s179.png]

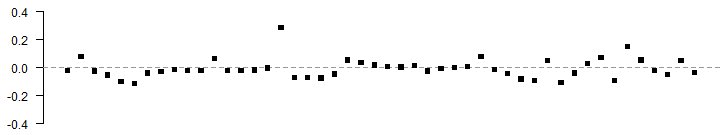

Supplement: S180 Fig — (PNG) [file pone.0212772.s180.png]

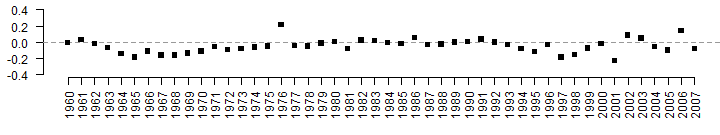

Supplement: S182 Fig — (PNG) [file pone.0212772.s182.png]

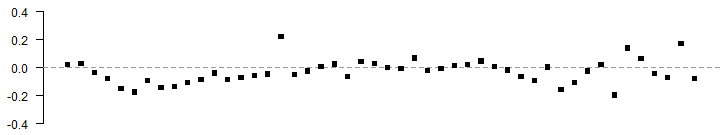

Supplement: S183 Fig — (PNG) [file pone.0212772.s183.png]

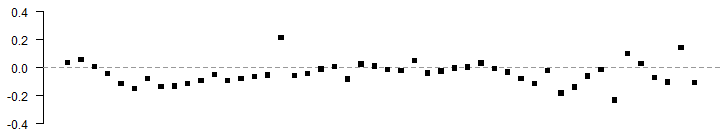

Supplement: S184 Fig — (PNG) [file pone.0212772.s184.png]

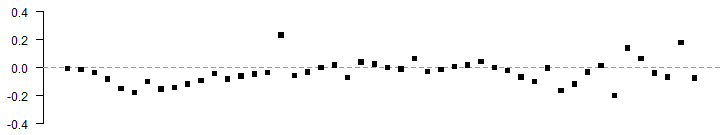

Supplement: S185 Fig — (PNG) [file pone.0212772.s185.png]

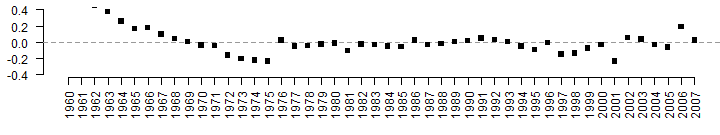

Supplement: S187 Fig — (PNG) [file pone.0212772.s187.png]

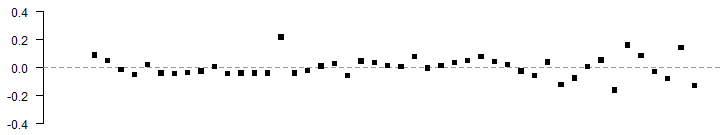

Supplement: S188 Fig — (PNG) [file pone.0212772.s188.png]

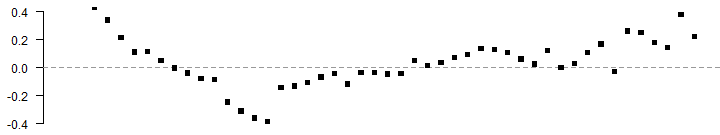

Supplement: S189 Fig — (PNG) [file pone.0212772.s189.png]

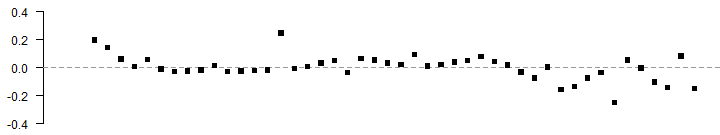

Supplement: S190 Fig — (PNG) [file pone.0212772.s190.png]

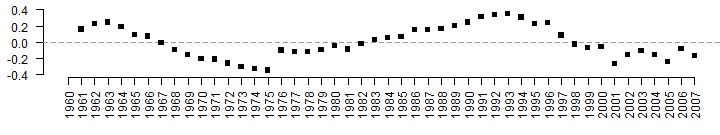

Supplement: S192 Fig — (PNG) [file pone.0212772.s192.png]

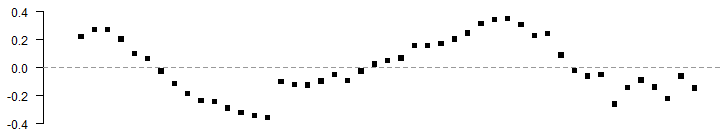

Supplement: S193 Fig — (PNG) [file pone.0212772.s193.png]

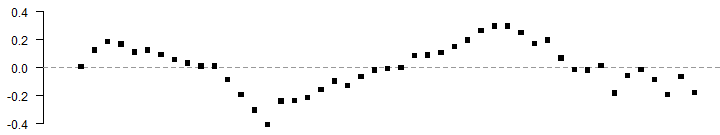

Supplement: S194 Fig — (PNG) [file pone.0212772.s194.png]

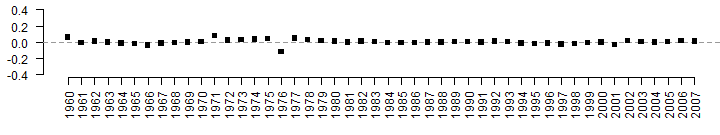

Supplement: S197 Fig — (PNG) [file pone.0212772.s197.png]

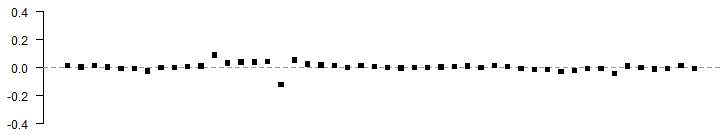

Supplement: S198 Fig — (PNG) [file pone.0212772.s198.png]

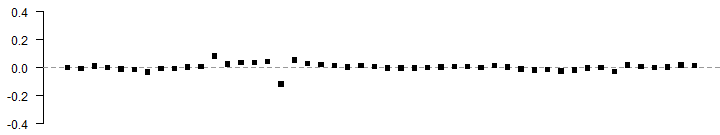

Supplement: S199 Fig — (PNG) [file pone.0212772.s199.png]

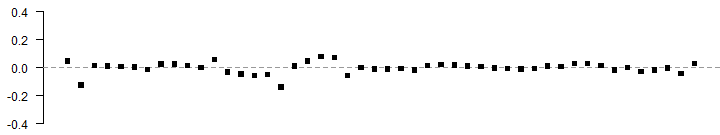

Supplement: S200 Fig — (PNG) [file pone.0212772.s200.png]

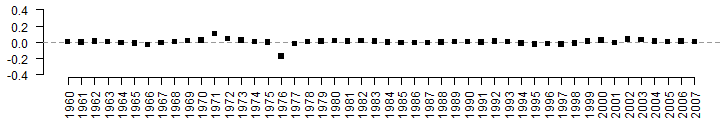

Supplement: S202 Fig — (PNG) [file pone.0212772.s202.png]

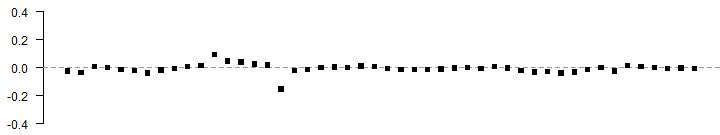

Supplement: S203 Fig — (PNG) [file pone.0212772.s203.png]

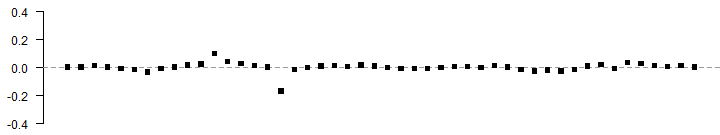

Supplement: S204 Fig — (PNG) [file pone.0212772.s204.png]

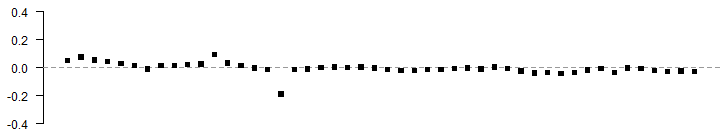

Supplement: S205 Fig — (PNG) [file pone.0212772.s205.png]

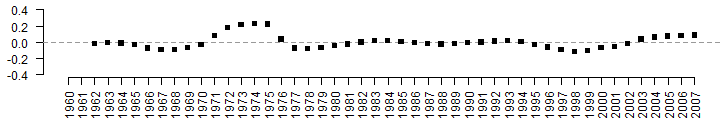

Supplement: S207 Fig — (PNG) [file pone.0212772.s207.png]

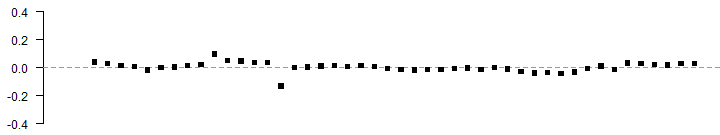

Supplement: S208 Fig — (PNG) [file pone.0212772.s208.png]

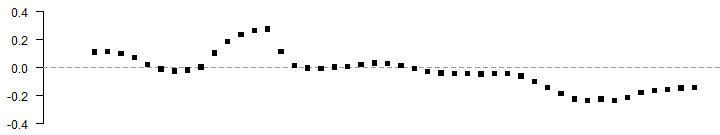

Supplement: S209 Fig — (PNG) [file pone.0212772.s209.png]

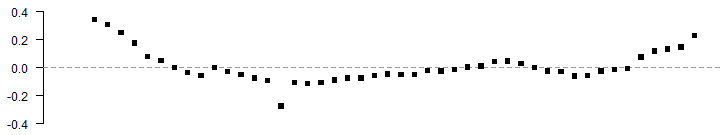

Supplement: S210 Fig — (PNG) [file pone.0212772.s210.png]

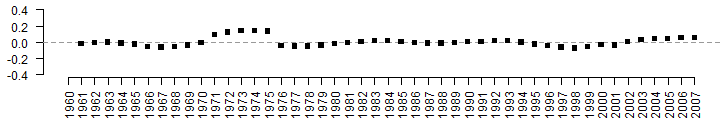

Supplement: S212 Fig — (PNG) [file pone.0212772.s212.png]

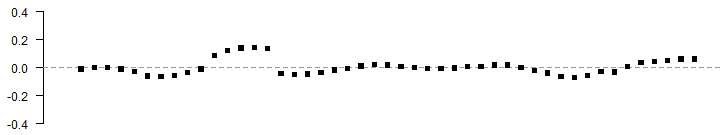

Supplement: S213 Fig — (PNG) [file pone.0212772.s213.png]

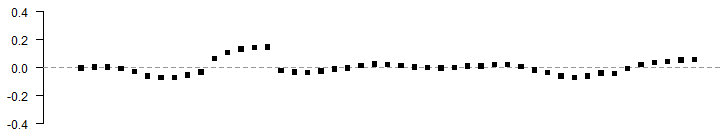

Supplement: S214 Fig — (PNG) [file pone.0212772.s214.png]

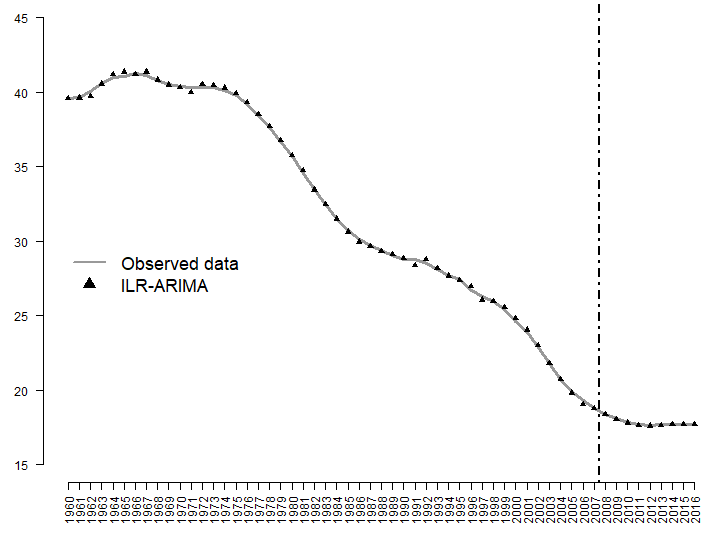

Supplement: S217 Fig — (PNG) [file pone.0212772.s217.png]

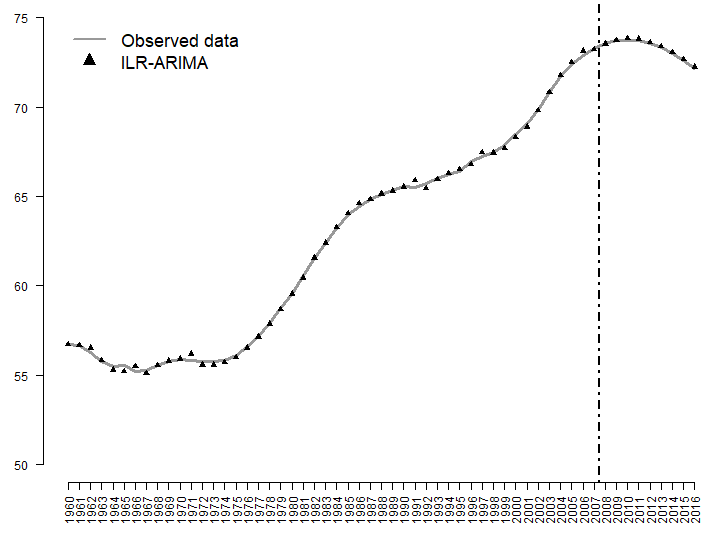

Supplement: S218 Fig — (PNG) [file pone.0212772.s218.png]

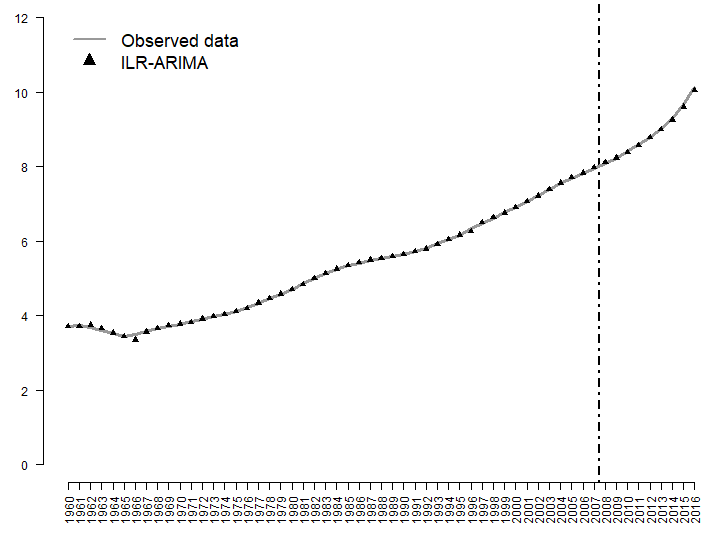

Supplement: S219 Fig — (PNG) [file pone.0212772.s219.png]

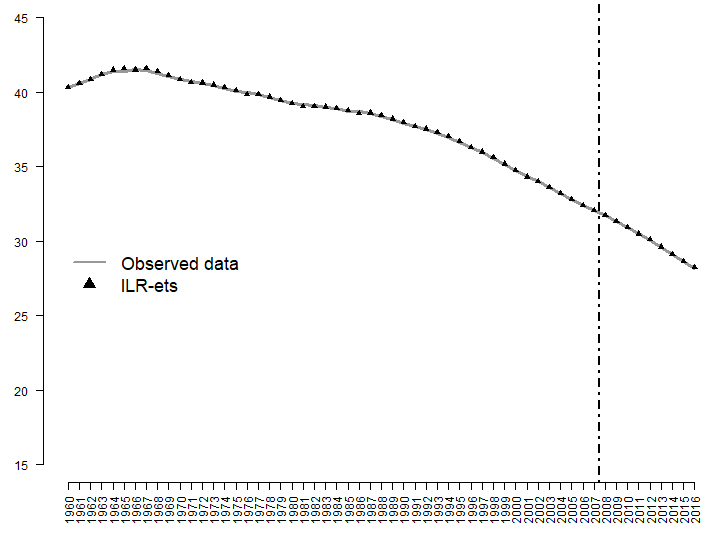

Supplement: S220 Fig — (PNG) [file pone.0212772.s220.png]

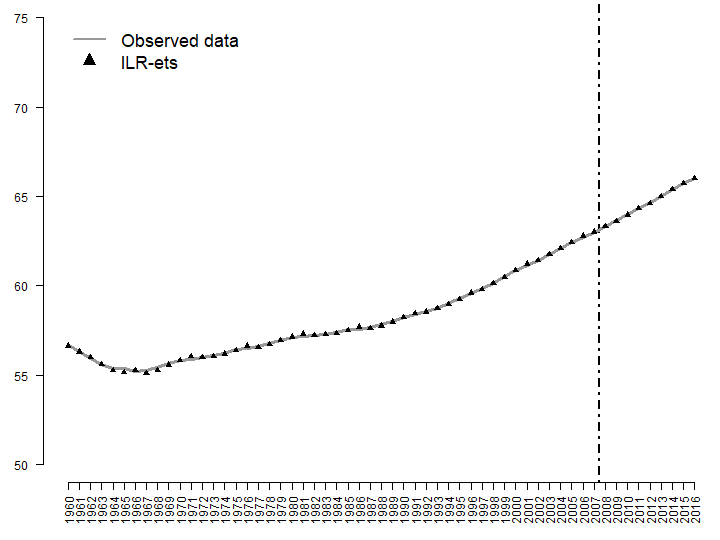

Supplement: S221 Fig — (PNG) [file pone.0212772.s221.png]

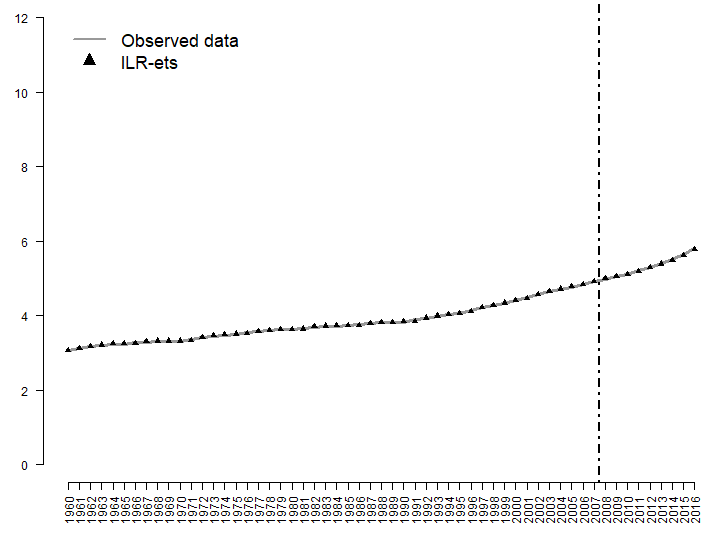

Supplement: S222 Fig — (PNG) [file pone.0212772.s222.png]

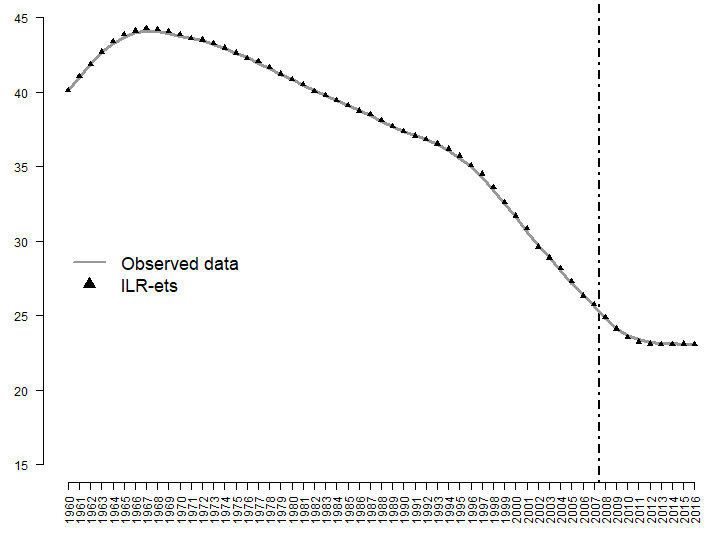

Supplement: S223 Fig — (PNG) [file pone.0212772.s223.png]

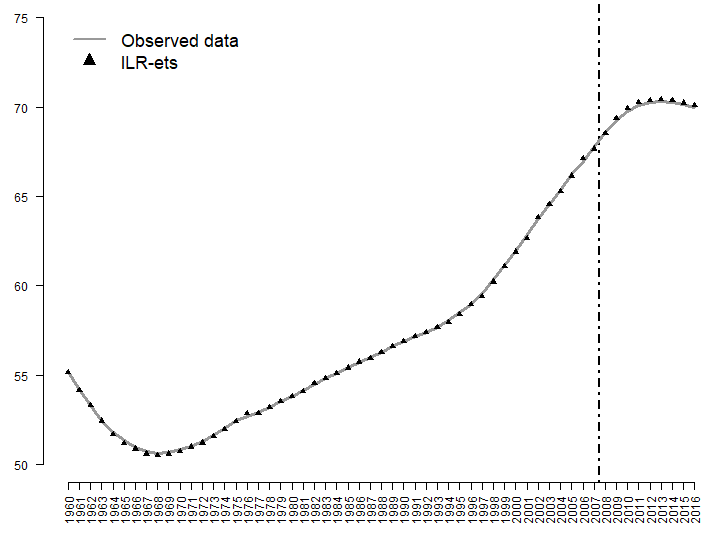

Supplement: S224 Fig — (PNG) [file pone.0212772.s224.png]

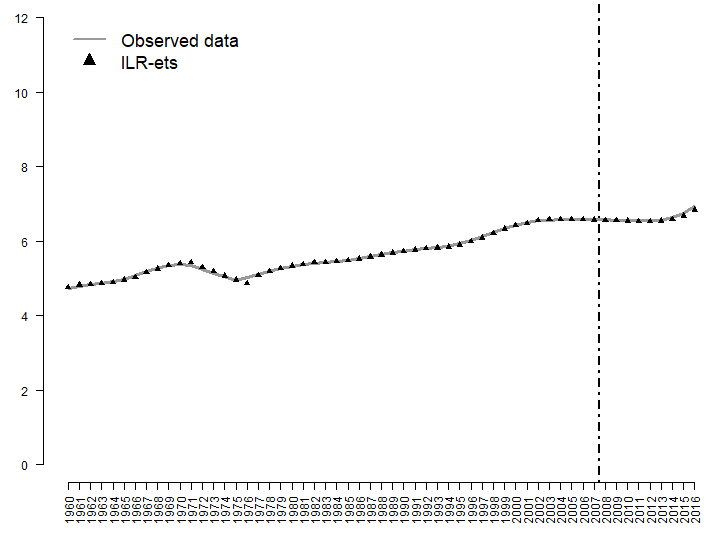

Supplement: S225 Fig — (PNG) [file pone.0212772.s225.png]
